# Insights into the Evolution of the Suppressors of Cytokine Signaling (SOCS) Gene Family in Vertebrates

**DOI:** 10.1093/molbev/msy230

**Published:** 2018-12-05

**Authors:** Bei Wang, Eakapol Wangkahart, Christopher J Secombes, Tiehui Wang

**Affiliations:** 1 Guangdong Provincial Key Laboratory of Pathogenic Biology and Epidemiology for Aquatic Economic Animal, Key Laboratory of Control for Disease of Aquatic Animals of Guangdong Higher Education Institutes, College of Fishery, Guangdong Ocean University, Zhanjiang, P.R. China; 2 Scottish Fish Immunology Research Centre, Institute of Biological and Environmental Sciences, University of Aberdeen, Aberdeen, United Kingdom; 3 Research Unit of Excellence for Tropical Fisheries and Technology, Division of Fisheries, Department of Agricultural Technology, Faculty of Technology, Mahasarakham University, Khamriang Sub-District, Kantarawichai, Mahasarakham, Thailand

**Keywords:** rainbow trout, SOCS gene family, synteny, phylogenetic tree, whole genome duplication, evolution, gene expression, ontogeny, cytokine and growth factor signaling

## Abstract

The SOCS family are key negative regulators of cytokine and growth factor signaling. Typically, 8–17 SOCS genes are present in vertebrate species with eight known in mammals, classified as type I (SOCS4–7) and type II (CISH and SOCS1–3) SOCS. It was believed that the type II SOCS were expanded through the two rounds of whole genome duplication (1R and 2R WGDs) from a single CISH/SOCS1–3 precursor. Previously, 12 genes were identified in rainbow trout but here we report 15 additional loci are present, and confirm 26 of the genes are expressed, giving rainbow trout the largest SOCS gene repertoire identified to date. The discovery of the additional SOCS genes in trout has led to a novel model of SOCS family evolution, whereby the vertebrate SOCS gene family was derived from CISH/SOCS2, SOCS1/SOCS3, SOCS4/5, SOCS6, and SOCS7 ancestors likely present before the two WGD events. It is also apparent that teleost SOCS2b, SOCS4, and SOCS5b molecules are not true orthologues of mammalian SOCS2, SOCS4, and SOCS5, respectively. The rate of SOCS gene structural changes increased from 2R vertebrates, to 4R rainbow trout, and the genes with structural changes show large differences and low correlation coefficient of expression levels relative to their paralogues, suggesting a role of structural changes in expression and functional diversification. This study has important impacts in the functional prediction and understanding of the SOCS gene family in different vertebrates, and provides a framework for determining how many SOCS genes could be expected in a particular vertebrate species/lineage.

## Introduction

The suppressors of cytokine signaling (SOCS) family of proteins are key negative regulators of cytokine and growth factor signaling. Since their discovery in the late 1990s, these small intracellular proteins have been characterized as regulatory cornerstones of intracellular signaling (Kazi et al. 2014; Linossi and Nicholson 2015; Jiang et al. 2017). In mammals, there are eight SOCS family members, cytokine-inducible SH2-containing protein (CISH) and SOCS1–7 (Delgado-Ortega et al. 2011; Linossi and Nicholson 2015; Mahony et al. 2016; Duncan et al. 2017). The SOCS family are characterized by a highly conserved C-terminal SOCS box motif, a central Src homology 2 (SH2) domain and an adjacent α-helical extension, termed the extended SH2-subdomain (ESS), and an N-terminal region that varies in sequence and length across the family (Delgado-Ortega et al. 2011; Linossi and Nicholson 2015; Duncan et al. 2017). The SOCS box acts as a substrate recognition module to mediate the polyubiquitination and subsequent degradation of substrate proteins by the 26S proteasome (1). The SH2 and ESS domain collectively bind tyrosine-phosphorylated motifs on target proteins. In addition, SOCS1 and SOCS3 have a unique kinase inhibitory region (KIR) that acts as a pseudosubstrate (Kershaw et al. 2013; Skjesol et al. 2014; Linossi and Nicholson 2015). SOCS4–7 contain an extensive N-terminal region (termed the type I subfamily) that distinguishes them from SOCS1–3 and CISH (type II subfamily; Jin et al. 2008).

The importance of SOCS regulation of immunological and other vital cellular responses is demonstrated by SOCS-deficient mice. SOCS1 knockout (KO) mice are perinatally lethal 2–3 weeks after birth due to the inflammation of several organs owing to IFNγ hyper-responsiveness (Naka et al. 1998; Marine, Topham, et al. 1999; Alexander et al. 1999). Both SOCS2 KO and transgenic mice show gigantism due to deregulated growth hormone signaling (Metcalf et al. 2000; Greenhalgh et al. 2002). SOCS3 KO and transgenic mice are embryonically lethal due to placental insufficiency or anemia, respectively (Marine, McKay, et al. 1999; Boyle and Robb 2008). SOCS4-deficient mice succumb to viral infection (Kedzierski et al. 2014), whereas SOCS5 transgenic mice inhibit interleukin (IL)-4 mediated STAT6 activation and reduce Th2 cell development (Seki et al. 2002). SOCS6 KO mice display growth retardation (Krebs et al. 2002) and SOCS7 KO mice are perinatally lethal due to growth retardation concomitant with hypoglycemia influenced by genetic background (Banks et al. 2005). Finally, CISH transgenic mice display impaired responses to IL-2 (Matsumoto et al. 1999).

The SOCS negative regulation is well documented in cytokines that mainly signal through the JAK/STAT pathway (Dogusan et al. 2000; Atanasova and Whitty 2012; Cianciulli et al. 2017). Tyrosine phosphorylation is one of the key events required to propagate signaling downstream of the JAK/receptor complex (Linossi and Nicholson 2015; Cianciulli et al. 2017). Many signaling proteins in these cascades contain phosphotyrosine-binding domains, such as an SH2 domain, allowing them to “dock” to this hub and carry out their function. The SOCS-SH2 domains bind to their targets only when the correct tyrosine is phosphorylated by active signaling. Phosphotyrosine-dependent binding of the SOCS-SH2 domain to its cognate target contributes to its ability to regulate signaling in two ways: firstly, localization to the correct target/receptor complex, which allows for the ubiquitination/inhibition of bound targets via the SOCS box, and secondly by competition with other signaling molecules, such as STATs, for the same phosphorylated site (1). In addition, SOCS1 and SOCS3 can directly inhibit JAK activation via their KIR, which positions in the substrate-binding pocket of JAK and blocks the access of incoming substrates (Kershaw et al. 2013). Similarly, SOCS proteins also negatively regulate growth factors that signal through receptors typically possessing a kinase domain (e.g., receptor tyrosine kinases or RTKs; Posner and Laporte 2010; Kazi et al. 2014).

SOCS proteins are constitutively expressed and act as physiological suppressors of cytokine and growth factor signaling (Krebs and Hilton 2000). Their expression can be upregulated by stimulation with cytokines and growth factors, and function as inducible negative feedback regulators (Kazi et al. 2014; Linossi and Nicholson 2015; Duncan et al. 2017). Other stimuli, including pathogen associated molecular patterns (PAMPs), chemokines and infections can also induce SOCS expression (Rakesh and Agrawal 2005; Wang, Gorgoglione, et al. 2011; Gorgoglione et al. 2013; Duncan et al. 2017). Dysregulation of SOCS gene expression leads to cancer and inflammatory, autoimmune, and neurodegenerative diseases (Liang et al. 2014; Jiang et al. 2017; Cianciulli et al. 2017).

From an evolutionary perspective, the SOCS family appears to have expanded to help regulate the increasingly complex JAK/STAT system (Dehal and Boore 2005; Liongue et al. 2012, 2016). Bioinformatics analysis has failed to identify a SOCS homologue in choanoflagellates and basic metazoa (ctenophores), although SOCS-box containing proteins are evident. However, homologues of mammalian SOCS1/2/3/CISH and SOCS6/7 were found in porifera, with an additional SOCS4/5 homologue found in cnidaria and maintained in the protostomia (e.g., Drosophila; Liongue et al. 2016). In the deuterostome sea squirt, a representative urochordate, the SOCS1/2/3/CISH and individual SOCS6 and SOCS7 proteins are present but the SOCS4/5 homologue was not found presumably as a result of gene loss in this lineage (Liongue et al. 2012). Liongue et al. hypothesized that the common ancestor of protostomes and deuterostomes possessed four members of the SOCS family: a SOCS1/SOCS2/SOSC3/CISH intermediate, a SOCS4/SOCS5 intermediate as well as distinct SOCS6 and SOCS7 precursors. The SOCS1/SOCS2/SOCS3/CISH lineage (type II SOCS) appears to follow the classical expansion during the two rounds (1R and 2R) of whole genome duplication (WGD) that occurred during early vertebrate evolution (Dehal and Boore 2005), generating SOCS1, SOCS2, SOCS3, and CISH, via SOCS1/SOCS3 and SOCS2/CISH intermediates. The SOCS4/SOCS5 intermediate expanded into SOCS4 and SOCS5 but no further copies were retained during 1R and 2R. In contrast, no expansion of the SOCS6 or SOCS7 genes has occurred, resulting in the eight SOCS members in mammals (Liongue et al. 2016).

The SOCS family in teleosts was first identified in several model fish species, that is, zebrafish *Danio rerio*, tetraodon *Tetraodon nigroviridis*, fugu *Fugu rubripes*, medaka *Oryzias latipes*, and stickleback *Gasterosteus aculeatus*, aided by their genome sequencing (Jin, Shao, et al. 2007; Jin, Xiang, et al. 2007; Jin et al. 2008). The SOCS family genes were later documented in rainbow trout *Oncorhynchus mykiss* (Wang and Secombes 2008; Wang, Gao, et al. 2010; Wang, Gorgoglione, et al. 2011; Maehr et al. 2014), turbot *Scophthalmus maximus* (Zhang et al. 2011), yellow perch *Perca flavescens* (Shepherd et al. 2012), catfish *Ictalurus punctatus* (Yao et al. 2015), tongue sole *Cynoglossus semilaevis* (Hao and Sun 2016), Japanese flounder *Paralichthys olivaceus* (Thanasaksiri et al. 2016), and Nile tilapia *Oreochromis niloticus* (Liu et al. 2016), with their function analyzed in a few species (Skjesol et al. 2014; Nie et al. 2014; Liu et al. 2016; Sobhkhez et al. 2017; Zhao et al. 2018). The orthologues of all the 8 mammalian SOCS family members have been found in teleosts with additional copies for CISH, SOCS1, 2, 3, 5, and 6 found in several fish species(Jin et al. 2008; Wang, Gorgoglione, et al. 2011; Yao et al. 2015; Thanasaksiri et al. 2016). Copy numbers of SOCS family genes are generally higher in fish genomes than in mammalian genomes. Additional SOCS members found in teleosts were believed to be mainly due to the fish wide WGD (3R) event or additional 4R WGD events in several fish lineages (Wang, Gorgoglione, et al. 2011; Yao et al. 2015; Thanasaksiri et al. 2016).

Rainbow trout is one of the most important Salmonid species for aquaculture, wild stock fisheries and recreational sport fisheries. Besides its economic importance, rainbow trout is also used extensively as a model species in a variety of biological disciplines such as comparative immunology. Many mammalian immune genes were found to have up to four copies in salmonids, for example, IL-1β (Husain et al. 2012), TNFα (Hong et al. 2013) due to the 4R WGD, which occurred 88–96 Ma in this lineage (Macqueen and Johnston 2014). The function of many fish cytokines, for example, IL-1β, TNFα, IFNγ, MCSF, IL-2, IL-4/13, IL-6, IL-8, IL-12, IL-15, IL-17A/F, IL-21, and IL-22, have been studied first in this species (Zou and Secombes 2016). Twelve SOCS family members have been documented in rainbow trout to date (Wang et al. 2008; Wang, Gao, et al. 2010; Wang, Gorgoglione, et al. 2011; Maehr et al. 2014). In the current study, 15 additional SOCS loci have been identified in the recently released rainbow trout genome (Berthelot et al. 2014), with 14 loci cloned at the cDNA level. Thus, rainbow trout possesses 27 SOCS genes (two copies of SOCS1 and SOCS4, three copies of SOCS3, and four copies of SOCS2, SOCS5, SOCS6, SOCS7, and CISH), the most SOCS genes encountered so far in any organism. Furthermore, our bioinformatics analysis reported here suggests that the duplicated SOCS2 and SOCS5 genes known in teleosts predate the 3R WGD, which impacts on the theory of when SOCS gene duplications occurred in early vertebrates. We next systematically studied the expression of all the trout SOCS gene family in vivo in tissues of healthy fish, and during ontogeny. This study provides novel insights into the expansion, evolution and functional diversification of the SOCS gene family in vertebrates, and sets the foundation for future functional studies of these important regulators in fish immune responses.

## Results

### Cloning and Characterization of the SOCS Gene Family in Rainbow Trout

Extensive analysis of EST, TSA and WGS databases identified 27 loci (table 1) in the rainbow trout genome. Twelve loci have been cloned previously as SOCS1a (originally SOCS1), SOCS2a1, SOCS2b1, SOCS2b2, SOCS3a (originally SOCS3), SOCS5b1 (originally SOCS9), SOCS6a (originally SOCS6), SOCS7a1 (originally SOCS7), and four CISH paralogues (Wang and Secombes 2008; Wang, Gao, et al. 2010; Wang, Gorgoglione, et al. 2011; Maehr et al. 2014). Fourteen loci, SOCS1b, SOCS2a2, SOCS3b1-2, SOCS4, SCOS5a1-2, SOCS5b2, SOCS6a2, SOCS6b1-2, SOCS7a2, and SOCS7b1-2, have been cloned from cDNA in this study (table 1, supplementary figs. S1–14, Supplementary Material online).

**Table 1.** Summary of Sequence Analysis of SOCS Gene Family in Rainbow Trout.

Mammals	3R Fish	Rainbow Trout	mRNA	Genomic Contigs	Chromosome	No of Exons	Coding Exon	Amino Acids (aa)	Molecular Weight (kDa)	pI
CISH	CISHa	CISHa1	AM903340^a^	CCAF010023507	7	3	3	225	24.85	9.44
		CISHa2	HG003693^a^	CCAF010055202	17	3	3	225	24.89	9.85
	CISHb	CISHb1	FR873795^a^	CCAF010044231	7	3	3	233	25.76	8.04
		CISHb2	HG003694^a^	CCAF010010398	17	3	2	203	22.71	7.69
				CCAF010010399						
SOCS1	SOCS1a	SOCS1a	AM748721^a^	MSJN01099356	UK	3	2	253	27.90	**9.02**
	SOCS1b	SOCS1b	KY387584	MSJN01004860	17	3	2	300	34.68	**6.00**
SOCS2	SOCS2a	SOCS2a1	AM748722^a^	CCAF010046190	15	4	2	201	22.71	**8.61**
		SOCS2a2	KY387585	CCAF010046784	21	3	2	201	22.50	**6.91**
				CCAF010188627						
	SOCS2b	SOCS2b1	FR874096^a^	CCAF010036701	16	4	2	218	24.55	**9.48**
		SOCS2b2	FR874097^a^	CCAF010027937	9	3	2			
SOCS3	SOCS3a	SOCS3a	AM748723^a^	CCAF010078215	13	2	1	212	23.81	9.30
	SOCS3b	SOCS3b1	KY387586	CCAF010092130	23	2	2	228	24.58	9.04
		SOCS3b2	KY387587	CCAF010048864	20	2	2	225	24.24	8.37
SOCS4	SOCS4	SOCS4	KY387588	CCAF010102522	25	2	1	398	45.05	9.25
				CCAF010102521						
		SOCS4b		CCAF010092130	19	?	1	462	52.97	8.29
SOCS5	SOCS5a	SOCS5a1	KY387589	CCAF010017338	1	2	1	562	61.99	**8.73**
				CCAF010017342						
		SOCS5a2	KY387590	CCAF010029944	23	2	1	567	62.69	**8.30**
	SOCS5b	SOCS5b1	AM903341^a^	CCAF010050454	24	2	1	544	60.57	**6.66**
		SOCS5b2	KY387591	CCAF010049242	27	2	1	550	61.06	**6.56**
				CCAF010049241						
SOCS6	SOCS6a	SOCS6a1	AM903342^a^	CCAF010004893	28	2	1	515	58.10	**6.07**
		SOCS6a2	KY387592	CCAF010023265	8	2	1	513	57.95	**6.07**
	SOCS6b	SOCS6b1	KY387593	CCAF010075589	15	3	1	543	60.91	**7.72**
		SOCS6b2	KY387594	CCAF010031173	11	2	1	608	67.61	**6.04**
SOCS7	SOCS7a	SOCS7a1	AM903343^a^	CCAF010053000	13	10	9	652	71.49	**6.66**
				CCAF010052999						
				CCAF010052998						
		SOCS7a2	KY387595	CCAF010023137	12	10	9	702	76.39	**5.95**
				CCAF010153388						
				CCAF010181790						
				CCAF010105492						
	SOCS7b	SOCS7b1	KY387596	CCAF010024912	17	9	9	837	90.36	**7.15**
		SOCS7b2	KY387597	CCAF010034763	13	9	9	841	90.82	**7.14**

a cDNA sequences reported previously (Wang and Secombes 2008; Wang, Gao, et al. 2010; Wang, Gorgoglione, et al. 2011; Maehr et al. 2014).

A second SOCS4 locus (SOCS4b) was predicted that has the same two-exon structure as SOCS4 and shares 83.2% identity in the overlapping N-terminal but with an extended C-terminal after the SOCS box due to a deletion of 4 bp leading to an open reading frame (ORF) shift and reading through the stop codon (supplementary fig. S15, Supplementary Material online). However, PCR using primers designed at the predicted first and last exons or at the junction across the intron yielded no products from cDNA samples prepared from 17 tissues, from cell lines (RTS-11, RTG-2) and from primary cultures of HK cells, splenocytes and HK macrophages. This suggests that the SOCS4b locus is not active in rainbow trout.

**Fig. 1.**
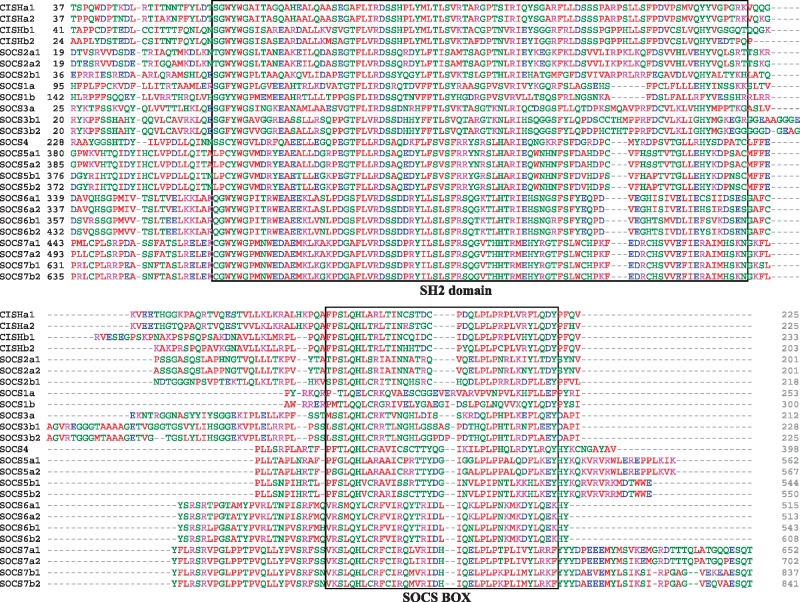
Multiple alignment of the rainbow trout SOCS family. The multiple alignment was produced using ClustalW. The accession numbers of the amino acid sequences used are as in figures 2 and 3. SOCS2b2 and SOCS4b were excluded from the alignment due to a premature stop (SOCS2b2, Wang, Gorgoglione, et al. 2011) or extended C-terminal (SOCS4). The C-terminal of the alignment containing the SH2 domain and the SOCS box is shown.

The SOCS molecules identified and cloned in rainbow trout are summarized in table 1. All the trout SOCS genes except SOCS1a can be located at a specific chromosome, with the 4R WGD paralogues on different chromosomes. The predicted proteins differ in size (from 201 to 841 aa) and some of the isoforms encoded by paralogues show disparate pIs, for example, SOCS1a and SOCS1b, SOS2a1 and SOCS2a2, SOCS5a and SOCS5b, and SOCS7a and SOCS7b (table 1). All the trout SOCS proteins contain a well-conserved SH2 domain and a SOCS box at the C-terminal (fig. 1). Trout SOCS4, SOCS5, and SOCS7 have longer C-terminals after the SOCS box, as is typical in other species (Kazi et al. 2014; Linossi and Nicholson 2015).

### Evolutionary Analysis of the SOCS Gene Family in Vertebrates

**Fig. 2.**
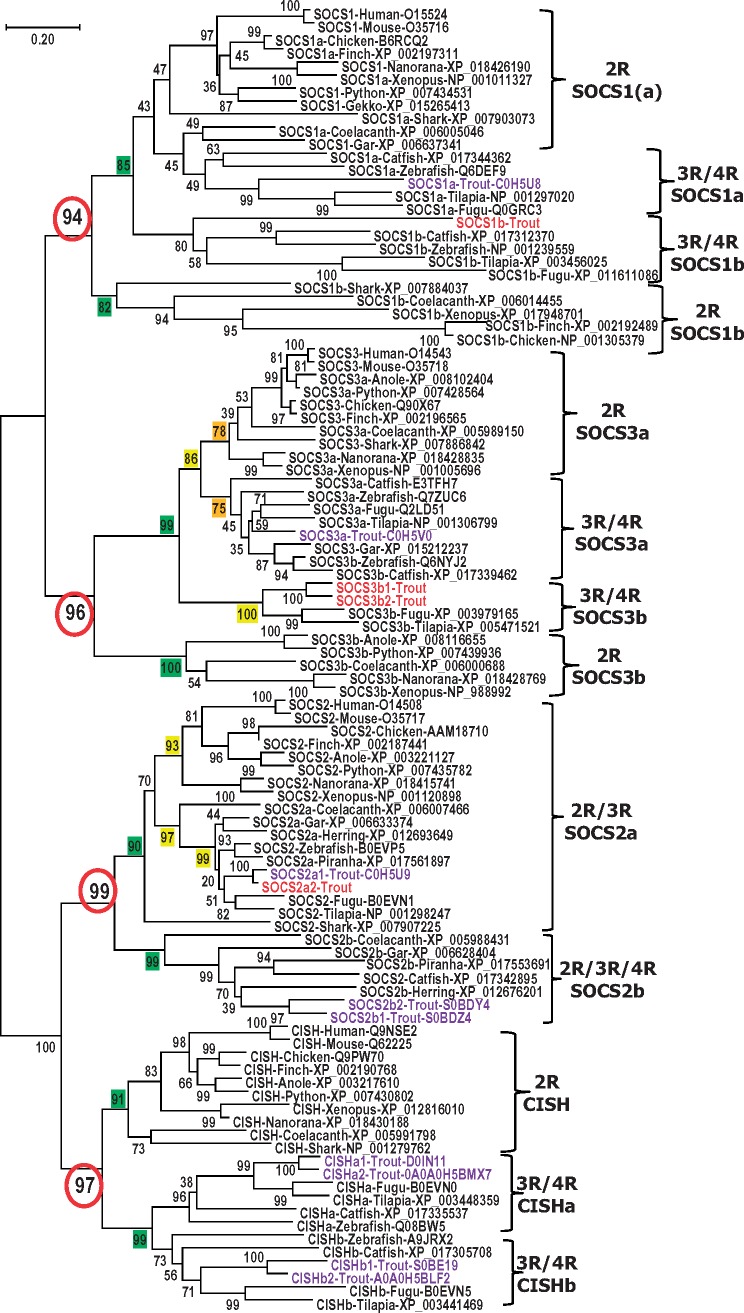
Phylogenetic tree analysis of vertebrate type II SOCS (SOCS1-3 and CISH). The phylogenetic tree was constructed using amino acid multiple alignments generated by ClustalW and the Neighbor-Joining method within the MEGA7 program (68). The percentage of replicate trees in which the associated taxa clustered together in the bootstrap test (10,000 replicates) is shown next to the branches. The evolutionary distances were computed using the JTT matrix-based method with the pairwise deletion option. The accession number of each sequence is shown after the common species name. The trout sequences reported in this study are in red and those known previously in purple.

Eight SOCS genes are present in mammals, and up to 12 SOCS genes have been reported in some individual fish species (Yao et al. 2015). With 27 loci in the genome, rainbow trout contains the largest number of SOCS genes found in any organism analyzed to date (table 2). To test the hypothesis (Jin et al. 2008; Liongue et al. 2012, 2016) of vertebrate SOCS gene family evolution, that proposes the expansion of SOCS family genes is due to the 1R and 2R WGD events resulting in the eight SOCS gene family members in mammals, and an increased number in teleost fish mainly due to the fish specific 3R WGD and further 4R WGD in certain fish lineages such as the salmonids, we analyzed the SOCS gene family throughout the vertebrates. Up to 15 SOCS genes are present in 3R teleosts with additional SOCS3a and SOCS5a present in zebrafish and catfish (table 2). We identified 9 SOCS genes in birds and a 2R ray-finned fish (spotted gar), 10 SOCS genes in amphibians, reptiles and a cartilaginous fish (elephant shark), and 12 SOCS genes in lobe-finned fish (coelacanth; table 2). The identities of these SOCS molecules were confirmed by phylogenetic analysis, where each of the SOCS members from different lineages was grouped and formed an independent clade with high bootstrap support, as shown in the Neighbor-Joining phylogenetic trees (figs. 2 and3). Similar phylogenetic tree topologies have also been obtained using Maximum Likelihood (ML) and Minimum Evolution (ME) methods (supplementary figs. S16–19, Supplementary Material online). Moreover, each pair of trout SOCS paralogues reported here grouped together first, and are on different chromosomes in the genome (table 1), suggesting a 4R WGD origin. Further examination of the phylogenetic tree and homology analysis of the SOCS protein sequences revealed that whereas CISH, SOCS1, SOCS2a, SOCS3, SOCS5a, SOCS6, and SOCS7 identified in teleosts are orthologues of mammalian counterparts, teleost SOCS2b, SOCS4, and SOCS5b appear to predate the 3R WGD as described below.

**Table 2.** Comparison of Identified SOCS Gene Copy Numbers Across Different Vertebrate Lineages.

Lineage	Salmonids	3R Ray-finned Fish	2R Ray-finned Fish	Cartilaginous Fish	Lobe-finned Fish	Amphibians	Reptiles	Birds	Mammals
Species^a^	Rainbow trout	Fugu, Tilapia, Zebrafish, catfish	Spotted gar	Elephant shark	Coelacanth	Tropical clawed frog, *Nanorana parker*i	Green Anole, Burmese python	Chicken, zebra finch	Human, mouse
Genome duplication	4R	3R	2R	2R	2R	2R	2R	2R	2R
CISH	4	2	^b^	1	1	1	1	1	1
SOCS1	2	2	1	2	2	2	1	2	1
SOCS2	4	2	2	1	2	1	1	1	1
SOCS3	3	2 (3)	1	1	2	2	2	1	1
SOCS4	2	1	1	1	1	1	1	1	1
SOCS5	4	2 (3)	2	2	2	1	2	1	1
SOCS6	4	2	1	1	1	1	1	1	1
SOCS7	4	2	1	1	1	1	1	1	1
Total	27^c^	15 (17)^d^	9	10	12	10	10	9	8

a The main species analyzed are listed. In the absence/incompleteness of relevant sequences, a sequence from a phylogenetically related species was used for analysis.

b CISH has not been identified in spotted gar yet or is not present.

c There are 26 transcribed genes and one predicted gene SOCS4b known in rainbow trout.

d Zebrafish and catfish possess additional SOCS3a and SOCS5a paralogues compared with other 3R ray-finned fish.

**Fig. 3.**
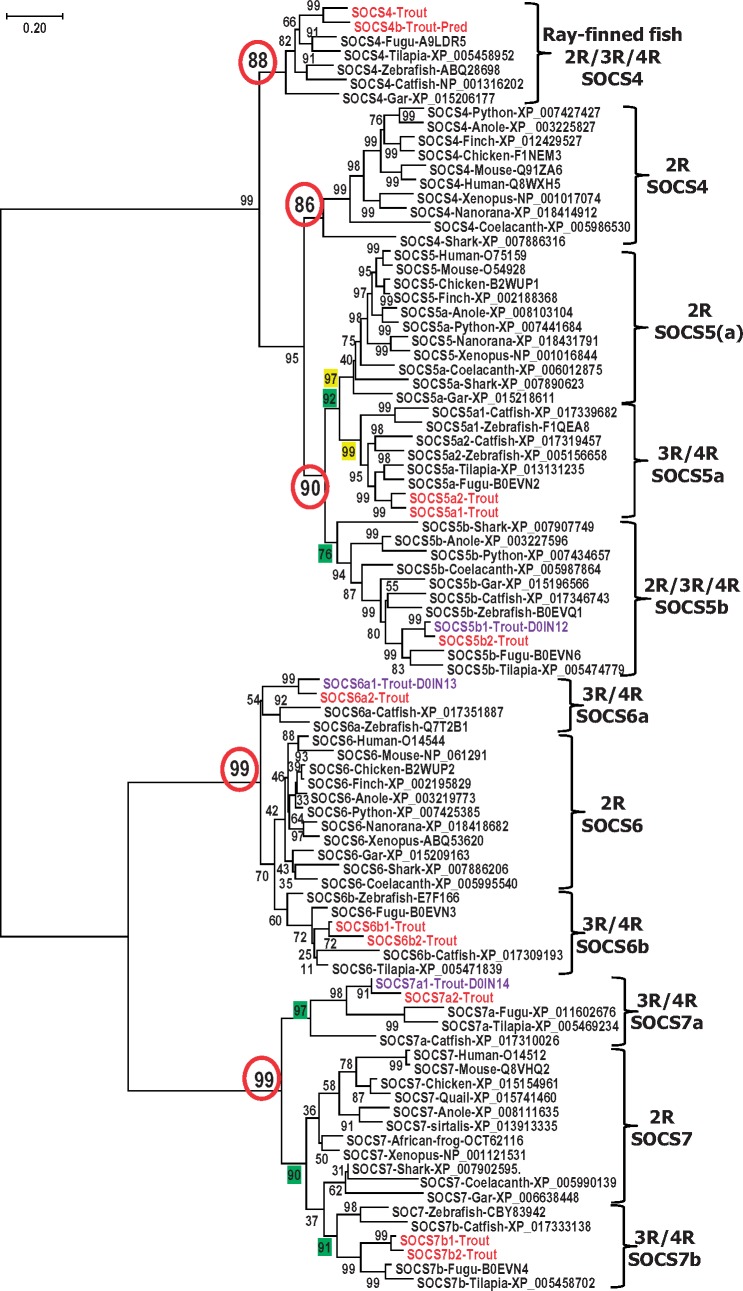
Phylogenetic tree analysis of vertebrate type I SOCS (SOCS4–7). The phylogenetic tree was constructed using amino acid multiple alignments generated by ClustalW and the Neighbor-Joining method within the MEGA7 program. The percentage of replicate trees in which the associated taxa clustered together in the bootstrap test (10,000 replicates) is shown next to the branches. The evolutionary distances were computed using the JTT matrix-based method with the pairwise deletion option. The accession number of each sequence is shown after the common species name. The trout sequences reported in this study are in red and those known previously in purple.

#### The Evolutionary Analysis of Type II SOCS Genes in Vertebrates

Type II SOCS genes arose from a common CISH/SOCS1/2/3 ancestor that existed in invertebrates, via proposed SOCS1/3 and SOCS2/CISH intermediates, leading to the four type II SOCS genes in mammals and other vertebrates (Liongue et al. 2012, 2016), that was presumed to have happened during the two rounds of WGD that occurred in early vertebrates. The discovery, reported here, of SOCS1b, SOCS2b, and SOCS3b in 2R vertebrates, including cartilaginous, ray-finned and lobe-finned fish species, as well as amphibians, reptiles and birds (table 2, fig. 2, supplementary figs. S16–19, Supplementary Material online) suggests that the intermediates SOCS1/3 and SOCS2/CISH in fact predate the first (R1) WGD. Hence, the two rounds of WGD potentially produced two copies (a and b) each of SOCS1–3 and CISH in 2R vertebrates, with some of the paralogues subsequently lost in a lineage-specific way (supplementary fig. S20, Supplementary Material online).

##### CISH

Only one CISH was found in 2R vertebrates with the exception of 2R spotted gar in which no CISH has been identified to date. Two CISH paralogues, CISHa and CISHb, have been found in several 3R teleosts and four in 4R salmonids. The fish CISHa and CISHb share higher homology between fish orthologues but similar homology to 2R vertebrate CISH, for example, 37.8–47.8% aa identities between CISHa and CISH and 35.1–46.9% between CISHb and CISH (supplementary table S2, Supplementary Material online). In phylogenetic tree analysis, CISHa and CISHb formed independent clades first and grouped with 2R vertebrate CISH with high bootstrapping support (fig. 2, supplementary figs. S16 and 17, Supplementary Material online). Fish CISHa and CISHb loci, as well as tetrapod CISH loci were found syntenically conserved (Wang, Gorgoglione, et al. 2011). All of this evidence suggests that teleost CISHa and CISHb are true orthologues of CISH present in 2R vertebrates and arose from the 3R/4R WGDs (supplementary fig. S20*B*, Supplementary Material online). The homology and phylogenetic tree analysis also suggests that fish CISHa and CISHb diverged symmetrically.

##### SOCS1

Two SOCS1 paralogues, SOCS1a and SOCS1b, are found in 3R/4R ray-finned fish, as well as 2R cartilaginous and lobe-finned fish, amphibians and birds, but only one SOCS1 could be detected in 2R ray-finned fish, reptiles and mammals (table 2). The gar SOCS1 shares high aa identities to mammalian SOCS1 (50.7–56.6%) and other 2R vertebrate SOCS1a (48.8–59.1%), compared with 2R vertebrate SOCS1b (28.3–33.9%; supplementary table S3, Supplementary Material online), suggesting that the gar SOCS1 is a true orthologue of mammalian SOCS1 or 2R vertebrate SOCS1a (supplementary fig. S20*C*, Supplementary Material online). It is noteworthy that 3R/4R fish SOCS1b share higher aa identities to SOCS1a (25.3–44.6%) and mammalian SOCS1 (24.1–43.6%) compared with 2R vertebrate SOCS1b (20.1–37.9%, supplementary table S3, Supplementary Material online). In phylogenetic tree analysis, the 3R/4R fish SOCS1b grouped with SOCS1a and mammalian SOCS1 (including the gar SOCS1) first, with 2R vertebrate SOCS1b forming a sister clade (fig. 2, supplementary figs. S16 and 17, Supplementary Material online). Furthermore, the fish SOCS1a and SOCS1b loci, as well as mammalian SOCS1 loci were found syntenically conserved (Yao et al. 2015). All these suggest that 3R/4R fish SOCS1 paralogues arose via 3R WGD, and are orthologues of 2R vertebrates SOCS1a or mammalian/gar SOCS1 (supplementary fig. S20*C*, Supplementary Material online).

##### SOCS2

Two SOCS2 paralogues, SOCS2a and SOCS2b, are found in several 2R/3R ray-finned fish, as well as 2R lobe-finned fish, with four in salmonids (table 2). The 2R vertebrate SOCS2a share higher aa identities to 3R/4R fish SOCS2a (55.6–81.7%), compared with SOCS2b (32.8–46.3%; supplementary table S4, Supplementary Material online). Vice versa, the 2R vertebrate SOCS2b share higher aa identities to 3R/4R fish SOCS2b (38.9–55.2%), compared with SOCS2a (36.9–46.7%). In phylogenetic tree analysis, the 3R/4R fish SOCS2a grouped with 2R vertebrate SOCS2a and SOCS2 and formed a sister clade with SOCS2b from 2R/3R/4R vertebrates (fig. 2, supplementary figs. S16 and 17, Supplementary Material online). Such data suggest that the SOCS2a and SOCS2b paralogues arose via the 2R WGD, and were further expanded in salmonids via the 4R WGD (supplementary fig. S20*D*, Supplementary Material online). Interestingly, only a single SOCS2 was found in shark that grouped in the SOCS2a clade in agreement with homology analysis (supplementary table S4, Supplementary Material online).

##### SOCS3

Only one SOCS3 was found in mammals, birds, gar and elephant shark but two or more were found in other vertebrates (table 2). The gar SOCS3 shares high aa identities to mammalian SOCS3 (63.0–69.2%) and other 2R vertebrates SOCS3a (64.9–80.1%), as well as 3R/4R teleost SOCS3b (49.6–52.8%), compared with 2R vertebrate SOCS3b (34.9–41.0%; supplementary table S5, Supplementary Material online), suggesting that the gar SOCS3 is a true orthologue of mammalian SOCS3 or 2R vertebrate SOCS3a (supplementary fig. S20*E*, Supplementary Material online). It is noteworthy that 3R/4R fish SOCS3b shares higher aa identities to SOCS3a (43.0–56.7%) and SOCS3 (43.3–52.8%) compared with 2R vertebrate SOCS3b (32.1–39.0%, supplementary table S5, Supplementary Material online). In phylogenetic tree analysis, the 3R/4R fish SOCS3b grouped with a sister group containing SOCS3a and mammalian SOCS3 (including the gar SOCS3) first, with 2R vertebrate SOCS3b forming a sister clade (fig. 2, supplementary figs. S16 and 17, Supplementary Material online). Furthermore, the fish SOCS3a and SOCS3b loci, as well as mammalian SOCS3 loci were found syntenically conserved (Yao et al. 2015). This suggests that the 3R/4R fish SOCS3 paralogues arose via the 3R WGD, and are orthologues of 2R vertebrate SOCS3a or mammalian/gar SOCS3 (supplementary fig. S20*E*, Supplementary Material online). The homology and phylogenetic tree analysis also suggests that fish SOCS3a and SOCS3b diverged asymmetrically.

#### The Evolutionary Analysis of Type I SOCS Genes in Vertebrates

##### SOCS4

Only a single SOCS4 is present in 2R/3R vertebrates except in rainbow trout in which two loci are present in the genome due to the 4R WGD. The SOCS4 molecules share high aa identities in ray-finned fish (56.87–81.9%) and in tetrapods (68.5–90.8%; supplementary table S6, Supplementary Material online). However, the SOCS4 from cartilaginous and lobe-finned fish share higher aa identities to that of tetrapods (54.5–61.3%) than to ray-finned fish (38.1–45.7%). The relatedness of fish and tetrapod SOCS4 was evidenced by the somewhat conserved synteny (Wang, Gorgoglione, et al. 2011; Yao et al. 2015), and analysis in this study (fig. 3). The ray-finned fish SOCS4 forms an independent group, as does the SOCS4 from tetrapods, cartilaginous and lobe-finned fish, that group with SOCS5 first (fig. 3, supplementary figs. S18 and 19, Supplementary Material online). It is noteworthy that the ray-finned fish SOCS4 are shorter (384–398 aa, with the exception of the predicted trout SOCS4b that has an extended C-terminal as described above) than SOCS4 from other 2R vertebrates (426–438 aa). Taken as a whole, the ray-finned fish SOCS4 and SOCS4 from other 2R vertebrates may have evolved separately from the two SOCS4 paralogues that arose from the 2R WGD (supplementary fig. S21*A*, Supplementary Material online).

##### SOCS5

Two or more SOCS5 paralogues are found in ray-finned, lobe-finned and cartilaginous fish, as well as reptiles (table 5). Two SOCS5a paralogues, due to chromosome duplication, in addition to SOCS5b, are present in zebrafish and catfish (41). The 3R/4R teleost SOCS5a shares higher aa identities to 2R vertebrate SOCS5a (59.6–72.0%) and SOCS5 (59.9–66.0%), compared with SOCS5b (41.9–48.1%; supplementary table S7, Supplementary Material online). Similarly, the 3R/4R teleost SOCS5b shares higher aa identities to 2R vertebrate SOCS5b (45.2–69.1%) compared with SOCS5 and SOCS5a (41.9–48.2%; supplementary table S7, Supplementary Material online). In phylogenetic tree analysis, the 3R/4R fish SOCS5a grouped with 2R vertebrate SOCS5a and SOCS5 and formed a sister clade with SOCS5b from 2R/3R/4R vertebrates (fig. 3, supplementary figs. S18 and 19, Supplementary Material online). This suggests that the 2R/3R SOCS5a and SOCS5b paralogues arose via the 2R WGD, and were further expanded in salmonids via the 4R WGD (supplementary fig. S21*B*, Supplementary Material online).

##### SOCS6

Only a single SOCS6 was found in 2R vertebrates, with 2 paralogues in 3R teleosts and 4 in 4R salmonids (table 2). The increase of SOCS6 in teleosts coincides with the 3R and 4R WGDs (supplementary fig. S21*C*, Supplementary Material online). The genomic loci of teleost SOCS6a and SOCS6b, as well as tetrapod SOCS6 loci are syntenically conserved (41). The 2R vertebrate SOCS6 share similar aa identities to the 3R/4R fish SOCS6a (60.6–78.1%) and SOCS6b (61.7–77.2%; supplementary table S8, Supplementary Material online). Moreover, all SOCS6 molecules grouped together with high bootstrap support (fig. 3, supplementary figs. S18 and 19, Supplementary Material online). Taken as a whole, the expansion of SOCS6 in teleosts appears due to the 3R and 4R WGDs.

##### SOCS7

**Fig. 4.**
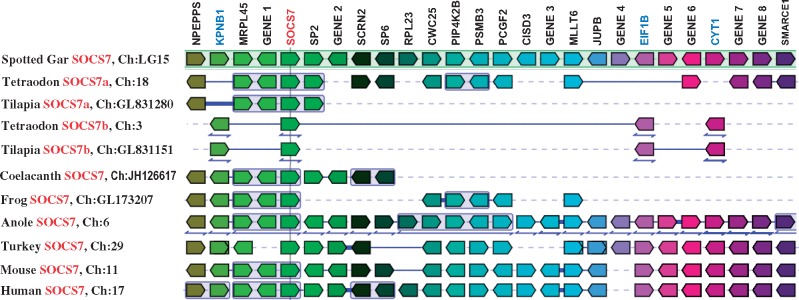
PhyloView to show gene synteny at the SOCS7 loci across the bony vertebrates. The synteny was analyzed with Genomicus v92.01 using the gene order of the spotted gar SOCS7 locus as a reference. The syntenically conserved orthologues or gene blocks are shown in matching colors. A line between two genes is equivalent to a break in the continuity of the alignment. A thin double-headed arrow under a block of genes indicates that the order of the genes shown was reversed. Gene 1 = si: ch211-156l18.8, Gene 2 = ENSLOCG00000013725, Gene 3 = ENSLOCG00000013683, Gene 4 = si: dkey-28e7.3, Gene 5 = zgc: 110712, Gene 6 = ENSLOCG00000013624, Gene 7 = ENSLOCG00000013599, and Gene 8 = ENSLOCG00000013584.

Only a single SOCS7 has been reported previously in a number of fish species (Jin et al. 2008; Wang, Gao, et al. 2010; Yao et al. 2015; Hao and Sun 2016). Three SOCS7 paralogues, SOCS7a2, SOCS7b1, and SOCS7b2, have been cloned in rainbow trout in this study. Further BLAST search identified two SOCS7 paralogues in several 3R fish (i.e., catfish, fugu, tetraodon, and tilapia) but only one SOCS7 in spotted gar and other 2R vertebrates (table 2). The 2R vertebrate SOCS7 share similar aa identities to the ray-finned fish SOCS7a (30.0–47.4%) and SOCS7b (33.5–50.9%; supplementary table S9, Supplementary Material online). In the phylogenetic tree, all SOCS7 molecules grouped together with high bootstrap support (fig. 3, supplementary figs. S18 and 19, Supplementary Material online). Moreover, the genomic loci of teleost SOCS7a and SOCS7b, as well as SOCS7 loci of other 2R vertebrates, are syntenically conserved (fig. 4). The coincidence of an increase in SOCS7 paralogues in 3R teleosts and 4R salmonids with the 3R and 4R WGDs (supplementary fig. S21*D*, Supplementary Material online) is clear.

#### A Model of SOCS Gene Family Evolution in Vertebrates

**Fig. 5.**
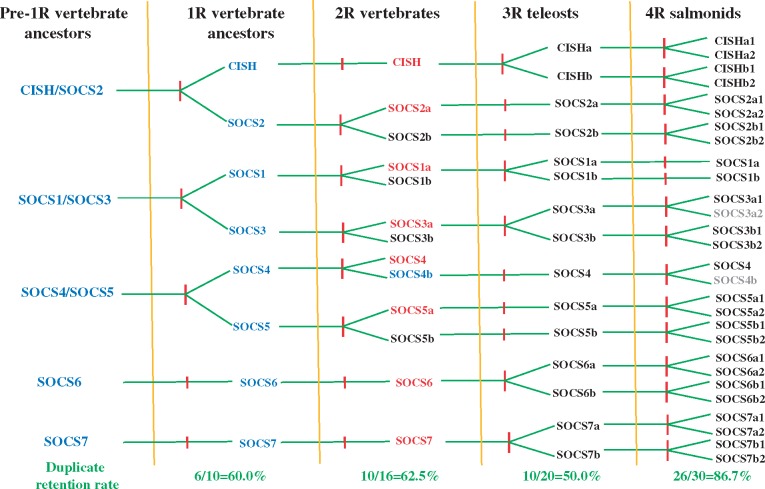
A model for the evolution of the SOCS family in vertebrates. An evolutionary timeline relevant to the whole genome duplications (1R, 2R, 3R, 4R) is indicated with vertical red lines. The evolutionary intermediates are in blue and mammalian SOCS family members in red. The gene names in grey indicate absent or inactive paralogues.

The SOCS family in extant vertebrate species has evolved from SOCS1/SOCS2/SOCS3/CISH and SOCS4/SOCS5 intermediates as well as distinct SOCS6 and SOCS7 precursors, through two rounds of WGDs (Jin et al. 2008; Liongue et al. 2012). On the basis of phylogenetic tree, homology, and synteny analysis (above), a model of the evolution of SOCS family molecules in vertebrates is proposed (fig. 5) that is different from Jin’s and Liongue’s model. First, the CISH/SOCS2 and SOCS1/SOCS3 intermediates likely pre-existed the 1R WGD, and were present with the SOCS4/SOCS5 intermediate and SOCS6 and SOCS7 precursors. The 1R WGD appears to have given rise to CISH and SOCS1-5, with one of the duplicated SOCS6-7 lost, and these were the ancestors of SOCS family members seen in mammals. Whereas one copy of the 2R duplicated CISH, SOCS6, and SOCS7 was lost in all 2R vertebrates, the second copies of SOCS1–5 were lost in a lineage specific manner leading to eight SOCS genes in mammals and up to 12 genes in other 2R vertebrates, for example, in coelacanth (table 2). The second copies of SOCS1 and SOCS3, and the SOCS4 copy of 2R vertebrates appear to have been lost in the ancestor of ray-finned fish. The 3R WGD duplicates of CISH, SOCS1, SOCS3, SOCS6, and SOCS7 are retained, but one of the duplicated SOCS2a, SOCS2b, SOCS4, SOCS5a, and SOCS5b was lost, resulting in up to 15 SOCS genes in some 3R fish species, which were further increased by gene duplication in a species-specific way (table 2). All these 3R SOCS genes were duplicated by a subsequent 4R WGD in salmonids with the exception of SOCS1a and SOCS1b where the other copy generated by this WGD was lost (fig. 5). The second copies of SOCS3a and SOCS4 may also have been lost or are inactive in some salmonids, as seen in rainbow trout where 26 expressed SOCS genes are present. In conclusion, whereas the CISH, SOCS1, SOCS2a, SOCS3, SOCS5a, SOCS6, and SOCS7 in teleosts are true orthologues of mammalian CISH and SOCS1–3, 5–7, respectively, the true orthologues of 3R/4R teleost SOCS2b, SOCS4, and SOCS5b arose from a copy generated from the 2R WGD, with the other copy giving rise to mammalian SOCS2, SOCS4, and SOCS5 genes. The retention rate for each WGD elaborated on this model is 60.0%, 62.5%, 50.0%, and 86.7% for 1R, 2R, 3R, and 4R, respectively.

In summary, this novel SOCS evolution model differs from Jin’s and Liongue’s model (Jin et al. 2008; Liongue et al. 2012, 2016) in the following ways: 1) there were 5 instead 4 SOCS genes in pre1R vertebrate ancestors; 2) there were 8, instead 6 SOCS members present in 1R vertebrate ancestors; 3) there are 12 instead of eight SOCS members retained in current 2R vertebrates; and 4) there are 15 SOCS members in 3R fish with SOCS2b, SOCS4, and SOCS5b arising from the other 2R copy relative to mammalian counterparts.

### Gene Organization of SOCS Gene Family

**Fig. 6.**
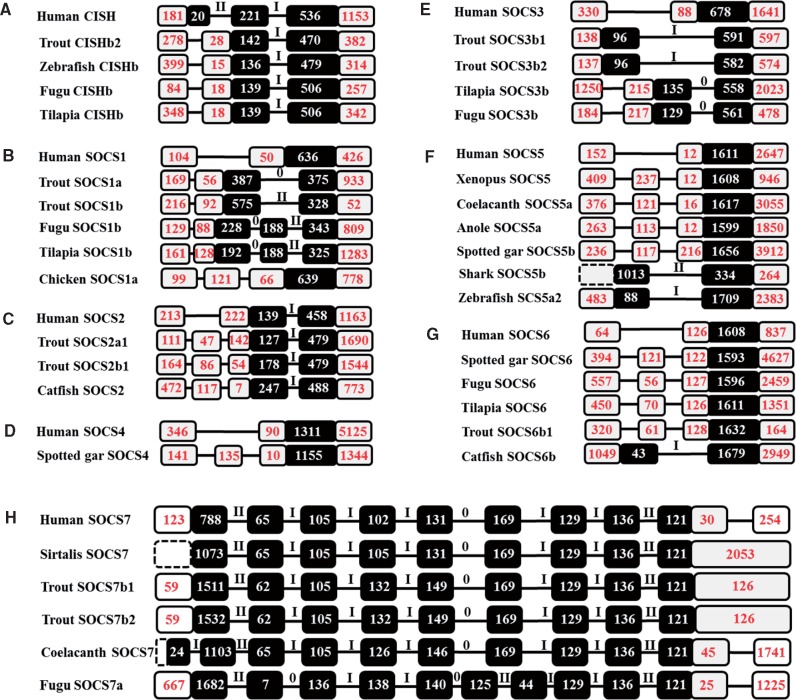
Diversification of gene organization of CISH (*A*), SOCS1 (*B*), SOCS2 (*C*), SOCS4 (*D*), SOCS3 (*E*), SOCS5 (*F*), SOCS6 (*G*), and SOCS7 (*H*) in vertebrates. The black and white boxes represent amino acid coding regions and untranslated regions within exons, respectively, and the black bars represent introns. The sizes (bp) of exons are numbered in the boxes and the intron phases are indicated above the bar. Dashed boxes indicate uncertainty of size. Gene organizations that differ from the human genes are shown. Detailed gene organization analysis of each SOCS family member is provided in supplementary figures S22–29, Supplementary Material online.

The eight SOCS genes in mammals have a characteristic exon/intron structure (Wang, Gorgoglione, et al. 2011). CISH and SOCS2 possess a three exon/two intron structure, with all three exons encoding for CISH, but only the last two encoding for SOCS2. SOCS1 and 3–6 each have two exons, with the first exon noncoding, whereas SOCS7 has 10 exons with the last exon noncoding. The diversified repertoire of the SOCS family in different vertebrate lineages prompted us to examine the exon/intron structure of SOCS genes. A general characteristic of mammalian SOCS gene organization can be observed in different vertebrate lineages, but diversification of exon/intron structure, in terms of number of exons, coding exons and intron phase, was found in paralogues in a species/lineage specific manner, as shown in supplementary figures S22–29, Supplementary Material online and summarized in figure 6.

All vertebrate CISH genes possess three exons, and all three exons are coding except for trout CISHb2, and zebrafish, fugu and tilapia CISHb in which only the last two exons are coding (fig. 6*A* and supplementary fig. S22, Supplementary Material online). All SOCS2 genes possess three exons, with the last two coding except for trout SOOCS2a1 and SOCS2b1, and catfish SOCS2 that had an exon insertion in the 5′-UTR (fig. 6*C* and supplementary fig. S24, Supplementary Material online). Trout SOCS2b2 may represent an expressed decaying SOCS2 paralogue that has a phase 0 intron encoding a mutant SOCS2 protein. Most of the vertebrate SOCS1, SOCS3, SOCS4, SOCS5, and SOCS6 genes have two exons with only the last exon coding (supplementary figs. S23, S25–S28, Supplementary Material online). An exception is the intron insertion in the coding region of SOCS1 (trout SOCS1a and SOCS1b, and fugu and tilapia SOCS1b, supplementary fig. S23, Supplementary Material online), SOCS3 (trout SOCS3b1 and SOCS3b2, fugu and tilapia SOCS3b, supplementary fig. S25, Supplementary Material online), SOCS5 (zebrafish SOCS5a2 and shark SOCS5b, supplementary fig. S27, Supplementary Material online), and catfish SOCS6b (supplementary fig. S28, Supplementary Material online). It is noteworthy that the position of the intron insertion in the coding exon is random, resulting in different intron phases, for example, intron phase 0 for trout SOCS1a versus II for trout SOCS1b, and intron phase I for trout SOCS3b1 and SOCS3b1 versus 0 for tilapia and fugu SOCS3b. Another exception is the exon insertion in the 5′-UTR in chicken SOCS1b, spotted gar SOCS4, SOCS5 (xenopus SOCS5, coelacanth and anole SOCS5a, and spotted gar SOCS5b) and SOCS6 (spotted gar, fugu and tilapia SOCS6, and trout SOCS6b; fig. 6, supplementary figs. S23, S26–S28, Supplementary Material online). The ten-exon SOCS7 structure is well conserved in vertebrates. This conservation includes the last noncoding exon, with conserved sizes of 105 bp, 169 bp, 129 bp, and 136 bp for exons 3, 6, 7, and 8, respectively, and conserved intron phases. Exceptions include the intron insertion in coelacanth SOCS7 exon 1, fugu SOCS7 exon 6, and intron lose in the 3′-UTR in sirtalis SOCS7 and trout SOCS7b1 and SOCS7b2 (fig. 6*H*, supplementary fig. S29, Supplementary Material online). The fugu SOCS7a is unique in having a small exon 2 (7 bp vs. 53–65) and large exon 3 (136 bp vs. 105 bp) with an intron in phase 0 (vs. I) and a phase II intron insertion in the conserved exon 6 (fig. 6*H*).

In conclusion, diversification of the gene organization of vertebrate SOCS family members has been observed in a gene- and lineage/species-specific manner, as demonstrated by the loss of coding capacity in exon 1 of CISH, the exon insertion in the 5′-UTR, random intron insertion in coding exons, and the loss of an intron in the 3′-UTR of SOCS7. The percentage of genes with a change of gene organization was 10.2%, 22.4%, and 42.3% (supplementary table S10, Supplementary Material online) in 2R vertebrates, 3R fish, and 4R rainbow trout, respectively.

### Constitutive Expression of SOCS Family Members in Rainbow Trout Tissues and in RTS-11 Cells

To understand the biology of the SOCS gene family and the paralogues in healthy fish and during development, their expression was comparatively studied. The relative expression of each SOCS gene in 17 tissues and in RTS-11 cells (Ganassin and Bols 1998) is presented in supplementary figure S30, Supplementary Material online (with data on CISH published previously (Maehr et al. 2014)). Tissue and gene specific expression patterns were apparent. The expression of type II SOCS (SOCS1, 2, and 3 paralogues) was diverse and ranged from low arbitrary units (AU < 10) to medium levels (10 < AU < 1000) with at least one paralogue expressed at high levels (AU > 1000) in some tissues (supplementary fig. S30*A*–*C*, Supplementary Material online). The expression of SOCS4 was low (AU < 10, supplementary fig. S30*D*, Supplementary Material online) and was only detectable in four out of seventeen tissues. The expression level of other type I SOCS (SOCS5, 6, and 7 paralogues) was also low (AU < 10) to medium (10 < AU < 1000) with the exception of SOCS7 paralogues in the brain which had high expression levels (AU > 1000; supplementary fig. S30*E*–*G*, Supplementary Material online). The expression level of most of the CISH paralogues was medium to high in tissues (Maehr et al. 2014).

Paired samples *T* tests indicated that the expression levels of different SOCS paralogues were largely different in tissues and RTS-11 cells (supplementary table S11, Supplementary Material online). However, the expression levels of paralogues were correlated with the exception of SOCS2a1 and SOCS2b1 (supplementary table S12, Supplementary Material online). It is noticeable that the correlation coefficient (R) was higher between 4R than 3R paralogues with the exception of CISH and SOCS7b paralogues, and the type I SOCS (5–7) were highly correlated (supplementary table S12, Supplementary Material online). Interestingly, the correlation coefficient R of expression levels between paralogues of SOCS2a1, SOCS2b1 and SOCS6b1 and SOCS7b was relatively low (supplementary table S12, Supplementary Material online).

**Fig. 7.**
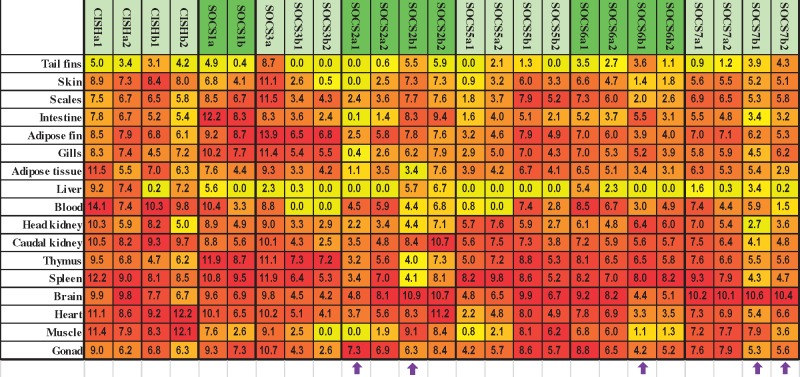
A heat map of SOCS gene expression in rainbow trout. The transcript expression level of each SOCS gene was determined by real time RT-PCR in 17 tissues from six fish. The transcript level was calculated using a serial dilution of references that contained equal molar amounts of the probes for each gene and was normalized against the expression level of EF-1α. The expression level was expressed as arbitrary units (AU) where 1 AU equals the expression level of EF-1α divided by 1,000,000. The average AU of each SOCS gene across tissues were log2 transformed and a heat map was generated with the highest expression in red and lowest expression in yellow for each gene. Zero represents an expression level with AU equal to or below 1. Arrows indicate genes with intron insertion in the 5′-UTR or intron loss in the 3′-UTR.

To give an insight into the potential function of SOCS genes at a tissue level, the complex gene expression levels were converted to a heat-map (fig. 7). The expression of SOCS4 was low and is not included further. The expression of most of the SOCS genes was low in the surface tissues/organs such as tail fins, skin, scales, intestine, adipose fin and gills, and the internal tissues/organs liver, head kidney (HK) and adipose tissue, but medium to high levels were apparent in the brain as well as in immune organs such as the thymus and spleen. In general, the expression of type I SOCS and SOCS2 was low in immune tissues/organs but the other type II SOCS were highly expressed in a tissue specific manner (fig. 7). It is noticeable that the expression patterns of SOCS2a1, SOCS2b1 and SOCS6, which had an exon insertion in the 5′-UTR, and SOCS7b1 and SOCS7b2, which lost an intron in the 3′-UTR, showed greater differences for their paralogues (fig. 7).

### Expression of SOCS Family Members during Ontogeny in Rainbow Trout

**Fig. 8.**
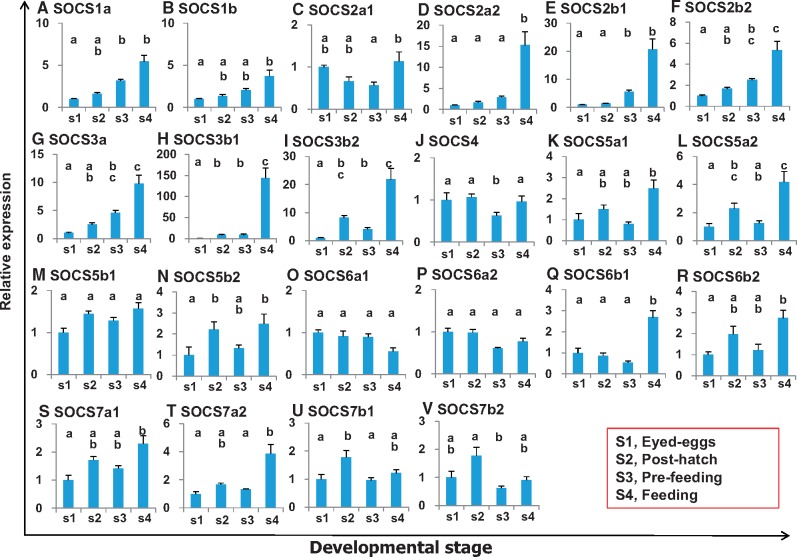
The ontogeny of trout SOCS gene expression. cDNA samples were prepared from eyed eggs (S1), immediately post-hatching (S2), pre-first feeding fry (S3), or fry 3 weeks after first feeding (S4). Six independent samples for each developmental stage were prepared for real-time quantification of gene expression. The expression level was normalized to that of the housekeeping gene EF-1α from the same sample, and expressed as arbitrary units where one unit equals the average expression level of each gene in eyed eggs. The results are presented as averages + SEM (*N* = 6). The expression levels between different developmental stages are statistically significant (*P* ≤ 0.05) where letters over the bars are different, as determined by one-way ANOVA.

The in vivo ontogenetic expression of all trout SOCS genes was examined at four early life stages during development (fig. 8). The egg stage had amongst the lowest expression level of all SOCS genes with the exception of SOCS5b1, SOCS6a1, and SOCS6a2, which stayed at similar levels in all stages; and SOCS4, which also stayed at similar levels but with a drop in pre-feeding fry (fig. 8*J*, *M*, *O*, and *P*). The expression of all other SOCS genes was, in general, similar or gradually increased from the eyed egg stage to pos-thatching and the pre-feeding fry, and reached the highest expression level in post-feeding fry (fig. 8). It is noteworthy that the expression changes were small in type I SOCS genes with less than a 4-fold increase from eyed eggs to post-feeding fry (fig. 8*J*–*V*). In contrast, some type II SOCS genes showed a more dramatic increase, for example, SOCS3b1 which increased >100-fold from eyed eggs to post-hatching fry, and SOCS2a2, SOCS2b1, SOCS3a, and SOCS3b2 which increased >10-fold (fig. 8*A*–*I*). The expression of CISH paralogues followed the pattern of other type II SOCS genes with an increase from eyed eggs to postfeeding fry, as reported previously (Maehr et al. 2014).

## Discussion

### The SOCS Gene Family in Rainbow Trout and the Evolution of the SOCS Gene Family

Despite many functional studies and important roles of the eight SOCS gene family members in mammals (Linossi and Nicholson 2015), our current knowledge of the SOCS gene repertoire in other vertebrates and how SOCS genes evolved across vertebrates is still rudimentary. In this study, 27 genomic loci of the SOCS gene family have been identified in the rainbow trout genome, with 26 genes confirmed to be expressed. Compared with eight SOCS genes in mammals and up to 17 genes in other vertebrates, rainbow trout has the largest repertoire of SOCS genes identified in any organism to date. Furthermore, we show that in 2R vertebrates up to 12 SOCS genes may be present, with a second SOCS1, SOCS2, SOCS3, and SOCS5 detectable, and that in 3R teleosts 15 SOCS family genes can be identified, with some additional gene duplications in particular species. Hence, this study provides a framework for determining how many SOCS genes could be expected in a particular vertebrate species/lineage, and proposes a new model for SOCS gene evolution.

The vertebrate SOCS gene family was derived from CISH/SOCS1–3, SOCS4/5, SOCS6, and SOCS7 ancestors in early vertebrates via WGD events in line with the expansion of JAK/STAT pathways (Liongue et al. 2012). Our bioinformatics analysis suggested for the first time the presence of CISH/SOCS2 and SOCS1/SOCS3 ancestral genes, rather than a single CISH/SOCS1–3 intermediate (Liongue et al. 2012, 2016), in early vertebrate ancestors before the first WGD. This provides an evolutional mechanism as to how additional SOCS1–3 paralogues evolved in 2R vertebrates (other than in mammals) via the two WGDs. Further WGDs and lineage/species specific gene loss/retention has resulted in the SOCS gene repertoires observed today. Our analysis also suggests that teleost SOCS2b, SOCS4, and SOCS5b are not true orthologues of mammalian SOCS2, SOCS4, and SOCS5, respectively. This may suggest novel functional roles of these genes in different vertebrates. For example, mammalian SOCS4 is broadly expressed in the hematopoietic system and a critical regulator of antiviral immunity (Kedzierski et al. 2014). SOCS4 expression is hardly detectable in the rainbow trout hematopoietic system (this study) and in catfish (Yao et al. 2015), suggesting that fish SOCS4 is unlikely to have a major role in immunity as seen in mammals. Similarly, SOCS2b and SOCS5b may possess novel functions unknown in mammalian SOCS2 and SOCS5.

WGDs, a sudden doubling of the complete genome, have markedly impacted vertebrate evolution and represent important evolutionary landmarks from which some major lineages have diversified (Lien et al. 2016; MacKintosh and Ferrier 2018). Gene balance hypothesis predict that copy numbers of genes encoding multiple protein structures and pathways must be kept in a constant ratio to avoid architectural disruption or metabolic imbalance (Pires and Conant 2016). Thus, genes encoding regulatory proteins that form oligomers, that interact transiently with multiprotein complexes in regulatory pathways are preferentially co-retained after WGDs. Accordingly, the SOCS family proteins, via their SOCS-box and SH domains, interacting with multiple substrates and modulating multiple signal pathways, are expanded via WGD. However, the retention of SOCS paralogues is member and WGD specific. It appears that only the progeny duplicates from CISH/SOCS2, SOCS1/SOCS3, and SOCS4/SOCS5, but not SOCS6 and SOCS7, were retained after the 1R and 2R WGDs. The 2R paralogues SOCS2a/b, SOCS4, and SOCS5a/b remained as single copies in 3R teleosts, whereas the 4R duplicates in rainbow trout are all retained with the exception of SOCS1a/b. This suggests that novel mechanisms other than gene balance might be in operation after different WGDs to preserve SOCS paralogues. The higher retention rate in 4R salmonids relative to 3R teleosts may be contributed by the short evolution time after WGD. The rise of 2R vertebrate CISH and SOCS1–5 is in line with the expansion of the JAK/STAT pathways (Liongue et al. 2012). Further WGDs in teleosts and salmonids expanded the SOCS gene family along with the signal pathways of cytokines and growth factors, which may generate parallelized signaling networks. By sub/neofunctionalization of the paralogues, the parallel signaling pathways can evolve specific regulatory interconnections (MacKintosh and Ferrier 2018) that integrate multiple inputs of cytokines and growth factors, and generate a wider repertoire of phenotypic outcomes of defense and growth.

### Lineage/Species-Specific Diversification of Exon/Intron Structure of SOCS Genes

Change of exon/intron structure might have an impact on gene expression and function (Xu et al. 2012; Jo and Choi 2015; Sajjanar et al. 2017). Loss of coding capacity, exon insertion/gain in the 5′-UTR, independent intron insertion(s)/gain in the coding regions, and intron loss in the 3′-UTR, were all observed in SOCS genes in a lineage/species-specific manner. Loss of coding capacity of CISH in several fish species shortened the N-terminal of the proteins encoded, and may have direct effects on their function. UTRs of a transcript play significant roles in translation regulation (Sajjanar et al. 2017). Exon insertions have been found in the 5′-UTR of SOCS1–2, 4–6 in some species that may bring extra control elements such as internal ribosome entry sites, upstream ORFs, terminal oligopyrimidine tracts and secondary structure (Sajjanar et al. 2017) in the 5′-UTR. AU-rich elements, microRNA response elements and other regulatory elements in the 3′-UTR also play important roles in mRNA turnover, a critical component of translation regulation (Sajjanar et al. 2017). The exon insertion in the 5′-UTR and loss of an intron in the 3′-UTR will potentially impact functional diversification of the SOCS genes affected.

All eukaryotic genomes carry introns, for example, in humans, introns constitute 25% of the genome, some 4–5 times the size of the exons (Jo and Choi 2015). Introns provide selective advantages to eukaryotic cells, such as regulating alternative splicing, enhancing gene expression, controlling mRNA transportation, chromatin assembly and regulation of nonsense-mediated decay (Jo and Choi 2015). Intron insertion in the coding region was found in SOCS1, 3, and 5–7. Exon insertion in the 5′-UTR also resulted in novel intron sequences, suggesting that intron insertion may play an important role in SOCS gene diversification.

Large-scale analysis suggests that structural divergences (changes of exon/intron structure) are prevalent in duplicated genes compared with orthologues and, in many cases, have led to the generation of functionally distinct paralogues (Xu et al. 2012). The structural change rate is proportional to evolutionary time (Xu et al. 2012). Unexpectedly, only 10.2% of SOCS genes in 2R vertebrates experienced structural changes, but this rate increased to 22.4% in 3R teleosts and 42.3% in 4R rainbow trout. This may suggest a relaxation of selective pressures in 3R and 4R paralogues that may accelerate functional diversification.

### Tissue Specific Expression Patterns

Although SOCS gene expression has been investigated in some fish species, in whole fish or a limited number of tissues (Yao et al. 2015; Hao and Sun 2016; Thanasaksiri et al. 2016), the current report is the first study to compare the complete repertoire of SOCS family genes (26 members in trout) in 17 tissues by real-time PCR in a single species. Each SOCS gene is differentially expressed in a tissue-specific manner. In adult fish, most of trout SOCS genes are lowly expressed in surface tissues and in liver, HK and adipose tissue, but highly expressed in the brain, spleen and thymus. The surface tissues are sites of pathogen attack and environmental assault. The liver is an important immune organ that responds to food borne pathogens and toxins. Fish HK is a major site of hematopoiesis, analogous to the mammalian bone marrow and a key secondary immune organ. The adipose tissue is increasingly seen as playing an important role in immune function, and can influence and be influenced by adjacent and embedded immune cells that patrol the internal organs. Since SOCS proteins are negative regulators of cytokine and growth factor signaling, the low-level expression in these tissues will allow prompt immune activation. The high-level expression of SOCS in brain, spleen and thymus perhaps prevents excessive cytokine and growth factor signaling to maintain homeostasis. The type I SOCS and SOCS2 expression is low in most immune tissues/organs whereas other type II SOCS expression is high in a SOCS- and tissue-specific manner. These expression patterns suggest that each SOCS protein is spatially positioned to regulate the cytokine and growth factor signaling networks, with type I SOCS more oriented to growth and type II SOCS to immune function (Wang, Gorgoglione, et al. 2011; Kazi et al. 2014; Linossi and Nicholson 2015; Duncan et al. 2017). Consistent with this notion is the finding that no or only minor changes of expression were observed during ontogeny in type I SOCS in contrast to type II SOCS, which increased >10-fold (>100-fold for SOCS3b1) from eyed eggs to feeding fry. Fish, including salmonids, have critical early stages of development, particularly from hatching to feeding when the protection provided by the eggshell is lost and water and food borne pathogens are met directly for the first time in life that will activate the host immune response. The increased expression of type II SOCS may represent an immunoregulatory mechanism to prevent host damage and autoimmunity.

### Differential Expression, Gene Structure, and Functional Diversification of SOCS Paralogues

The SOCS paralogues typically show differential expression spatially in tissues and in RTS-11 cells but the expression levels are correlated, suggesting sub/neofunctionalization. The correlation efficient is in general higher between 4R paralogues than paralogues derived from 3R or an earlier origin (between SOCS2a/b and SOCS5a/b) as expected. However, the correlation coefficient between 4R CISHa, CISHb, and SOCS7b paralogues is lower than that between their 3R paralogues, indicating a fast diversification.

The expression patterns of SOCS2a1, SOCS2b1, SOCS6, SOCS7b1, and SOCS7b2 showed large differences relative to their paralogues, and the correlation coefficient of expression levels was low between these genes and their paralogues. Interestingly, SOCS2a1, SOCS2b1, and SOCS6 had an exon insertion in the 5′-UTR, and SOCS7b1, and SOCS7b2 lost an intron in the 3′-UTR. This suggests that gene structure changes, such as intron insertion in the 5′UTR and intron loss in the 3′-UTR, potentially have impacts on gene expression, leading to functional diversification of the paralogues.

### Conclusion

Prior to this study, 8–17 SOCS genes were known to be present in different vertebrate species. Thus, identification of 26 expressed genes in rainbow trout makes this the largest repertoire of SOCS genes in any organism to date. Our bioinformatics analysis suggests that 2R vertebrates may possess up to 12 and 3R teleosts up to 15 SOCS family genes, with additional genes potentially arising from species/lineage-specific gene duplication. A novel model for SOCS family evolution is presented whereby the vertebrate SOCS genes were derived from CISH/SOCS2, SOCS1/SOCS3, SOCS4/5, SOCS6, and SOCS7 ancestors in early vertebrate ancestors via WGDs. Our analysis also proposes that teleost SOCS2b, SOCS4, and SOCS5b are not true orthologues of mammalian SOCS2, SOCS4, and SOCS5, respectively. The appearance of 2R vertebrate CISH and SOCS1–5 is in line with the expansion of the JAK/STAT pathways. Further WGDs in teleosts and salmonids expanded the SOCS gene family along with the signaling pathways of cytokines and growth factors, and this may have resulted in parallelized signaling networks. This study provides a framework for determining how many SOCS genes could be expected in a particular vertebrate species/lineage.

The SOCS paralogues in trout show differential expression spatially in tissues but the expression levels are correlated, suggesting sub/neofunctionalization. Several changes in gene structure were noted, that increased with WGDs. The genes with such changes showed more distinct tissue expression patterns and a low correlation efficient between paralogues, suggesting that gene structure change may accelerate functional diversification. Each SOCS gene was differentially expressed in a tissue-specific manner, presumably to allow spatially positioned SOCS proteins to optimally regulate the cytokine and growth factor signaling networks in trout.

## Materials and Methods

### Identification and Cloning of the SOCS Family Genes in Rainbow Trout

A BLAST (the basic local alignment search tool; Altschul et al. 1990) search was performed at NCBI (http://blast.ncbi.nlm.nih.gov/Blast.cgi, last accessed July 2018) using known SOCS protein sequences from rainbow trout and other species, resulting in the identification of EST (expressed sequence tags), TSA (transcriptome shotgun assembly) and WGS (whole genome shotgun) contigs for 27 SOCS gene loci (table 1) in the trout genome (Berthelot et al. 2014). Twelve loci matched the 12 trout SOCS family members published previously (Wang and Secombes 2008; Wang, Gao, et al. 2010; Wang, Gorgoglione, et al. 2011; Maehr et al. 2014). The coding region of the other 15 loci was predicted as described previously (Wang et al. 2015, 2016, 2018). Primers (supplementary table S1, Supplementary Material online) were designed at the predicted 5′-untranslated region (UTR) and 3′-UTR to PCR amplify the complete ORF using a mixed cDNA sample from different tissues, leading to the cloning of 14 of these SOCS genes (loci) in rainbow trout. PCR using primers designed at the predicted SOCS4b locus or at the junction across the predicted intron yielded no products from cDNA prepared from 17 tissues, from cell lines (RTS-11, RTG-2) and from primary cultures of HK cells, splenocytes and HK macrophages. Cloning, sequencing and protein sequence analysis was performed as described previously (Wang, Diaz-Rosales, et al. 2011; Hong et al. 2013). Programs used included: the AlignIR program (LI-COR, Inc.) for nucleotide sequence analysis, the Splign program (https://www.ncbi.nlm.nih.gov/sutils/splign/splign.cgi, last accessed July 2018) for gene organization predication, the ClustalW program (Chenna et al. 2003) for multiple sequence alignments, the MatGAT program (V2.02, Campanella et al. 2003) for global sequence comparisons and SMART7 (Letunic et al. 2012) for SH2 and SOCS domain prediction. The trout SOCS genes were mapped to chromosomes using the genome assembly GCA_002163495.1.

### Evolutionary Analysis of SOCS Gene Family

The protein sequences of the SOCS gene family were extracted from Expasy and NCBI databases from representative model species of different vertebrate lineages with their genomes sequenced. The main species analyzed are elephant **shark***Callorhinchus milii* (cartilaginous fish); **coelacanth***Latimeria chalumnae* (lobe-finned fish); spotted **gar***Lepisosteus oculatus* (2R ray-finned fish); **fugu**, **tilapia**, **zebrafish**, and **catfish** (3R ray-finned fish), rainbow **trout** (4R ray-finned fish); tropical clawed frog ***Xenopus****tropicalis* and ***Nanorana****parkeri* (amphibians); green **anole***Anolis carolinensis* and Burmese **python***Python bivittatus* (reptiles); **chicken***Gallus gallus* and zebra **finch***Taeniopygia guttata* (Birds); and **human***Homo sapiens* and **mouse***Mus musculus* (mammals). SOCS genes from other species including Atlantic **herring***Clupea harengus*, red-bellied **piranha***Pygocentrus nattereri*, common garter snake *Thamnophis****sirtalis*** and Japanese **quail***Coturnix japonica* were also used in the analysis when a SOCS sequence was absent or incomplete in a model species.

The same set of protein sequences were used for homology and phylogenetic analysis. Homology analysis was performed using MatGAT with Blossom6.2 matrix, and a penalty of 10 for gap opening and 1 for gap extension. Phylogenetic trees were constructed using a multiple alignment and the Neighbor-Joining method within the Molecular Evolutionary Genetics Analysis program (MEAG, version 7; Kumar et al. 2016). The evolutionary distances were computed using the JTT matrix-based method. The pairwise-deletion option was used for the NJ and ME tree construction, and bootstrap tested for 10,000 (NJ), 5,000 (ME), and 1,000 (ML) times.

### Comparative Expression Analysis of Trout SOCS Gene Family Members

#### Real-Time PCR Analysis of Gene Expression

Primer design, quality control and real-time RT-PCR analysis were performed as described previously (Wang, Diaz-Rosales, et al. 2011; Hong et al. 2013) using a LightCycler480 Instrument II (Roche). At least one primer was designed across an intron and tested to ensure that no amplification from genomic DNA (200 ng per reaction) was observed for a specific primer pair. The primer pairs to differentiate different paralogues were designed manually based on a multiple cDNA sequence alignment of all paralogues. At least one primer for any one paralogue was able to distinguish this transcript from the remaining paralogues by ensuring that the 3′-end nucleotide of the primer is different from the other paralogues and at least one more nucleotide at the 3′-end region (nucleotides 2 to 5 from the 3′-end) differs from the others. The cp (crossing point) value increased by at least 10 with plasmid templates when primers of the other paralogue(s) were used, suggesting that the rate of cross amplification between paralogues is below 1/1,000. A common reference containing an equimolar amount of purified PCR products representing the 26 actively expressed trout SOCS genes and the house keeping gene elongation factor-1α (EF-1α) was used for quantification. Primers used for real-time PCR detection are detailed in supplementary table S1, Supplementary Material online.

#### Tissue Distribution of Gene Expression

Rainbow trout were purchased from the Mill of Elrich Trout Fishery (Aberdeenshire, UK) and maintained in 1 m diameter, aerated fiberglass tanks supplied with a continuous flow of recirculating freshwater at 14 ± 1 °C in the aquarium facility at the Scottish Fish Immunology Research Centre, University of Aberdeen. Fish were fed twice daily on standard commercial pellets (EWOS), and were acclimated for at least 2 weeks prior to use.

Six healthy rainbow trout (mean ± SEM = 142 ± 9 g) were anaesthetized, killed and seventeen tissues in the order of blood, thymus, gills, tail fins, adipose fin, scales, skin, muscle, spleen, liver, adipose tissue, heart, ovary, HK, caudal kidney, intestine, and brain, were sampled. The RNA preparation and RT-PCR analysis was performed as described previously (Hong et al 2013). In all cDNA samples, the expression of each gene was calculated relative to the expression level of EF-1α, and multiplied by 1,000,000 to give an arbitrary unit for each sample. Six cDNA samples prepared from unstimulated RTS-11 cells (a trout macrophage-like cell line) were also included in the analysis. The expression of each SOCS gene was presented as mean ± SEM (*N* = 6). The average expression levels of each SOCS gene across tissues were log2 transformed and a heat map was generated with the highest expression in red and lowest expression in yellow for each gene.

#### Ontogeny of the Expression of the SOCS Gene Family

To investigate if the expression of SOCS is correlated to immune capacity in early life, the ontogeny of the expression of SOCS genes was examined. Juvenile stages of rainbow trout were raised at 10 °C in recirculated water in the Institut National de la Recherche Agronomique’s experimental fish facility, Jouy-en-Josas, as described previously (Wang, Monte, et al. 2010). Eyed eggs (Stage S1, ∼280 degree days, DD), immediate pos-thatch fry (S2, ∼370 DD), pre-first feeding (PFF) fry (S3, 560 DD), at the stage of full disappearance of the yolk sac, and fry 3 weeks following first feeding (S4, 770 DD) were sampled. The RNA preparation and cDNA synthesis was as described previously (Wang, Diaz-Rosales, et al. 2011; Hong et al. 2013). Six samples for each developmental stage were prepared. To obtain enough RNA, each sample contained two eyed eggs (S1) or two larvae (S2) at hatching, but a single PFF or feeding fry was sufficient. The real-time quantification of gene expression was as described above. The comparative expression level of each gene was expressed relative to the expression level in eyed eggs (arbitrary unit = 1).

### Statistical Analysis

Real-time PCR data were analyzed using the SPSS Statistics package 24.0 (SPSS Inc., Chicago, IL), as described previously (Wang, Diaz-Rosales, et al. 2011). A paired samples *T* test was applied to the expression levels between SOCS paralogue pairs (supplementary fig. S30, Supplementary Material online). One way-analysis of variance (ANOVA) and the LSD post hoc test was used to analyze the ontogeny of expression data in figure 8, with *P* ≤ 0.05 between groups considered significant.

## Supplementary Material

Supplementary data are available at *Molecular Biology and Evolution* online.

## Supplementary Material

Supplementary Data
