# Supplementary material for: Insights into the Evolution of the Suppressors of Cytokine Signaling (SOCS) Gene Family in Vertebrates
Source: Mol Biol Evol. 2018 Dec 5;36(2):393–411. doi: 10.1093/molbev/msy230 (PMC6368001; doi:10.1093/molbev/msy230)
Supplement: Supplementary Data [file msy230_supp.docx]

**Supplementary materials for**

**Insights into the evolution of the suppressors of cytokine signalling (SOCS) gene**

**family in vertebrates**

**Bei Wang, Eakapol Wangkahart, Christopher J. Secombes and Tiehui Wang**

**Table of contents**

Fig. S1 The cDNA sequence and its translation of rainbow trout SOCS1b gene……………………..…..………..Page 3

Fig. S2 The cDNA sequence and its translation of rainbow trout SOCS2a2 gene………………..….…………….Page 4

Fig. S3 The cDNA sequence and its translation of rainbow trout SOCS3b1 gene……………………….….……..Page 5

Fig. S4 The cDNA sequence and its translation of rainbow trout SOCS3b2 gene…………………………….…...Page 6

Fig. S5 The cDNA sequence and its translation of rainbow trout SOCS4 gene…………………………..……......Page 7

Fig. S6 The cDNA sequence and its translation of rainbow trout SOCS5a1 gene…………………………..….….Page 8

Fig. S7 The cDNA sequence and its translation of rainbow trout SOCS5a2 gene………………………..………..Page 9

Fig. S8 The cDNA sequence and its translation of rainbow trout SOCS5b2 gene……………………..…………Page 10

Fig. S9 The cDNA sequence and its translation of rainbow trout SOCS6a2 gene………………………….….….Page 11

Fig. S10 The cDNA sequence and its translation of rainbow trout SOCS6b1 gene……………………..…….….Page 12

Fig. S11 The cDNA sequence and its translation of rainbow trout SOCS6b2 gene………………………..…….Page 13

Fig. S12 The cDNA sequence and its translation of rainbow trout SOCS7a2 gene……………………….….….Page 14

Fig. S13 The cDNA sequence and its translation of rainbow trout SOC7b1 gene…………………….….…..….Page 15

Fig. S14 The cDNA sequence and its translation of rainbow trout SOCS7b2 gene………………………..…….Page 16

Fig. S15 Analysis of a second SOCS4 locus in rainbow trout…………………………………………….…..….Page 17

Fig. S16 A maximum likelihood phylogenetic tree of vertebrate type II SOCS (SOCS1-3 and CISH)….......….Page 20

Fig. S17 A minimum evolotion phylogenetic tree of vertebrate type II SOCS (SOCS1-3 and CISH)….…....….Page 21

Fig. S18 A maximum likelihood phylogenetic tree of vertebrate type I SOCS (SOCS4-7) ………………....….Page 22

Fig. S19 A minimum evolotion phylogenetic tree of vertebrate type II SOCS (SOCS4-7). ………………....….Page 23

Fig. S20 The type II SOCS genes in vertebrates and the coincidence of gene loss/gain

in relation to 3R/4R WGDs………………………………………………………………....….Page 24

Fig. S21 The type I SOCS genes in vertebrates and the coincidence of gene loss/gain

in relation to 3R/4R WGDs…………………………………………………………………….Page 25

Fig. S22 CISH gene organisation across vertebrates…………………………………………………..…….…..Page 26

Fig. S23 SOCS1 gene organisation across vertebrates……………………………………………………….….Page 27

Fig. S24 SOCS2 gene organisation across vertebrates………………………………………………….....…….Page 28

Fig. S25 SOCS3 gene organisation across vertebrates…………………………………………………...…..….Page 29

Fig. S26 SOCS4 gene organisation across vertebrates………………………………………………….....…….Page 30

Fig. S27 SOCS5 gene organisation across vertebrates…………………………………………………….…….Page 31

Fig. S28 SOCS6 gene organisation across vertebrates…………….…………………………………..…..…….Page 32

Fig. S29 SOCS7 gene organisation across vertebrates……………………………………………….…....…….Page 33

Fig. S30 Comparative expression of trout SOCS paralogues ….……………………………………….……….Page 34

Table S1 Primers used for PCR cloning and real-time RT-PCR analysis of gene expression

of rainbow trout SOCS gene family members………………………………………….…..….Page 35

Table S2 The amino acid number and identity (top right)/similarity (bottom left) of

CISHa, CISHb and CISH…………………………………………………………..……..........Page 37

Table S3 The amino acid number and identity (top right)/similarity (bottom left) of

SOCS1a, SOCS1b and SOCS1…………………………………………………………......….Page 38

Table S4 The amino acid number and identity (top right)/similarity (bottom left) of

SOCS2a, SOCS2b and SOCS2………………………………………………………..…..…...Page 39

Table S5 The amino acid number and identity (top right)/similarity (bottom left) of

SOCS3a, SOCS3b and SOCS3………………………………………………………..………Page 40

Table S6 The amino acid number and identity (top right)/similarity (bottom left) of

SOCS4 homologues……………………………………………………………………...……Page 41

Table S7 The amino acid number and identity (top right)/similarity (bottom left) of

SOCS5a, SOCS5b and SOCS5…………………………………………………………….….Page 42

Table S8 The amino acid number and identity (top right)/similarity (bottom left) of

SOCS6a, SOCS6b and SOCS6…………………………………………………………….….Page 43

Table S9 The amino acid number and identity (top right)/similarity (bottom left) of

SOCS7a, SOCS7b and SOCS7…………………………………………………………….….Page 44

Table S10 Summary of intron/exon organisation of the vertebrate SOCS gene family…………………….….Page 45

Table S11 The p-value of a paired samples T test of the expression levels of SOCS paralogue pairs…………Page 46

Table S12 The Spearman's rho correlation coefficient (R) between the the

expression levels of SOCS gene family members…………………………………..……..….Page 47

**Fig. S1**

| 1 93 183 273 1 363 19 453 49 543 79 633 109 723 139 813 169 903 199 993 229 1083 259 1173 289 | ACGGTGACAGTAATGTAATGGTGTGATTTATAGCCGACGCATTGCACGCGGAGTATCTACATTCTTAGTGCGAAGTCTCCCACCACCGAGTC ATTTGGTTAACGTCTCATTCCTCTCCAGAA AACGGATCTCTCCAGATCATTCTGGATCTC TCTCTCCTCGCCCCTATAGTGGATTTGGAT ATGGGATAGGGATCCAGACTCCTGGACCAC GCAGGAGTATCTTCTAGTGCGACACACACA CACCGTCAGCTGGTTCACAGTGGCTACTAC TGGGGATGGGGGATCCAGTGGAGGGCTCTC TTCTGTATGATGGTCAGAGACACTCTGGCA GGAACCACAGTGGAACCATCGCAGAACCAC    M  M  V  R  D  T  L  A   G  T  T  V  E  P  S  Q  N  H  ACGACACCCACGCAGAACCAGGACGGAACC CAGCAGCTTCCAGGTGAAACCCTAGAACAC GTAGAACAACAGAACCGCGTTCAAGAACAA  T  T  P  T  Q  N  Q  D  G  T   Q  Q  L  P  G  E  T  L  E  H   V  E  Q  Q  N  R  V  Q  E  Q  CAGAACCAACTCGAGAACTTCGAACTCTTA GGTCAAGAGAATGAACTCTTAGAACAACAG AACCTAGAACATCAGATCCAACTCTTAGAA  Q  N  Q  L  E  N  F  E  L  L   G  Q  E  N  E  L  L  E  Q  Q   N  L  E  H  Q  I  Q  L  L  E  CTAGAGAATACGGTCCAGACCAGCACAGAC AACCGGACCACACCTCCAGACAAGACCAGT TCTGGCGGTGCCCTCCATCCCTGGTGGCCT  L  E  N  T  V  Q  T  S  T  D   N  R  T  T  P  P  D  K  T  S   S  G  G  A  L  H  P  W  W  P  CAACAGAGCAGCTTTAGAGCGACTAGACCC AGTGTCCCTCTCCCCTGGCTCGGACCTCTT CTCCACTTGCCCGATCCGATCCCAGACTCC  Q  Q  S  S  F  R  A  T  R  P   S  V  P  L  P  W  L  G  P  L   L  H  L  P  D  P  I  P  D  S  TGGACCACACACCTCCGGCCCTTCTTCAGC CAGCAGGAGTATCTTCTAGTGCGACGCACA CACCGTCAGCTGGTTCACAGTGGCTACTAC  W  T  T  H  L  R  P  F  F  S   Q  Q  E  Y  L  L  V  R  R  T   H  R  Q  L  V  H  S  G  Y  Y  TGGGGCCCTATGGAGATGGAGGAGGCCCAC CGCACACTGCTACTCACACCGCCTGGGAGC TTCCTCATCAGGGACAGCAGTCAGACGAAC  W  G  P  M  E  M  E  E  A  H   R  T  L  L  L  T  P  P  G  S   F  L  I  R  D  S  S  Q  T  N  GTCTTCTTCACCCTCAGTTACCACGGCGAC CGGGGGCCCGTCAGTGTCCGCGTCCTCCTG ACCAGTCAGAGTTTCCGCCTCAACGGCAGT  V  F  F  T  L  S  Y  H  G  D   R  G  P  V  S  V  R  V  L  L   T  S  Q  S  F  R  L  N  G  S  AACAAGGCCTTTGACTCATTGTTCGCCTTG CTGCGGTTCTACGTGGAGTCATCCCATAGG CGGCTGCGGAGGGCGTGGCGCCGGGAGCGA  N  K  A  F  D  S  L  F  A  L   L  R  F  Y  V  E  S  S  H  R   R  L  R  R  A  W  R  R  E  R  CCCATGACCTTACAGCAGCTGTGTAGGGGA CGAATCGTGGAGCTGTACGGCGCGGAGGGG ATAGATTCACTACCTGGGCTCAACCAGGTG  P  M  T  L  Q  Q  L  C  R  G   R  I  V  E  L  Y  G  A  E  G   I  D  S  L  P  G  L  N  Q  V  GTCACACAGTATCTACAGGACTATCCTTAT AGTATATAGACACACCCTCCTACCGGAGAG TTTGGGACACCACTTCCGTTTCAACAGACTG  V  [T](http://www.fr33.net/translator.php?modus=2&codon=AKA)  Q  Y  L  Q  D  Y  P  Y   S  I  *   |
| --- | --- |

**Fig. S1 The cDNA sequence and its translation of rainbow trout SOCS1b gene.** The primers used for cDNA cloning are boxed. The start and stop codons, and the immediate upstream in-frame stop codon of the main open reading frame (ORF) are in red. The arrows indicate intron positions.

**Fig. S2**

| 1 92 182 272 362 452 542 632 1  722 26 812 56 902 86 992 116 1082 156 1172 186 1262 1352  1442 | GGGTTTAATGGGGATTTGGTTAGTCAAAACACATACCGAAGGAAGGGGATTCAACGTCGCG GAAAAGACTGTCATTGGGATCTGTACCAAA AACAAGACAAGCGTGGTTTGGTGACTTTCT GCATGATATTTTAAATCTTAACATTACCAG CAGCAAGCGAGTCGTGCGGAGTCGACTTGC CTCGGTCGCGAGACGTTGTCATCGCTTGTT TTGCACCACGGGTTGGAAAAATATTCTTAG TTTGTTTTCCACCTCGCCAAAATGACAACT GATTGATTAGCCCGCCAGAAGATAAATAAT ACGACATGGAAAACTCTAGCTATGTAATCT GTCATCTTTCAATGTGATCGACATGGTTAC ACTGGCTAGCCGTTCCCTGGAAGTGTGTAG CTAACGAGCGGTTTTGTGCTTTGTTTATTT ATTCATATTAGTCCCCTTTTCTTTAGGGAG AAGTGTGTATTTGAATGGTCATCGGTTGAG TCGTTTGGTGCAGGATCCACTCGCAAATAG GACGATTCATTTATCAAATTCGAGAAGAAA CCTGCAGAAGAAACTGGTTTTGCGACTGTG GTGCAACTGCCTGTCTTTTGGGGAATATTT TTGCATTTGTCGGATGTCTTTTGGGCAATT TCGGGTTGTTCCCCAATGACCTGCCACTCA CCCGAATCCACCGAGACCATCGAAAATGAG AGGAGAACGGATACCGAGTCGCGAGTTGTA   M  T  C  H  S   P  E  S  T  E  T  I  E  N  E   R  R  T  D  T  E  S  R  V  V  GACTCCGATGAGACTCGCATCGGTCAAGCC ATGAAAGACCTTAAAAATACAGGCTGGTAC TGGGGCAGCCTGACCGCCAACGAAGCCAAA  D  S  D  E  T  R  I  G  Q  A   M  K  D  L  K  N  T  G  W  Y   W  G  S  L  T  A  N  E  A  K  GAGATTCTCCAGGATGCATCAGAGGGCACC TTCCTGGTGCGAGACAGCTCCCAGAGGGAC TACCTGTTCACCATCTCTGCCATGACCTCC  E  I  L  Q  D  A  S  E  G  T   F  L  V  R  D  S  S  Q  R  D   Y  L  F  T  I  S  A  M  T  S  GCCGGCCCAACTAACTTGCGCATCGAGTAC AAGGAGGGGAAGTTTAAACTAGACTCGGTG GTGCTGATCAAGCCCAAGCTCAAGCAGTTT  A  G  P  T  N  L  R  I  E  Y   K  E  G  K  F  K  L  D  S  V   V  L  I  K  P  K  L  K  Q  F  GACAGTGTGGTGCACCTGGTGGAACACTAC GTGCAGCTGTCCAGGACTACCAGTAAAGGT GCGTCGTCAGGGGCGTCGCAGTCCCTGGCC  D  S  V  V  H  L  V  E  H  Y   V  Q  L  S  R  T  T  S  K  G   A  S  S  G  A  S  Q  S  L  A  CCACCCAACGGCACGGTTCAGCTGCTACTG ACTAAGCCTGTGTACACTGCCACGCCCTCT CTACAGCACCTGTCCCGGATCGCCATCAAC  P  P  N  G  T  V  Q  L  L  L   T  K  P  V  Y  T  A  T  P  S   L  Q  H  L  S  R  I  A  I  N  AACGCCACCAGGCAGGTGCAGGAGCTGCCT TTACCCAACAGGCTAAAGAACTACCTGACG GACTACAGCTACAATGTATAGTAGGACTGC  N  A  T  R  Q  V  Q  E  L  P   L  P  N  R  L  K  N  Y  L  T   D  Y  S  Y  N  V  *    AAATCCCAGATGTGGTTTGGGATAGAGGGC AGTGCACCTGCCATGCAGTGGCAGTCAGAC TATCACCCAGTCCGAAGTCAGTCTTTAGCC CCAACCACTAGACACGGCTTGACGGCCCCC GTACTTCTGGAAGGATTGCTGTTGAGCAAT ATGCTTATAGAGCCAAGTCAGTCATCTAAT  CC |
| --- | --- |

**Fig. S2 The cDNA sequence and its translation of rainbow trout SOCS2a2 gene.** The primers used for cDNA cloning are boxed. The start and stop codons, and the immediate upstream in-frame stop codon of the main ORF are in red. The arrows indicate intron positions.

**Fig. S3**

| 1 91 1 181 15 271 45 361 75 451 105 541 135 631 185 721 215 811 245 901 991 1081 1171 1261 1351 | ACCTTCTTCTCTCTCTCCCTCCTGCCCCCT ACTCTCCCTCGCCCCTCCCTGCTCTGGACC CCCCACCCCTCGGTCAACGAGCCGAGCGTG GGGGTCAGGGCGCCATGGTAACCAACAGCA GGCGCGACCCCCCCCACCATGAGCAGCATG ACCCCCCCAGATCCCAGAGGTCAGGGGTCT     M  S  S  M   T  P  P  D  P  R  G  Q  G  S  CGTGTGCACCCCCTGCGCTATAAACCCTTC AGCTCACATGCACACTACCAACAGGTGCTG TGTGCGGTGCGTAAGCTACAAGAGAGTGGG  R  V  H  P  L  R  Y  K  P  F   S  S  H  A  H  Y  Q  Q  V  L   C  A  V  R  K  L  Q  E  S  G  TTCTACTGGGGTGCGGTGGGGGGTCGTGAG GCTAGCTCTCTGCTCCGCTCCCAGCCCCCC GGGACCTTCCTGGTTCGAGACTCATCCGAC  F  Y  W  G  A  V  G  G  R  E   A  S  S  L  L  R  S  Q  P  P   G  T  F  L  V  R  D  S  S  D  CACCACTACTTCTTCACACTGTCGGTCCAG ACGGCCCGCGGGACCAAGAACCTCCGTATC CACAGCCAGGGGGGAAGCTTTTACCTGCAA  H  H  Y  F  F  T  L  S  V  Q   T  A  R  G  T  K  N  L  R  I   H  S  Q  G  G  S  F  Y  L  Q  CCCGACTCGTGCTGCACACACATGCCCCCG CGCTTCGACTGTGTGCTCAAACTGATTGGA CACTACATGGGGAAGGAGGGGCGAGGTGGA  P  D  S  C  C  T  H  M  P  P   R  F  D  C  V  L  K  L  I  G   H  Y  M  G  K  E  G  R  G  G  GAAGCAGCAGGGGGAGGGGAGGCTGGGGTA AGGGAGGGGGGAGGCACGGCTGCGGCGGCA GGGGAGACTGTGGGGTCGGGGACTGGGAGT  E  A  A  G  G  G  E  A  G  V   R  E  G  G  G  T  A  A  A  A   G  E  T  V  G  S  G  T  G  S  GTGTATCTGATACACTCAGGGGGAGAGAAG GTTCCGTTGGAGCTGCGTCGACCCCTCCCC TCCTCCCTCTCCTCCCTCCAACACCTGTGT  V  Y  L  I  H  S  G  G  E  K   V  P  L  E  L  R  R  P  L  P   S  S  L  S  S  L  Q  H  L  C  AGGAGGACCCTGAACGGACACCTCGGGAGC TCTGCCAGCCCCGACACACACCAGCTCCCT CACACACTCAGGAACTTCTTGGAGGAATAC  R  R  T  L  N  G  H  L  G  S   S  A  S  P  D  T  H  Q  L  P   H  T  L  R  N  F  L  E  E  Y  GATGCCCCTATCTAACACACACATACACAC AAAAACACACACCCTCACACAGTTCAGGTT TGGGGTTTTCCCCTCTAACGGTTGAGGATA  D  A  P  I  *     GGATTTGGAAAGGGTAAGCTGTTCCAAATC GATGCCACTGCTTTCTTACCAAGGGGAGCC TAACATAGGTAACCATTGATACTCCTCCAC TCCTGGACAGACTGTTAATGGGAGTTAAGA TGAGACTGGGGGGAGGTTAAAAGTCGTTGC CCTCTCAGCCAGTGACCCAATCTCCCAGCG GGTGCTGTGATTGGGTAGTCAAAAAGATTG ATAGGTCGATAATGCAGACCGTGGACACGC CACACACAGTGACGTGTGTGGCTTTAGGAG TATAGCCAGAAGGTTGTTTGGGGTTTGTTG TGCTGCTGTGGCTAAGGTATAGCTCCTTCT ATTGATCAATAGAGGCTTTCATCTCCTCCC TCCCTTTTTTCTCCTGTTTGACATGTAGAA TGGGCTCAATGGACTGTGAGAGGGAGGAAA AGGAAGCGAGAGACAAGGAGAGAGGCGTAA TGAGAGTGTGTGGAGAACAGCAGTGTGTCA AACGAAGGGGCCAAAAGCAGAGTGAAAGTG TTTGTGGTGTCG |
| --- | --- |

**Fig. S3 The cDNA sequence and its translation of rainbow trout SOCS3b1 gene.** The primers used for cDNA cloning are boxed. The start and stop codons, and the immediate upstream in-frame stop codon of the main ORF are in red. The arrow indicates an intron position.

**Fig. S4**

| 1 93 1 183 16  273 46 363 76 453 106 543 136 633 166 723 196 813 226 903 993 1083 1173 1263 1353 | CCTCCATCACAACTCACCTCCTCCCCTTGTACCTCCTCCTCCCTCCCTGCTCTGGACCCCCCACCCCTCGGTCAGCAAGCCGAGCAGGGGGG GTCAGGGCGCCATGGTAACCCATAGCAGGC ACGACCCCCCCAGCCATGAGCAGCGTTACC CGCCCAGAGGCCAGAGGTCAGGGGTCAGAC     M  S  S  V  T   R  P  E  A  R  G  Q  G  S  D  GTGGACCCCCCGCGCTACAAACCCTTCAGC TCGCACGCACACTACCAACAGGTGCTGTGT GCGGTGCGTAAGTTACAGGAGAGTGGGTTC  V  D  P  P  R  Y  K  P  F  S   S  H  A  H  Y  Q  Q  V  L  C   A  V  R  K  L  Q  E  S  G  F  TACTGGGGTGTAGTGGGGGGTCGTGAGGCC AGCGCTCTGCTCCGCTCCCAGCCCCCCGGG ACCTTCCTGGTCCGAGACTCCTCCGACCAC  Y  W  G  V  V  G  G  R  E  A   S  A  L  L  R  S  Q  P  P  G   T  F  L  V  R  D  S  S  D  H  CACTACTTCTTCACACTGTCGGTCCAGACG GCTCGCGGGACCAAGAACCTCCGGATCCAC AGCCAGGGGGGAAGCTTTTACCTGCAGCCT  H  Y  F  F  T  L  S  V  Q  T   A  R  G  T  K  N  L  R  I  H   S  Q  G  G  S  F  Y  L  Q  P  GACCCCCACTGCACGCACACGCCCCCGCGT TTCGACTGTGTGCTCAAACTGATTGGACAC TACATGGGGAAGGAGGGAGGTGGAGGAGGA  D  P  H  C  T  H  T  P  P  R   F  D  C  V  L  K  L  I  G  H   Y  M  G  K  E  G  G  G  G  G  GATGGGGAGGCCGGGGCCGGGGTAAGGACA GGGGGAGGCATGACTGCGGCTGCAGGGGAG ACTGTGGGGACAGGGAGTTTGTATCTGATC  D  G  E  A  G  A  G  V  R  T   G  G  G  M  T  A  A  A  G  E   T  V  G  T  G  S  L  Y  L  I  CACTCAGGGGGAGAGAAGGTTCCGTTAGAA CTGCGTCGACCCCTCCCCTCCTCCCTGTCC TCCCTCCAGCACCTGTGTAGAAGGACCCTG  H  S  G  G  E  K  V  P  L  E   L  R  R  P  L  P  S  S  L  S   S  L  Q  H  L  C  R  R  T  L  AACGGACACCTGGGGGGCCCGGATCCCCCC GACACACACCAGCTCCCTCACACTCTAAGA GACTTCTTGGCGGAGTACGATGCTCCCATC  N  G  H  L  G  G  P  D  P  P   D  T  H  Q  L  P  H  T  L  R   D  F  L  A  E  Y  D  A  P  I  TAACACACACACACAGTTCATTTTGGGGGT TTCCCCATCTAACGGTTGAGGATAGGATTT GGAGAGGGTAAGCTGATCCTAGATCGATGC  *    TACTGCTTCCTATCAAGTGGAGCTTCATAT AGGTAGCCATGGATACGCCTCTCCTGGAGA GACTGTCAATGGGAGTCAGGAAAACGCTGG GGGTCAGAAGTGATTGCCCTGTCAGCCAGT TAGTCATTTTCCCAGCGGAAGCTGTGATTG GATAGTCAAAAAGATTACAGTGACGCAGAC CGTGAACGTGCTCACACGCAAGCAAACACA CACTTTAGCAGTGACTTTAGGAGTGTTGTC AGAAGGTTTCCTGTGGGTTTGTTGTGCTGC TGAGGAACAGCTCTTTCTATTGATTAACAG AAGCTTGCCTCTCCTCTTTCTCCTGTCTGA CATGTAAAATGGGCTGACTGGATTGAGAGA GAGAGAGGGAGATAGAGGGAGGGATGGAAG TATAATGAGAGAAAAGGAGAGAGGGGATGG GGAGTATGGCAGAGAATGTCAAATGAGGGG CCCATAGCAGAGTGAATGTGTTTGTGTGCA TGGTGTC |
| --- | --- |

**Fig. S4 The cDNA sequence and its translation of rainbow trout SOCS3b2 gene.** The primers used for cDNA cloning are boxed. The start and stop codons, and the immediate upstream in-frame stop codon of the main ORF are in red. The arrow indicates an intron position.

**Fig. S5**

| 1 1 92 6 182 36 272 66 362 96 452 126 542 156 632 186 722 216 812 246 902 276 992 306 1082 336 1172 366 1262 396 1352 1442 1532 1622 | CATGGACATGGAGATTACGTTGTCTCTCTAAAACTATTTTTAAAAGCACAGAGCTGTATGA AGAAGAGGGAAGGCCATGTCTGAGAAGAAA     M  S  E  K  K  TCCCGAAGTTCGGACATCTGTCCCAAATGC GGCATCCGCAGCTGGAGTGCCGATGGCTAC GTGTGGAGCTGCAAGAAACGCTCCCGGAGT  S  R  S  S  D  I  C  P  K  C   G  I  R  S  W  S  A  D  G  Y   V  W  S  C  K  K  R  S  R  S  TCTCGAAACGATCCGGGCCTTCGGCGTCCG GAGGGGGTAGGGCTGATGGAGGAGCAAGGA GCGCGTTCCACCTCATGTCCGCGGAGACGG  S  R  N  D  P  G  L  R  R  P   E  G  V  G  L  M  E  E  Q  G   A  R  S  T  S  C  P  R  R  R  AGAGAGAGGAAGTGTAGCTGTACCGTAATG GGGGAAGTTGACATAGATGTCCCCTGTCGG AAAGCCCTTTCTAGGCGCTCTCTCCGGCAG  R  E  R  K  C  S  C  T  V  M   G  E  V  D  I  D  V  P  C  R   K  A  L  S  R  R  S  L  R  Q  AAGTTCCAGGATGCAGTGGGTCAGTGTTTC CCTCTCCGCACTGACCATCGCCACCACCAC CACGGCTGCCCAACGGGGGCCTACCAGGGG  K  F  Q  D  A  V  G  Q  C  F   P  L  R  T  D  H  R  H  H  H   H  G  C  P  T  G  A  Y  Q  G  GCCTTTTCTGTGCTCCTCTGGTCCAAGCGT CCGATACATGTCACGGAGCTCATGCAGGAC AAGTGCCCCTTCTCGTCCAAGTCAGAGCTG  A  F  S  V  L  L  W  S  K  R   P  I  H  V  T  E  L  M  Q  D   K  C  P  F  S  S  K  S  E  L  GCCCACTGCTGGCACCTCATCAAGAAGCAT GCCACCCACCCCAGCGCCATTGTGGGCCTA GAGGTTGCCCAAGCCGCCAAGGCTGCACAG  A  H  C  W  H  L  I  K  K  H   A  T  H  P  S  A  I  V  G  L   E  V  A  Q  A  A  K  A  A  Q  GCTGCTGGCAAAGAACCAGTCCCGTCCACT TCCACATCCCCGCCTTCGACACCTCTTTCA TGGGAGGGCATCTGCTTGAGTAGGCCCCTG  A  A  G  K  E  P  V  P  S  T   S  T  S  P  P  S  T  P  L  S   W  E  G  I  C  L  S  R  P  L  AGCCTTGAGGACTGGGACCTCTCCCATCCG CATGGCAGAGCAGCCTATGGTGGCAGCCAT ACAGATTACATCCTAGTCCCTGACCTCCTG  S  L  E  D  W  D  L  S  H  P   H  G  R  A  A  Y  G  G  S  H   T  D  Y  I  L  V  P  D  L  L  CAGATCAACAACAGCTCGTGTTACTGGGGC GTTCTGGACCGCTTCCAGGCAGAGGAGCTC CTGGAAGGCCAGCCCGAGGGCACCTTCCTC  Q  I  N  N  S  S  C  Y  W  G   V  L  D  R  F  Q  A  E  E  L   L  E  G  Q  P  E  G  T  F  L  CTCCGTGACTCAGCCCAGGACAAGTTCCTC TTCTCCGTCAGCTTCCGCCGCTATAGCCGC TCCCTCCATGCGCGTATCGAGCAGAACGGT  L  R  D  S  A  Q  D  K  F  L   F  S  V  S  F  R  R  Y  S  R   S  L  H  A  R  I  E  Q  N  G  AAGCGCTTCAGCTTCGATGGCCGCGATCCG TGCATGTACCGGGATCCGAGTGTGACGGGC CTGCTCCGGCACTACAGCGACCCAGCCACA  K  R  F  S  F  D  G  R  D  P   C  M  Y  R  D  P  S  V  T  G   L  L  R  H  Y  S  D  P  A  T  TGCCTCTTCTTTGAGCCCCTCCTGTCCCGC CCTCTGGCCCGGACTTTCCCATTCACCCTG CAGCACCTGTGTCGCGCAGTGATCTGTAGC  C  L  F  F  E  P  L  L  S  R   P  L  A  R  T  F  P  F  T  L   Q  H  L  C  R  A  V  I  C  S  TGCACTACGTACCAGGGCATCAAGATCCTT CCACTGCCTCATCAGCTCAGGGACTATCTT AGGCAGTACCACTACAAGTGCAATGGGGCT  C  T  T  Y  Q  G  I  K  I  L   P  L  P  H  Q  L  R  D  Y  L   R  Q  Y  H  Y  K  C  N  G  A  TATGCAGTGTAAAACAATGATAATTTCTGA CTTCATAATTATAGTCCCATGGGTTACTAC TCTTCAATATTGACCATGTTTCATTGGTTT  Y  A  V  *     TTAGAGACTTGTGAAGACCCTGACTGCTAC AGTGCCTTCAGAAAGTATTCACACCCATAG ACTTTTTACACATTTTGTTGTGTTAAAATG GAGATTTTGTGTCACTGGCCTACACACAAT ATCAAGTAATGTCAAAGTGGAATTATGTTT TTAGAAATGTTTACAAATTAATTAAAAATT AAAAGCTGAAATGTCTTGAGTCAATAAGTA TTCAACCCCTGGCAAGCCTAAATAAGTTCA GGAGAAACAATTTGCTTAACAACTCACATA ATAAGTTGCACGGACTCACTGTGTGAGGTA ATAG |
| --- | --- |

**Fig. S5 The cDNA sequence and its translation of rainbow trout SOCS4 gene.** The primers used for cDNA cloning are boxed. The start and stop codons, and the immediate upstream in-frame stop codon of the main ORF are in red. The arrow indicates an intron position.

**Fig. S6**

| 1 92 1 182 28 272 58 362 88 452 118 542 148 632 178 722 208 812 238 902 268 992 298 1082 328 1172 358 1262 388 1352 418 1442 448 1532 478 1622 508 1712 538 1802 1892 | GGGATATGTGCCTGACTTGTCTTGATAGCCATTTTTTTGCAGACAAAGAGAACTGTTGAGT AGTGTTTATAAAAGACAAAAGGGTCTTGGT CTCCTATCCATGGAGAAAGTGGGCAAGATG TGGAGCAACCTGAGGAGCCGATGCCAGACC CTCTTCCACACCGACAGTGTGGGACCCAGT     M  E  K  V  G  K  M   W  S  N  L  R  S  R  C  Q  T   L  F  H  T  D  S  V  G  P  S  ACAGAGAACAGCGTGGTGGAGGTGGACGGT ATGCACTGTGTGGTGGACCTGGGACGGGGA GGCAATTCAGGCGAGGCCCAGGCTTCTCGG  T  E  N  S  V  V  E  V  D  G   M  H  C  V  V  D  L  G  R  G   G  N  S  G  E  A  Q  A  S  R  GCCTCTAGCCTGCCCCGAAGCCTCTTGCCG CTCCCCATGGTTACTGGGGGACGACGTCAC AACTGTGTGTCGGACATCCCCCAGATAGTG  A  S  S  L  P  R  S  L  L  P   L  P  M  V  T  G  G  R  R  H   N  C  V  S  D  I  P  Q  I  V  GAGATCACCATAGACAGCAAAGACAGTGAG GATGCGAGGGGGGGTCGTGGAGGAGTCCCT GTGGCCCGGAGAGACTCGTACTCACGCCAT  E  I  T  I  D  S  K  D  S  E   D  A  R  G  G  R  G  G  V  P   V  A  R  R  D  S  Y  S  R  H  GCACCTTGGGGGGGCAAGAAAAAACACTCA TGTTCCACTAAGACTCAGAGCTCCATGGAT ACAGACAGGTGGTCAGGGCGCGCGCGCGGG  A  P  W  G  G  K  K  K  H  S   C  S  T  K  T  Q  S  S  M  D   T  D  R  W  S  G  R  A  R  G  GCCGCTGGCCGGAGGGACCGTCGCTACGGG GTCAGCTCTATCCAGGAAATGGGGGACTCG GGGGGCGGGGGGCGCAGTCTGAGCGCCCGT  A  A  G  R  R  D  R  R  Y  G   V  S  S  I  Q  E  M  G  D  S   G  G  G  G  R  S  L  S  A  R  TCCCTGCGCCAGCGGCTTAGTGATACAGTG GGCCTGTGTCTCCCCCTGCCCCCCCGCCGC CGCTCGCGCTCTTCCAAGACCCCCACCGTC  S  L  R  Q  R  L  S  D  T  V   G  L  C  L  P  L  P  P  R  R   R  S  R  S  S  K  T  P  T  V  TTGAAGCGCAAGATCCACCTGACAGAGCTG ATGCTGGAGACCTGTCCCTTCCCCCAGGGC TCGGACTTGGCCAACAAGTGGCACCTGATC  L  K  R  K  I  H  L  T  E  L   M  L  E  T  C  P  F  P  Q  G   S  D  L  A  N  K  W  H  L  I  AAGCAGCACACGGCGCCCGTCAGCCCGCAT TCCTCCACGGCTCTGCTTGACGCCTTCGAC ACCGCCCACCCCTCCCCCGAGGACGAGGAG  K  Q  H  T  A  P  V  S  P  H   S  S  T  A  L  L  D  A  F  D   T  A  H  P  S  P  E  D  E  E  GAGCGTCTGCGCGAACGCCGCAGGCTCGGC ATTGAAGAGGGAGTGGACCCCCCACCCAAC GCCCAGATTCACACCCTGGAGGCCTTGGCG  E  R  L  R  E  R  R  R  L  G   I  E  E  G  V  D  P  P  P  N   A  Q  I  H  T  L  E  A  L  A  CAGGGCTCCTCTTTGTACAAACTGGGACCA AAGATGGCCCCCGGCATTGCAGAGGCCTCT GGGGAGGCCCGGGGCACCGCGGCCTGCTGC  Q  G  S  S  L  Y  K  L  G  P   K  M  A  P  G  I  A  E  A  S   G  E  A  R  G  T  A  A  C  C  TCAGGAGTGGGAGTATCGGTGCAGGTGCTC GGGGGGGCTACAGCCCAGTTGGCTGACTGT GACTCGGAGGAGGACTCAACTACCCTATGC  S  G  V  G  V  S  V  Q  V  L   G  G  A  T  A  Q  L  A  D  C   D  S  E  E  D  S  T  T  L  C  CTGCAGGCCCTGAGGCCCAAGCAGCGGCAC GCGTCCGGGGATGGCAATCTGAGCCGGAAC CAGCCTGGGCCATGGAAGGTGCACACGCAG  L  Q  A  L  R  P  K  Q  R  H   A  S  G  D  G  N  L  S  R  N   Q  P  G  P  W  K  V  H  T  Q  ATTGACTACATCCACTGCCTGGTACCGGAC CTGCTGCAGATCACTGCACTGCCCTGCTAC TGGGGCGTGATGGACCGCTACGAGGCGGAG  I  D  Y  I  H  C  L  V  P  D   L  L  Q  I  T  A  L  P  C  Y   W  G  V  M  D  R  Y  E  A  E  GCGCTGCTGGATGGACGGCCTGAGGGCACC TTCCTGCTGCGAGACTCGGCCCAGGAGGAC TACCTGTTCTCGGTCAGCTTTCGCCGCTAC  A  L  L  D  G  R  P  E  G  T   F  L  L  R  D  S  A  Q  E  D   Y  L  F  S  V  S  F  R  R  Y  AACCGCTCGCTGCATGCCCGCATCGAGCAG TGGAACCACAACTTCAGCTTCGATGCCCAC GACCCCTGCGTGTTCCACTCATCCACCGTC  N  R  S  L  H  A  R  I  E  Q   W  N  H  N  F  S  F  D  A  H   D  P  C  V  F  H  S  S  T  V  ACAGGCCTACTGGAGCACTACAAGGACCCC AGCGCCTGCATGTTCTTTGAGCCGCTGCTC ACGGCGCCACTCAACCGGGCCTTCCCTTTC  T  G  L  L  E  H  Y  K  D  P   S  A  C  M  F  F  E  P  L  L   T  A  P  L  N  R  A  F  P  F  GGCTTGCAGCACCTGGCACGCGCCGCCATC TGCCCCCGGACCACGTACGACGGCATCGGC GGCCTGCCACTGCCCCCGGCCCTGCAGGAC  G  L  Q  H  L  A  R  A  A  I   C  P  R  T  T  Y  D  G  I  G   G  L  P  L  P  P  A  L  Q  D  TTCCTCAAGGAGTATCACTACAAACAGAAA GTGCGTGTGCGCTGGCTGGAGAGGGAGCCG CCACTCAAGATCAAA**TAG**TGGCGGGGCTTT  F  L  K  E  Y  H  Y  K  Q  K   V  R  V  R  W  L  E  R  E  P   P  L  K  I  K  *    GGCGGCCTGTGCGTGCCGCCGCGGGCTTAG TCAAAACAGGAAAAACTCCCAGCCAATCAG GAGGTAATACTGAACATGGCACTTTTCAGT CAGGCAGGGA |
| --- | --- |

**Fig. S6 The cDNA sequence and its translation of rainbow trout SOCS5a1 gene.** The primers used for cDNA cloning are boxed. The start and stop codons, and the immediate upstream in-frame stop codon of the main ORF are in red. The arrow indicates an intron position.

**Fig. S7**

| 1 93 1 183 31 273 61 363 91 453 121 543 151 633 181 723 211 813 241 903 271 993 301 1083 331 1173 361 1263 391 1353 421 1443 451 1533 481 1623 541 1713 511 1803 1893 1983 2073 | GGGATATGTGCCTGACTTAAGCCATTTTTTTGCAGACAAAGAGAGCTACTGAGTACTGGTTTAAAAAGACAAAAGGGGCTTGGTCACTATCC ATGGAAAAAGTGGGCAAGATGTGGAGCAAC CTGAGGAACCGATGCCAGACCCTCTTCCAC AGCGACGGTGCGGGACCCAGTACAGAGAAC  M  E  K  V  G  K  M  W  S  N   L  R  N  R  C  Q  T  L  F  H   S  D  G  A  G  P  S  T  E  N  AGCGTGGTGGAAGAGGACGGTATGCACTGT GTGGCGGACCTGGGACGGGGAGAAACTTCA GGCGAGGCCCAGGCATCTCGGGCCTCCAGC  S  V  V  E  E  D  G  M  H  C   V  A  D  L  G  R  G  E  T  S   G  E  A  Q  A  S  R  A  S  S  CTTTCCCGAAGCCTCTTGCCGCTTCCCGGG GTTGCTGGAGGGCGACGTCACAACTGTGTG TCGGACATCCCCCAGATGGTAGAGAGAACC  L  S  R  S  L  L  P  L  P  G   V  A  G  G  R  R  H  N  C  V   S  D  I  P  Q  M  V  E  R  T  ACAGACAGAAAAGACAATGAGGAAGCGAGA GGGGCTCCTTGGGGAGTCCCTATGGGCCGG AGAGACTCGTACTCGCGCCATGCACCTTGG  T  D  R  K  D  N  E  E  A  R   G  A  P  W  G  V  P  M  G  R   R  D  S  Y  S  R  H  A  P  W  GGGGGCAAGAAAAAACACTCATGTTCCACT AAGACCCAGAGCTCCATGGATACAGACAGG CGGTCGGGACGCGCGCGCGGTGCCGCTGGG  G  G  K  K  K  H  S  C  S  T   K  T  Q  S  S  M  D  T  D  R   R  S  G  R  A  R  G  A  A  G  CGAAGGGAGCGTCGCTACGGGGTCAACTCC ATCCAGGAGATGGATGACTCTGGAGACGGG GGGCGTAGTCTGAGCACCCGCTCCCTGCGC  R  R  E  R  R  Y  G  V  N  S   I  Q  E  M  D  D  S  G  D  G   G  R  S  L  S  T  R  S  L  R  CAGCGGCTTAGCGACACAGTGGGCCTGTGT CTCCCCCTTCCCCCCCGCCGCCGCTCCCTC TCCTCCTCCCCCCAAACCCCCACCATCTCC  Q  R  L  S  D  T  V  G  L  C   L  P  L  P  P  R  R  R  S  L   S  S  S  P  Q  T  P  T  I  S  AAGCGCAAGATCCACCTGACAGAGCTGATG CTGGAGAACTGTCCCTTCCCCCAGGGCTCG GACCTGGCCAACAAGTGGCACCTGATCAAG  K  R  K  I  H  L  T  E  L  M   L  E  N  C  P  F  P  Q  G  S   D  L  A  N  K  W  H  L  I  K  CAGCACACAGCGCCTGTCAGCCCGCATTCC TCCTCGGCTCTGCTAGACGCCTTTGACCCG GCCCACCCCTCCCCCGAGGACGAGGAGGAG  Q  H  T  A  P  V  S  P  H  S   S  S  A  L  L  D  A  F  D  P   A  H  P  S  P  E  D  E  E  E  CGTCTGCGCGAACGCCGCAGGCTCAGCATT GAGGAGGGAGTGGACCCCCCACCCAACGCC CAGATCCACACCCTGGAGGCCTTGGCGCAG  R  L  R  E  R  R  R  L  S  I   E  E  G  V  D  P  P  P  N  A   Q  I  H  T  L  E  A  L  A  Q  TGCTCCTCTCTGTACAAACTGGGACCAAAG ATGGCCCCTGGCATTGCAGAGGCCTCTGGG GAGGGCACGATGGCCAGCTGCTCAGGAGGG  C  S  S  L  Y  K  L  G  P  K   M  A  P  G  I  A  E  A  S  G   E  G  T  M  A  S  C  S  G  G  GCGGCTGGGTCGTCGGGCTCGTCGGGACAG GTGCTCGGAGGGGCTACAGCCCAGCTGGCT GACTGCGACTCGGAGGAGGACTCCACCACC  A  A  G  S  S  G  S  S  G  Q   V  L  G  G  A  T  A  Q  L  A   D  C  D  S  E  E  D  S  T  T  CTTTGCCTGCAGGCCCTGAGGCCTAAGCTA CGCCACACGTCCGGGGATGGCCATCTGAGC CGGAACCAGCCTGGGCCCTGGAAGGTGCAC  L  C  L  Q  A  L  R  P  K  L   R  H  T  S  G  D  G  H  L  S   R  N  Q  P  G  P  W  K  V  H  ACCCAGATCGACTACATCCACTGCCTGGTG CCGGACCTGCTTCAGATCACTGCGCTGCCC TGCTACTGGGGCGTGATGGACCGCTACGAG  T  Q  I  D  Y  I  H  C  L  V   P  D  L  L  Q  I  T  A  L  P   C  Y  W  G  V  M  D  R  Y  E  GCAGAGGCTCTGCTGGATGGCCGGCCTGAG GGCACCTTCCTGTTGCGTGACTCGGCCCAG GAGGATTACCTGTTCTCGGTCAGCTTCCGT  A  E  A  L  L  D  G  R  P  E   G  T  F  L  L  R  D  S  A  Q   E  D  Y  L  F  S  V  S  F  R  CGCTACAACCGATCGCTGCATGCCCGCATC GAGCAGTGGAACCACAACTTCAGCTTCGAC GCCCACGACCCCTGCGTGTTCCACTCATCC  R  Y  N  R  S  L  H  A  R  I   E  Q  W  N  H  N  F  S  F  D   A  H  D  P  C  V  F  H  S  S  ACCGTCACGGGGCTACTGGAGCACTACAAG GACCCCAGCGCCTGCATGTTCTTTGAGCCG CTGCTCACGGCACCTCTCCACCGGACCTTC  T  V  T  G  L  L  E  H  Y  K   D  P  S  A  C  M  F  F  E  P   L  L  T  A  P  L  H  R  T  F  CCCTCCGGCTTGCAGCACCTGGCCCGCGCC GCCATCTGCCGCCGGACCACATACGACGGC ATCGGCGCCCTGCCGCTGCCCCCGGCCCTG  P  S  G  L  Q  H  L  A  R  A   A  I  C  R  R  T  T  Y  D  G   I  G  A  L  P  L  P  P  A  L  CAGGACTTCCTCAAGGAGTATCACTACAAA CAGAAAGTGCGTGTGCGCTGGCTGGAGAGG GAGCCGCCACTCAAGGTCAAATAGGGGAGC  Q  D  F  L  K  E  Y  H  Y  K   Q  K  V  R  V  R  W  L  E  R   E  P  P  L  K  V  K  *    GGTATCTGGCGGCCTGTGCGCCGCGGACGG AGTTGAAACAGAAAAATCCCTGCCAATCAG GAGAGGCCATACTGAATGAACAAAGCACTA CTCAGTCAGGCAAGGAGGGGTAACTGGAGA AACAAGGAGTTAGGTGAAGTGAGCTATCCA AGATTCATTCTAATCTCATTATTGTTTTAG  TTATCATTTACATCACTTGGGATACATATG TATCATTATATTCAGTACAATGCTGTCTGG CAAGCACACATGGTTTTTAATGTTGAACAG GTTCTGAAAGGGAGAGCACAAGCCTTCATG CTTTGTGTCCCTCTCACCTTTTTAATTCTG TCAAGTGTCTGCCTGATCCCACAGCATG |
| --- | --- |

**Fig. S7 The cDNA sequence and its translation of rainbow trout SOCS5a2 gene.** The primers designed for cDNA cloning are boxed. The start and stop codons, and the immediate upstream in-frame stop codon of the main ORF are in red. The arrow indicates an intron position.

**Fig. S8**

| 1 922 182 1 272 25 362 55 452 85 542 115 632 145 722 175 812 205 902 235 992 275 1082 305 1172 345 1262 375 1352 405 1442 435 1532 465 1622 495 1712 525 1802 555  1892 1982 2072 | AGCTTTTGGAGAGAACAACGAAGTTTTTATGCCCCAAGTGACTGTGACAGTGATTCATGGG ACATGCTATTGACAGCCTTTGGGAAGATTC TGAATGGGAGGGCTTGCTGCTGAGAACTGA CTACAGCCACAAATCACCTTTTCCCTGCAG CTGACCCCGACTGCAACTACCATTATTCCA GCCCATAATTAACCTGTCATGTCTGAACCA AAGGAGTCGGGTGATCGTGGGAAAGACAGG GAGCGGGGCGCCCGTCCCAAGGTGAGACAG   M  S  E  P   K  E  S  G  D  R  G  K  D  R   E  R  G  A  R  P  K  V  R  Q  AGCCGGTCTGAGGAGAGAAGAGATGCCGGC GGGGGGCAAAAGGGAGGAAGAGGAAAAAAG AAAGGCCAGACGTCCCATGAGCAAGCTGGG  S  R  S  E  E  R  R  D  A  G   G  G  Q  K  G  G  R  G  K  K   K  G  Q  T  S  H  E  Q  A  G  GAGCGGCCTGTCAGCGATGGGTTTGAGTAT GGGGACCTGCTGACTGGTCTGGAGCCCAGG GACCGCTGTTCCTCCTCTCCACTGAAGGAG  E  R  P  V  S  D  G  F  E  Y   G  D  L  L  T  G  L  E  P  R   D  R  C  S  S  S  P  L  K  E  GGCAGGAGATGGCAGGGCCTGGAGGGGGTC ACTTCACTCAGCCAGGACAGGGGGACAGCC AGGCTGGCACAGGGGACAGCTGAGCCACCA  G  R  R  W  Q  G  L  E  G  V   T  S  L  S  Q  D  R  G  T  A   R  L  A  Q  G  T  A  E  P  P  ACCAGTGAGGCTGAGGGCAGGGGGGCAGGT GGCAGTCGCACACTCCGCCAAAAGATCCAG GATGCCATGGGGCAGTGTTTCCCCATAAAG  T  S  E  A  E  G  R  G  A  G   G  S  R  T  L  R  Q  K  I  Q   D  A  M  G  Q  C  F  P  I  K  ACCAACACTCCGTCGTCCAGTTCCACTCAG CATGTCTTTATGCCACAAGCTGCTGCTGCC GGGGCTGGGTCCTCCTCGCGCCGCAAGATC  T  N  T  P  S  S  S  S  T  Q   H  V  F  M  P  Q  A  A  A  A   G  A  G  S  S  S  R  R  K  I  CACCTTACTGAACTCATGCTGGATGACTGT CCCTTCGCTGCAGGCACCGAGCTGGCTCAG AAGTGGTACCTCATCAAGCAGCACACAGCC  H  L  T  E  L  M  L  D  D  C   P  F  A  A  G  T  E  L  A  Q   K  W  Y  L  I  K  Q  H  T  A  CCCATCTCCACACCTCCCGTAGTGGACACC TTGGTGGTCAGCGCTAGTGCCTCTGCCTCA AACTTGGCCGCCGTGGTGGAGGATGTGGAT  P  I  S  T  P  P  V  V  D  T   L  V  V  S  A  S  A  S  A  S   N  L  A  A  V  V  E  D  V  D  GACCGGTTACGAGAGCGCAGGCGCATCAGC ATCGAGCAAGGCGTGGAGCCGCCACCCAAC GCAGAGATCCACACGTTTGAGGTGACGGCC  D  R  L  R  E  R  R  R  I  S   I  E  Q  G  V  E  P  P  P  N   A  E  I  H  T  F  E  V  T  A  CAGATCAACCCTCTGTACAAGCTGGGGCCC AAACTGGCCCATGGTATGAATGAGCTTGCA GGGGATGACAGAGCTACCATTCACCAGCAA  Q  I  N  P  L  Y  K  L  G  P   K  L  A  H  G  M  N  E  L  A   G  D  D  R  A  T  I  H  Q  Q  CAGCAGCTGCTTCTCCAGAGGCAACAGCAG CACCAGCTCTTGCTGCAGAGCTGTCTGGAC ACTCTCGATGAGGTGGTGGCCGTGGCCTCC  Q  Q  L  L  L  Q  R  Q  Q  Q   H  Q  L  L  L  Q  S  C  L  D   T  L  D  E  V  V  A  V  A  S  TCCTCTTCTGCCTCAGCATCTGCCTTGGTC CCTGTCTGTGAAGTTGCTTCTGTGCCTGAC CCCATGGTTGACCCTGAGGTCACAGCCAGC  S  S  S  A  S  A  S  A  L  V   P  V  C  E  V  A  S  V  P  D   P  M  V  D  P  E  V  T  A  S  CTCCAGCCAACCAAAATTGTTGTGCCCCAG GCTGAGGGTCCCCCTACTCAGGACGGCTAT CGCATCCACACCCAGATCGACTACATCCAC  L  Q  P  T  K  I  V  V  P  Q   A  E  G  P  P  T  Q  D  G  Y   R  I  H  T  Q  I  D  Y  I  H  TGTCTGGTGCCTGACCTGCTGCAGATCACT AACTTACCCTGCTACTGGGGTGTGATGGAC CGCTATGAGGCCGAGACACTGCTGGAGGGT  C  L  V  P  D  L  L  Q  I  T   N  L  P  C  Y  W  G  V  M  D   R  Y  E  A  E  T  L  L  E  G  AAGCCAGAGGGCACCTTCCTGCTCCGCGAC TCAGCCCAGGAAGACTACCTCTTCTCCGTC AGCTTCCGCCGCTACGGCCGCTCGCTGCAC  K  P  E  G  T  F  L  L  R  D   S  A  Q  E  D  Y  L  F  S  V   S  F  R  R  Y  G  R  S  L  H  GCCCGCATCGAGCAGTGGAACCACAACTTC AGCTTCGACGTGCACGACCCCAGTGTTTTC CATGCGCCCACCGTCACGGGGCTGCTGGAG  A  R  I  E  Q  W  N  H  N  F   S  F  D  V  H  D  P  S  V  F   H  A  P  T  V  T  G  L  L  E  CACTACAAGGACCCCAACTCCTGCATGTTC TTCGAGCCTCTGCTGTCCAACCCCATCCAC CGCACCCTGCCCTTCAGCCTGCAGCACGTG  H  Y  K  D  P  N  S  C  M  F   F  E  P  L  L  S  N  P  I  H   R  T  L  P  F  S  L  Q  H  V  TGCCGGGCGAGGATCAGCAGCTGCACCACC TACGACGGCATCAACGTGCTGCCCATCCCC AACACCCTGAAGAAACACCTGAAGGAGTAC  C  R  A  R  I  S  S  C  T  T   Y  D  G  I  N  V  L  P  I  P   N  T  L  K  K  H  L  K  E  Y  CATTACAAGCAGAGGGTGAGGGTACGGAGG ATGGACACCTGGTGGGAATAACAAAGACCT TCTGGTGGAAGTGAACCACAACTAGGCTAC  H Y K Q R V R V R R M D T W W E * TAACTTAACACTATGGAGGCAGGCCTCACT CAGTGGACAACCAGTTGACTCACTCCATTT TCTTCCATTTCGCTCTATTAACATATACCA  CCTACCTACTGCATTCACTGTATACCTAGC TACCTGTTTTATGTATTTATAATTTACAAT ATAAGACCAATGTCTTAAACTCTGTGGATA CTATGTTATTTCAATTCTGATGCACATTCA CCCATTTTTCTC |
| --- | --- |

**Fig. S8 The cDNA sequence and its translation of rainbow trout SOCS5b2 gene.** The primers used for cDNA cloning are boxed. The start and stop codons, and the immediate upstream in-frame stop codon of the main ORF are in red. The arrow indicates an intron position.

**Fig. S9**

| 1 92 182 1 272 2 362 32 452 62 542 92 632 122 722 152 812 182 902 212 992 242 1082 272 1172 302 1262 332 1352 362 1442 392 1532 422 1622 452 1712 482 1802 512 1892 1982 | CGTGTGGGATCTCTAAAGCCAGTTAGCTAGCTAGATGCCTGGCTAGCAGCCTGTCAGAAGC TAGCTAACGTCGTTAGGCAGTAACGTTAGC TGTCAAAACCAAGCCAAGTGCTTTGATTTC CCAGCGAACTCAAAATGCATCGATCTTGAC AGTTTACTGGGAAAAACGTTTTTAACAGAG GATGACAGGGATGACTGCTTTTGAAGGACA ACCTTTGTGGCCATCCACACATCTATGAGA TCCCCACATGTCTGAGGGACTTCCAAAATG   M  AAGAAAATTAGTCTGAAAACAATACGAAAG TCATTCAACCTAAAAGGCAAAGAGGAGGGA GAGGATGTTGTACCACAGCCAAATTCAGAT  K  K  I  S  L  K  T  I  R  K   S  F  N  L  K  G  K  E  E  G   E  D  V  V  P  Q  P  N  S  D  ACCAACTTTTCAACAGATTCAATGTTTGGA AAATGTTACAGCAAAGAACTTGTTTGTAAC GATTTTGATCACGAGGAGAAGAAAGGCCGT  T  N  F  S  T  D  S  M  F  G   K  C  Y  S  K  E  L  V  C  N   D  F  D  H  E  E  K  K  G  R  AAGAACGGCTCAAAAAGTGAGAGTCTTATG GGATCACTGAAAAGGAGGCTTTCGGCAAAA CAGAAAAGTAAAATAAAAGAAAGTTCCACA  K  N  G  S  K  S  E  S  L  M   G  S  L  K  R  R  L  S  A  K   Q  K  S  K  I  K  E  S  S  T  TCCGTAACCTGTGAAGATGACACATTCTCG TGCTCCTCAGCACCCATTTTCTTTAACGAC GTAAAATCACAGCACCATCTAAGATCTAGT  S  V  T  C  E  D  D  T  F  S   C  S  S  A  P  I  F  F  N  D   V  K  S  Q  H  H  L  R  S  S  AGTCACCATTATAGCCCCACGCCATGGGCC CTCAGGGCAGCGAATTCTGAGGAAACATGC CTTAGAATGGATGGGAAAGTGAAAGCTATG  S  H  H  Y  S  P  T  P  W  A   L  R  A  A  N  S  E  E  T  C   L  R  M  D  G  K  V  K  A  M  ATACACTCATCAGACCCCAGCCCATCTCTT TATGGCATTCAGAAAGTATTCCCTGACCCC CAGATGGATAGGCCTTTTCAGGATTCATCT  I  H  S  S  D  P  S  P  S  L   Y  G  I  Q  K  V  F  P  D  P   Q  M  D  R  P  F  Q  D  S  S  GAGAGCACTGAGCCTCAGAATTGTGATTTG CATCTAGATATTGATGTTGAAAATGTGCCT GCGATTATTGGACTATCACCTCAGAACTAT  E  S  T  E  P  Q  N  C  D  L   H  L  D  I  D  V  E  N  V  P   A  I  I  G  L  S  P  Q  N  Y  ATTCACTATGCAATGCCTTTAGATGATGAC CTTGAAAAAGGCTTGGATAATTCCTCTCCA ATAGATGAGGTGCCTCAGCCCGAGGGCTTC  I  H  Y  A  M  P  L  D  D  D   L  E  K  G  L  D  N  S  S  P   I  D  E  V  P  Q  P  E  G  F  CCTCCTCATGCAGCGGAAGACCCCATTGAC CAGGAGGAGCTGATGTCACCAGACATATTC ATGGACCCATCAGTGAATAGACTGCTCTAT  P  P  H  A  A  E  D  P  I  D   Q  E  E  L  M  S  P  D  I  F   M  D  P  S  V  N  R  L  L  Y  GAATCTGCAGGTGTCTTTATTCCAAACTCT AGAATAGATTCTCCTCTCTCCCCTTTGCTA CCTCAGCTACCTGGCAGTCACATCCGAAGG  E  S  A  G  V  F  I  P  N  S   R  I  D  S  P  L  S  P  L  L   P  Q  L  P  G  S  H  I  R  R  AGTTTCCAAGTATATGGTGGTTCTTCTCAT TCGCATGGTGCAGAGAGAGTAATGCACCAT CTCAACTTTGATCCCAATTCAGCCCCTGGC  S  F  Q  V  Y  G  G  S  S  H   S  H  G  A  E  R  V  M  H  H   L  N  F  D  P  N  S  A  P  G  GTTGGCAGGGTTTATGATGCTGTTCAGCAC AGCGGGCCCATGATTGTAACCAGCCTTACA GTAGAGCTAAAGAAACTGGCCAAGCAGGGT  V  G  R  V  Y  D  A  V  Q  H   S  G  P  M  I  V  T  S  L  T   V  E  L  K  K  L  A  K  Q  G  TGGTACTGGGGTCCTATAACGCGTTGGGAA GCTGAGGAAAAACTTGCTAGCCTACCAGAT GGGTCATTCTTGGTGCGAGACAGCTCCGAT  W  Y  W  G  P  I  T  R  W  E   A  E  E  K  L  A  S  L  P  D   G  S  F  L  V  R  D  S  S  D  GATCGTTATCTTTTGAGCTTGAGCTTTCGG TCACAGGGTAAGACCCTCCACACCAGGATC GAACACTCCAATGGCAGTTTCAGTTTCTAC  D  R  Y  L  L  S  L  S  F  R   S  Q  G  K  T  L  H  T  R  I   E  H  S  N  G  S  F  S  F  Y  GAACAGCCAGATGTGGAGGGTCACATATCA ATAGTGGAACTCATTGAACATTCCATCAGA GATTCTGAGAGTGGAGCCTTCTGCTATTCA  E  Q  P  D  V  E  G  H  I  S   I  V  E  L  I  E  H  S  I  R   D  S  E  S  G  A  F  C  Y  S  CGATCTCGTACACCAGGGACTGCCACGTAT CCTGTCAGACTGACAAACCCTATCTCAAGG TTTATGCAGGTGCGTTCCATGCAGTACCTC  R  S  R  T  P  G  T  A  T  Y   P  V  R  L  T  N  P  I  S  R   F  M  Q  V  R  S  M  Q  Y  L  TGTCGGTTTGTCATCCGTCAGTACACACGG ATTGACCTTATCCAGAAATTGCCTTTGCCA AACAAGATGAAAGATTACTTGCAGGAAAAG  C  R  F  V  I  R  Q  Y  T  R   I  D  L  I  Q  K  L  P  L  P   N  K  M  K  D  Y  L  Q  E  K  CACTACTGAAGAAGATCAATGAAACCCAGT TGGACTTTCTTGCACATTTTAGATCAGCAA CATAATGAAATAGGCCTAGTTTTTAATCTT  H  Y  *    TTGGACATGACCCTAATCCAGTGATGTGTC CACCAGCTAATGCCAGTAACTGACATGTCT TGTGAGCCATACAGTTATACTCCCCACCTC AAAGCCAAGGATTGAAG |
| --- | --- |

**Fig. S9 The cDNA sequence and its translation of rainbow trout SOCS6a2 gene.** The primers used for cDNA cloning are boxed. The start and stop codons, and the immediate upstream in-frame stop codon of the main ORF are in red. The arrow indicates an intron position.

**Fig. S10**

| 1 93 183 273 363 453 1 543 12 633 42 723 72 813 102 903 132 993 162 1083 192 1173 222 1263 252 1353 282 1443 312 1533 342 1623 372 1713 402 1803 432 1893 462 1983 492 2073 512 2163 2251 | GGAGAGAAGAAGAGGGCGACAGAAAGTATTTAAATAAATAGATTGTTGTTGAAGTACTACAATACAACATAAATAGCCAAAGCTAAAACGGG GAACGTTTGCAACACACTTTTGTTTTATTT TTATAGCTAGCGAGATACAGTTGCTTCCGC GAAATGTGTTTGACTGCTAAGCTAGCCGAC TGGCTAAACTAAGTAGCTAGCAAGTTAGCT CAGCCCGGTTCCGGCCTAAAATAGCTAACG TTAGCTCCATTTTCAGATTTCGGGAAAAAA CCCAATTCAAGTGTACATTACCACGTATGC AGATGTTCATCTAACGAAAGCAAAGGGGAA GCTGATGGAACGTTAGTCCAACTGAGTCAC CAATTCTAGTCATGCAATGGGGATGAGCGC TCTGCAGTGTGTATGACCAGCTGTCTCCAG CCTGATGGGAGGACTCTGTGGATAGCAGGA TAGTCTTCAGGACTCCAGGAGGAGAGTAGC CGCCCCTGCCAAGGAGGGCCGTTCAGAATG AAGAAAATCAGCCTTAACACCATCCGCAAG       M   K  K  I  S  L  N  T  I  R  K  TCCCTCAACATCAAGGTCAAGGAGGAAGGG GGGGGGGACTTTGTCATGCTCCAGCAGCCC TCGCTAGCGGCCGACTTCTCCAAGGAGGAA  S  L  N  I  K  V  K  E  E  G   G  G  D  F  V  M  L  Q  Q  P   S  L  A  A  D  F  S  K  E  E  TCGCTCTTCGGGGGCTGCTACACTAAAGAT CTTGCGGGCTGCAACCTGGGAATTGGTGAT GTGGGAGAGGAGAAGGCGGGACACAACAAG  S  L  F  G  G  C  Y  T  K  D   L  A  G  C  N  L  G  I  G  D   V  G  E  E  K  A  G  H  N  K  GGCCGGTCTAAGAGCGAGAGCCTGATGGGT TCGCTGAAGAGGAGGCTGTCGGTCAAGCAG AAGGCCAAGGTCAAAGGTGGCTCATTAGCC  G  R  S  K  S  E  S  L  M  G   S  L  K  R  R  L  S  V  K  Q   K  A  K  V  K  G  G  S  L  A  ATGGGGTCGGCTGACGATGATGACACCTTC TCCTCCTCCTCGGTCCCAATCAGCTTCAAC GAGGTCAAGGCCCAGCGTCCGTTACGATCA  M  G  S  A  D  D  D  D  T  F   S  S  S  S  V  P  I  S  F  N   E  V  K  A  Q  R  P  L  R  S  GCGTCGCTCCGTAGCCACCATTACAGCCCG TCTCCCTGGCCCCTGCGGCCTGTCGCTTCA GATGAGGCCTGCATCAAAATGGAGGTGAAG  A  S  L  R  S  H  H  Y  S  P   S  P  W  P  L  R  P  V  A  S   D  E  A  C  I  K  M  E  V  K  GTTAAAGCCATGGTCCATTCCCCCAGCCCA GACCTGAACGGCGTGCGGAAGGAGTTCCGC CACCATGACTTTCAGATGGAGAACATCTTC  V  K  A  M  V  H  S  P  S  P   D  L  N  G  V  R  K  E  F  R   H  H  D  F  Q  M  E  N  I  F  CAGGAGCCGCAACACGGAGACCTGAACAGT GACAAACACGTGCCTGTAGTCCTGGGCCTC ACGCCACAGGACTACATTCAGTACACCATG  Q  E  P  Q  H  G  D  L  N  S   D  K  H  V  P  V  V  L  G  L   T  P  Q  D  Y  I  Q  Y  T  M  CCTTTAGAGGAGGGGATGTACCCAGAGGGG TCCCACTCTGTCCCCCACTCCTTCTGCCTG GACAGGTCACTGCCTATGGAGGTGGTGACG  P  L  E  E  G  M  Y  P  E  G   S  H  S  V  P  H  S  F  C  L   D  R  S  L  P  M  E  V  V  T  GAGGCGGACAGTAGCTCCCTCCGCGTCGAC CACCAGAGTCAGGAGGACCAGGATCTGGTG AGTCTGAACCGGAACCTTCCAGCGGACCTC  E  A  D  S  S  S  L  R  V  D   H  Q  S  Q  E  D  Q  D  L  V   S  L  N  R  N  L  P  A  D  L  TCCGTGGAGTCACAGTCGGTGAACGGCCTC TTCATCGGCTCTAACGGTGTGATGCTCCAT AGCTTCAGGGACAGGGTCAACGCTCAACAA  S  V  E  S  Q  S  V  N  G  L   F  I  G  S  N  G  V  M  L  H   S  F  R  D  R  V  N  A  Q  Q  CACCCCGCTCCTCCCCAGCGCTCCCCTCTC CTGCCCGCGTTACCCAGCAACAACATCTCC AGGACTTACTCCAGGTTCGGCGGGGCAGAT  H  P  A  P  P  Q  R  S  P  L   L  P  A  L  P  S  N  N  I  S   R  T  Y  S  R  F  G  G  A  D  GGCCACGTGGCGGCCCGGGTGAGGCAACAC CTGAACTTTGACCTCGACTCTGCCCCAGGG GTGAGTCGGCTGTATGACTCAGTCCGGAGC  G  H  V  A  A  R  V  R  Q  H   L  N  F  D  L  D  S  A  P  G   V  S  R  L  Y  D  S  V  R  S  AGTGGACCAATGGTAGTAACCAGCCTGACT GAGGAGCTGAAGAAGCTAGCCAGGCAGGGT TGGTACTGGGGACCTATCACACGCTGGGAG  S  G  P  M  V  V  T  S  L  T   E  E  L  K  K  L  A  R  Q  G   W  Y  W  G  P  I  T  R  W  E  GCTGAGGAGAAGCTGGTCAACCTGCCTGAC GGCTCGTTTCTGGTCAGAGACAGTTCGGAC GACAGGTACCTCCTCAGCCTTAGTTTCCGC  A  E  E  K  L  V  N  L  P  D   G  S  F  L  V  R  D  S  S  D   D  R  Y  L  L  S  L  S  F  R  TCCCAGACCAAGACCCTCCACACTCGCATC GAACACTCCAACGGACGCTTCAGCTTCTAC GAGCAGCCCGACGTGGAGGGACACACGTCC  S  Q  T  K  T  L  H  T  R  I   E  H  S  N  G  R  F  S  F  Y   E  Q  P  D  V  E  G  H  T  S  ATGGTGGACCTGATAGAATTCTCCGTCAAA GACTCTGAGAACGGAGCCTTCTGTTATTCT AGATCTCGCTTACCAGGGTCCGCCACCTAC  M  V  D  L  I  E  F  S  V  K   D  S  E  N  G  A  F  C  Y  S   R  S  R  L  P  G  S  A  T  Y  CCCGTCAGGCTGACCAATCCCGTGTCTCGG TTTATGCACGTGCGCTCTCTGCAGTACCTG TGTCGGTTTGTGATTAGACAGTACACCAGG  P  V  R  L  T  N  P  V  S  R   F  M  H  V  R  S  L  Q  Y  L   C  R  F  V  I  R  Q  Y  T  R  ATTGACCTAATTCAGAACCTGCCTTTGCCC AACAAGATGAAAGACTACCTGCAGGAGAAG CACTAC**TGA**CAGGGTTGACACTGACAGATG  I  D  L  I  Q  N  L  P  L  P   N  K  M  K  D  Y  L  Q  E  K   H  Y  *   GGACAGACAACGGTAGCAACTTGTACTTTT GGGTTCAGTTGAAGAGATTCAACAAAGGTT TACAGACATCCGTAGTTTGGAGACGATCGA  TTGGGTTCCATTGGCTCTTTGTTTGTGTCC AGGGATGTCTGTATGTAGTG |
| --- | --- |

**Fig. S10 The cDNA sequence and its translation of rainbow trout SOCS6b1 gene.** The primers used for cDNA cloning are boxed. The start and stop codons, and the immediate upstream in-frame stop codon of the main ORF are in red. The arrows indicate intron positions.

**Fig. S11**

| 1 92 182 272 1 362 9 452 39 542 69 632 99 722 129 812 159 902 189 992 219 1082 249 1172 279 1262 309 1352 339 1442 369 1532 399 1622 429 1712 459 1802 489 1892 519 1982 549 2072 579 2162 609 2252 | GCAAGAGAGAGAGGAAGAGTCCGTCAGAAAGTATTAGAAAAGACAGAAAAAAATAGATTGT TGTTGAAAAATTACAATACAGCATAATAAA CAGCCATAGATAAAACGGGGAACCATTCCA ACGCACTTTTATTTTGTTTTGCTAGCTAGA TACGGTAGCTCCTTCCACGATATGCTTGTG ACTGCTAAGCTAGCTTGCTGACTGGCTAAG CTAAGTAGCTAGCTCAGTTCTATCTTCTAA AATACATTTTCAAATTTAGGTAAAACCCAA TTCAAGTGTACATTACCACGAATGCAGATG GTAATCTAACGAAGGGATGAGTGCTGTGCA GTGTATATGACCAGCTGTCTCCAGCCTGAT    M  T  S  C  L  Q  P  D  GGGAGGACTCTGTGGATAGCAGCATCTTCA TCAGGACCCCAGGAGGAGTGCAGCCATCCC GTCCCAGTCTCATGTCCCACACCCCTTCCC  G  R  T  L  W  I  A  A  S  S   S  G  P  Q  E  E  C  S  H  P   V  P  V  S  C  P  T  P  L  P  TGCCAAGGAGGGCCGTTCAGAATGAAGAAA ATCAGCCTTAAGACCATCTGCAAGTCCCTC AACATCAACAAGGGCAAGGAAGAAGGAGGG  C  Q  G  G  P  F  R  M  K  K   I  S  L  K  T  I  C  K  S  L   N  I  N  K  G  K  E  E  G  G  GGGGATTTTGTCATGCTCCAGCAGCCCTCG CCAGCGGTCGACTTCTCCAAGGAAGACTCT CTTTTCAGGGGCTGCTACACCAAAGAGCTG  G  D  F  V  M  L  Q  Q  P  S   P  A  V  D  F  S  K  E  D  S   L  F  R  G  C  Y  T  K  E  L  GCGGGCTGCGACCTTGGAGGTGGCGGTGGA GGAGTTGAAGAAGAGGAGAAGGCGGGACAC AACAAGGGCCGTTCTAAGAGCGAGAGTCTG  A  G  C  D  L  G  G  G  G  G   G  V  E  E  E  E  K  A  G  H   N  K  G  R  S  K  S  E  S  L  ATGGGTTCTCTGAAGAGGAGGCTGTCGGCC AAGCAGAAGGCCAAAGTCAAAGGGGGCTCC ACAGCAATGGGGTCAGTTGACGATGACGAT  M  G  S  L  K  R  R  L  S  A   K  Q  K  A  K  V  K  G  G  S   T  A  M  G  S  V  D  D  D  D  ACCTTCTCCTCCTCCTCTGTGCCCATCAGC TTCAACGAGGTCAAGGCCCATCGGCCGTTA CGATCGGCATCCCTCCGCAGCCACCATTAC  T  F  S  S  S  S  V  P  I  S   F  N  E  V  K  A  H  R  P  L   R  S  A  S  L  R  S  H  H  Y  AGCCCCTCTCCCTGGCCCCTGCGGCCTGTG AACTCAGACGAAGCTTGCATCAAGATGGAG GTGAAAGTTAAAGCCATGGTCCACTCTCCG  S  P  S  P  W  P  L  R  P  V   N  S  D  E  A  C  I  K  M  E   V  K  V  K  A  M  V  H  S  P  AGCCCCAGCCCCAACCTGAATGGTGTTCGG AAGGATTTCCACCACCAGGACTTCCAGATG GAGGGCATCTTTCAGGAGCAGGCCGTGAAG  S  P  S  P  N  L  N  G  V  R   K  D  F  H  H  Q  D  F  Q  M   E  G  I  F  Q  E  Q  A  V  K  AACCTCCAGGAGCAGGCAGAGTCCCTGAAG AACCTCCAGCAGCCCCAAAACGGAGACCAG CTGCACCTGAACATTGATGAACACGTGCCT  N  L  Q  E  Q  A  E  S  L  K   N  L  Q  Q  P  Q  N  G  D  Q   L  H  L  N  I  D  E  H  V  P  GTAGTCTTGGGCTTCACGCCACAGGAATAC ATCCAGTACACCATACCTTTAGAGGAGGGA ATGTACCCAGAGGGGTCCCACTCTGTCTCT  V  V  L  G  F  T  P  Q  E  Y   I  Q  Y  T  I  P  L  E  E  G   M  Y  P  E  G  S  H  S  V  S  CACTCCTTCTGCCTGGACGGGTCCCTGCCT ATGGAGGTGGTGACTGAGGCAGACAGTAGC TCTCTCCACGCTGATCAGCAGAGTCAGGAA  H  S  F  C  L  D  G  S  L  P   M  E  V  V  T  E  A  D  S  S   S  L  H  A  D  Q  Q  S  Q  E  GACCAGGATCTGGTGAGTATGAACCTGAAC CTGCCAGTGGACCTCTTCATGGAGTCTGTC AACGGCCTCCTCATCGGCTCTAACGGCGTG  D  Q  D  L  V  S  M  N  L  N   L  P  V  D  L  F  M  E  S  V   N  G  L  L  I  G  S  N  G  V  ATGCTCCAGAGCTCCAGGGACAGGGTCGAT GCCCAACACCCCCCTCCTCCCCCCCTGCCC CTGCTACCCAGCAACGAGATCCCTAGGACT  M  L  Q  S  S  R  D  R  V  D   A  Q  H  P  P  P  P  P  L  P   L  L  P  S  N  E  I  P  R  T  TGCTCTGGGTTCGGTGTTGCGTACAGCCAC GTGGCGGAGCGGGTGAGGCACCACTTAAAC TTTGACCCCAACTCGGCTCCGGGCATGAGC  C  S  G  F  G  V  A  Y  S  H   V  A  E  R  V  R  H  H  L  N   F  D  P  N  S  A  P  G  M  S  CTGGTGTATGACTCAGTCCAGAGCAGCGGA CCCATGGTTGTGACCAGCCTGACCGAGGAG CTGAAGAAGCTAGCCAGGCAGGGTTGGTAC  L  V  Y  D  S  V  Q  S  S  G   P  M  V  V  T  S  L  T  E  E   L  K  K  L  A  R  Q  G  W  Y  TGGGGGCCCATCACACGCTGGGAGGCTGAG GAGAAGCTGGTCAACCTGCCCGACGGCTCG TTCCTGGTCAGGGACAGCTCGGACGACAGG  W  G  P  I  T  R  W  E  A  E   E  K  L  V  N  L  P  D  G  S   F  L  V  R  D  S  S  D  D  R  TACCTCCTCAGCCTTAGTTTCCGCTCCCAG ACCAAGACCCTCCACACTCGCATCGAACAC TCCAACGGACGCTTCAGCTTCTACGAGCAG  Y  L  L  S  L  S  F  R  S  Q   T  K  T  L  H  T  R  I  E  H   S  N  G  R  F  S  F  Y  E  Q  CCCGACGTGGAGGGACACACGTCCATCGTA GACCTGATAGAATACTCCATCAAAGATTCG GAGAATGGAGCCTTCTGTTATTCTAGATCT  P  D  V  E  G  H  T  S  I  V   D  L  I  E  Y  S  I  K  D  S   E  N  G  A  F  C  Y  S  R  S  CGCTTACCAGGGTCCGCCACCTACCCCGTC AGGCTGACCAATCCCGTGTCTCGTTTTATG CAAGTGCGCTCCCTGCAGTACCTGTGTCGG  R  L  P  G  S  A  T  Y  P  V   R  L  T  N  P  V  S  R  F  M   Q  V  R  S  L  Q  Y  L  C  R  TTCGTGATCAGACAGTACACCAGGATTGAC CTGATTCAGAACCTGCCTTTGCCCAACAAG ATGAAAGACTACCTGCAGGAGAAGCACTAC  F  V  I  R  Q  Y  T  R  I  D   L  I  Q  N  L  P  L  P  N  K   M  K  D  Y  L  Q  E  K  H  Y  **TGA**CAGGGTTGACACTGACAGATAGGACAG ACAATGGTAGCAACTTGCACTTTTGGGTTC AGTTTAAGAGATTTAACAAAGGTTTACAGA  *   CATCCATCGTTTAGACAAGATCGATTGGGT TCCATTGGCTCTTTGTTTTTTCCCAGGGAC GTGTCCAAAATGGAACCCTATTCCG |
| --- | --- |

**Fig. S11 The cDNA sequence and its translation of rainbow trout SOCS6b2 gene.** The primers used for cDNA cloning are boxed. The start and stop codons, and the immediate upstream in-frame stop codon of the main ORF are in red. The arrow indicates an intron position.

**Fig. S12**

| 1 91 1 181 31 271 61 361 91 451 121 541 151 631 181 721 211 811 241 901 271 991 301 1081 331 1171 361 1261 391 1351 421 1441 451 1531 481 1621 511 1711 541 1801 571 1891 601 1981 631 2071 661 2161 691 2251 | GTTTCTGATGCGCCTCGACCTCCCGACTTC GCCGTGGCTCAGCGCTTCGATTTCCCGCAT GGCGCGGGCAGAGGCTCGCGGGCTCAGCTC ATGGTGTTTCAGAGCGTCCTTAGGACAAGC GACGGTATTTTGGAATGCGGCCTCGAGCAA CCTCCTGGCTTCCAGGTAAGTGAGGCTGAT  M  V  F  Q  S  V  L  R  T  S   D  G  I  L  E  C  G  L  E  Q   P  P  G  F  Q  V  S  E  A  D  AAACAGGAAGCCAGTTCTGCCTGTGTCATG ATGACCGGTGACAACAATATGGATGTGCAA CACCAGAGGCTTCAGTGGCACCCCATTATG  K  Q  E  A  S  S  A  C  V  M   M  T  G  D  N  N  M  D  V  Q   H  Q  R  L  Q  W  H  P  I  M  AAGCTGTCCAAGGTGGTTGCAGATGCAGGT GACCTGGCTGGAGAGGGAGATGGGCTCTGC CACCGGCACCGCCTGGTCACTGATGCCATG  K  L  S  K  V  V  A  D  A  G   D  L  A  G  E  G  D  G  L  C   H  R  H  R  L  V  T  D  A  M  GACTGGCCTCCGCTCCTGGACAAGTCCCTC CGTTTCTGCATCCTGGACCCCAAGAAGACC TGTTCGACAGGGGACACGATCTACCACCAC  D  W  P  P  L  L  D  K  S  L   R  F  C  I  L  D  P  K  K  T   C  S  T  G  D  T  I  Y  H  H  CTGGACGACACGGTGCTCAACCTGGCCCGG AGGCTGGGGGAGCTGGGCCAGGCCTCAGAG ATGCTGCTGAAGGAGGGTGGAGAGATGCCT  L  D  D  T  V  L  N  L  A  R   R  L  G  E  L  G  Q  A  S  E   M  L  L  K  E  G  G  E  M  P  CGCTGTTCCTGTCAAAGCATCCTGGCTTCG GCAACGGGGGGCATAGGCCCCGGAGAGGAC CCTAGTGAAACCAGTGACGCCCTCCTGGTG  R  C  S  C  Q  S  I  L  A  S   A  T  G  G  I  G  P  G  E  D   P  S  E  T  S  D  A  L  L  V  CTGGAGGGCCTGGACTCTGAGGAAGTGGGT GAGCTGGGGCTGGGCGGGGAGGAGTTTAAA GGTGGTGTCCCTGGGCAGGAGGGCGAGACA  L  E  G  L  D  S  E  E  V  G   E  L  G  L  G  G  E  E  F  K   G  G  V  P  G  Q  E  G  E  T  GACATGGGGCAGGACCTAATGAGGCAGGTA CACAGTCTTGCTGGGGAGTTCTGTGCCTGT GACCCCCAGGTGTGTCCCTCCCCGTTGGAT  D  M  G  Q  D  L  M  R  Q  V   H  S  L  A  G  E  F  C  A  C   D  P  Q  V  C  P  S  P  L  D  GCCCTGGGCTCAGCTGCAGCCTTAACATCG TCCCTGTCTCTGGCCTCCTCTAACGTCACG GGACAGTCGGCAGCAGAAGCTTCCGCCACC  A  L  G  S  A  A  A  L  T  S   S  L  S  L  A  S  S  N  V  T   G  Q  S  A  A  E  A  S  A  T  CTATCTGGCACCTTACCTTCACAGGACCCT CCCAGCCAAACCTCCCAGCCCCAAAGCAGG GCCACCACGCCAAAGTTAGGGGTGATGTTG  L  S  G  T  L  P  S  Q  D  P   P  S  Q  T  S  Q  P  Q  S  R   A  T  T  P  K  L  G  V  M  L  CGAGCTGCCTCACCTGTTGTAGTAGAGGGG GCGGCAGGGGGCGAGAAGACGACCAGAGGG AAGTCCCGGAAAGGCTCTCTAAAGATCCGT  R  A  A  S  P  V  V  V  E  G   A  A  G  G  E  K  T  T  R  G   K  S  R  K  G  S  L  K  I  R  CTGAGCAAACTATTCCGGACCAAGAGCAGC AGCGGCTCCAGTCACCTACTGGACAAGAGA CCCTCGCTGGCCTCCTCCACCTCCTCCGGG  L  S  K  L  F  R  T  K  S  S   S  G  S  S  H  L  L  D  K  R   P  S  L  A  S  S  T  S  S  G  GGCAGCCTGATGGATGTGTGGGGCTCTGGA TCCACCAGCACAGACTTGGACACAGGGAGC AAACACCAGCTCCCCAGGCCCCAAAGTGCC  G  S  L  M  D  V  W  G  S  G   S  T  S  T  D  L  D  T  G  S   K  H  Q  L  P  R  P  Q  S  A  TTCTCTCCGTTGGCCTTCGGTCCTGCCTTC ACCGATGAGACTGTGTCCCTTGTAGACGTG GATATTTCTCGTAGAGGGGTGAACTCTCTG  F  S  P  L  A  F  G  P  A  F   T  D  E  T  V  S  L  V  D  V   D  I  S  R  R  G  V  N  S  L  CAACCCCCGACACCTCCACCTCCGCCCAGG CGAAGCCTCAGTCTGCTGGATGACTTTGGT GGGCCGCAGCCTGGGCCTTTCCTACAGAGT  Q  P  P  T  P  P  P  P  P  R   R  S  L  S  L  L  D  D  F  G   G  P  Q  P  G  P  F  L  Q  S  GGTGTTCATCCCTCCCATGCCTTCATCCAG CATAGCCTCAGCCTCAATGATACGTTCCTG CGGGGTCTCCCCCGGCCCATCCCGCAGCCT  G  V  H  P  S  H  A  F  I  Q   H  S  L  S  L  N  D  T  F  L   R  G  L  P  R  P  I  P  Q  P  GGCGACGTCCAGAACCCATCCCGGGTGGAC CAGCGCCCACTGCTGTGCCCACTGAGCCGC CCCGATGCCAGCAGCTTTGCCACCAGCCTC  G  D  V  Q  N  P  S  R  V  D   Q  R  P  L  L  C  P  L  S  R   P  D  A  S  S  F  A  T  S  L  CGAGAACTGGAAAAATGTGGCTGGTACTGG GGTCCTATGAACTGGGAGGATGCAGAGATG AAGCTGAAGGCCAAGCCAGACGGGGCGTTT  R  E  L  E  K  C  G  W  Y  W   G  P  M  N  W  E  D  A  E  M   K  L  K  A  K  P  D  G  A  F  CTGGTGAGGGACAGCTCTGACCCCCGCTAC ATCCTCAGCCTGAGCTTCCGCTCCCAGGGG GTCACACACCACACGCGCATGGAGCACTAC  L  V  R  D  S  S  D  P  R  Y   I  L  S  L  S  F  R  S  Q  G   V  T  H  H  T  R  M  E  H  Y  AGAGGAACCTTCAGCCTCTGGTGTCACCCA AAGTTTGAGGACCGCTGTCATTCTGTGGTA GAGTTTATCGAACGGGCCATCATGCACTCC  R  G  T  F  S  L  W  C  H  P   K  F  E  D  R  C  H  S  V  V   E  F  I  E  R  A  I  M  H  S  AAGAACGGAAAATTCCTCTACTTCCTGCGT TCACGGGTCCCAGGCCTCCCCCCGACCCCG GTCCAGCTACTGTACCCAGTGTCCCGCTTC  K  N  G  K  F  L  Y  F  L  R   S  R  V  P  G  L  P  P  T  P   V  Q  L  L  Y  P  V  S  R  F  AGCAGTGTCAAGTCCCTGCAGCACCTCTGT CGCTTCTGCATTCGCCAGCTGATCCGCATA GACCACATCCAGGAGCTCCCGTTGCCCACG  S  S  V  K  S  L  Q  H  L  C   R  F  C  I  R  Q  L  I  R  I   D  H  I  Q  E  L  P  L  P  T  CCCCTCATAGTCTACCTGCGAAAGTTCTAC TACTATGATCCTGAGGAGGAGATGTACATG TCAATCAAGGAGATGGGGCGGGACACGACC  P  L  I  V  Y  L  R  K  F  Y   Y  Y  D  P  E  E  E  M  Y  M   S  I  K  E  M  G  R  D  T  T  ACCCAACCGGCGACTGGCCAGCCGGAGTCT CAAACGTAGCATTTGGAAGATCATTGGAGC AAGGTTTTTATACACGTGGACAGCTTACCT  T  Q  P  A  T  G  Q  P  E  S   Q  T  *    GAAGACGGATGGTGACTGATTGGGGACAAT CTCGCT |
| --- | --- |

**Fig. S12 The cDNA sequence and its translation of rainbow trout SOCS7a2 gene.** The primers used for cDNA cloning are boxed. The start and stop codons are highlighted in red. The arrows indicate intron positions.

**Fig. S13**

| 1 1 93 12 183 42 273 72 363 102 453 132 543 162 633 192 723 222 813 252 903 282 993 312 1083 342 1173 372 1263 402 1353 432 1443 462 1533 492 1623 522 1713 552 1803 582 1893 612 1983 642 2073 672 2163 702 2253 732 2343 762 2433 792 2523 822 2613 | GCGGTAGCTATGTGAAGCGTAGAACTCGTTTATTCCCTTTCGTGAAT**TGA**GGAATAATT**ATG**AACAACGCGCAAGATATGTCTCCCGATTTT    M  N  N  A  Q  D  M  S  P  D  F  GTCTTGATGCGCCTGGTTTCCGCGGCCGAG GATGACCGCTTGGACGAGGAGAATGGGAAT CTGTCGTCGGGTGGAGTGGTGGCAGGACTC  V  L  M  R  L  V  S  A  A  E   D  D  R  L  D  E  E  N  G  N   L  S  S  G  G  V  V  A  G  L  GGTAAAGAGGTCTTGGCTCATAGCAACATG AAAGGGGGCTTGGAATTCGCCCGAGATCCC ACGACAGGCCTCGCGCATTCCGGCAATCTC  G  K  E  V  L  A  H  S  N  M   K  G  G  L  E  F  A  R  D  P   T  T  G  L  A  H  S  G  N  L  TCTTCGCTGAACGATGCACAGCAGAGCGGC GGAACGGCGGCACCTGACAGCCGGGTGATG GCGACTAAACCGTCGGTGTCTATTCCTCCT  S  S  L  N  D  A  Q  Q  S  G   G  T  A  A  P  D  S  R  V  M   A  T  K  P  S  V  S  I  P  P  CCACACAGCGCCATGGAGGCCAAGGGCTTT GAGTTTGCTCACAGAGGCGGCTTACGGCCT CAGCTCCTTGTGTTTCCTAACATATTGAGG  P  H  S  A  M  E  A  K  G  F   E  F  A  H  R  G  G  L  R  P   Q  L  L  V  F  P  N  I  L  R  GATGGTGAGGGGATTTTAGAATGTGAATCC GACGGTCGGAATCAGTCGAGGAAACATATT GGTTTGGACAAGACCTCCGGCTCTGTCTCC  D  G  E  G  I  L  E  C  E  S   D  G  R  N  Q  S  R  K  H  I   G  L  D  K  T  S  G  S  V  S  GACCTAACCGGAGGCATCAATAACAATACG CTCAGTGCCGGCGACGCGCAACAGCAGAAC CATCTGTCCCAGAGCTGGGGTCTGCATCCC  D  L  T  G  G  I  N  N  N  T   L  S  A  G  D  A  Q  Q  Q  N   H  L  S  Q  S  W  G  L  H  P  CGGGTGGTATTATCCACGGTTGCTGCCGAT ATCGAGGGAGGTGAGCTATGCCACCGGCAC AGTTTGATCACCAACCCATCAGACTGGCCT  R  V  V  L  S  T  V  A  A  D   I  E  G  G  E  L  C  H  R  H   S  L  I  T  N  P  S  D  W  P  CCCTTAAGGGATAAATCCAATCATTTCACA ATGATAGAGTCGAAATGGGGGTGCACAACC GGGGATCTGCCCGACGGCCCAGTGTTCGAC  P  L  R  D  K  S  N  H  F  T   M  I  E  S  K  W  G  C  T  T   G  D  L  P  D  G  P  V  F  D  CTGGCCAGAAGGTTCGGGGAGCTGGGGCTC GGCGCGGTACCCAAGATATTATTCAAGGAC GGGGCGATGCCCCAGTGCTCGTGTCAGGGC  L  A  R  R  F  G  E  L  G  L   G  A  V  P  K  I  L  F  K  D   G  A  M  P  Q  C  S  C  Q  G  GCACATGGTCCGGCACCAGCGGGGATGGGG CATGGGGATGACCCCACTGAGACTAGCGAT GCTTTGTTGGTGCTAGAGGGGCTGGGGACT  A  H  G  P  A  P  A  G  M  G   H  G  D  D  P  T  E  T  S  D   A  L  L  V  L  E  G  L  G  T  GGGGACGTGGCCAGGCTGGGTATCACTGGA TGCCCAGAGGATGATTCGGATGAAAGTCGA GCACGTCGAGTTCAGAAGATGTCAGGTGCA  G  D  V  A  R  L  G  I  T  G   C  P  E  D  D  S  D  E  S  R   A  R  R  V  Q  K  M  S  G  A  TTTTCTCTCAGTAGCTTTCAGGCAGAGTTG ACCAGACAGATGGAAGGGGTGGCTGGAGAA CCTTGTCCGTCGGCGCACGGTGACGCACAA  F  S  L  S  S  F  Q  A  E  L   T  R  Q  M  E  G  V  A  G  E   P  C  P  S  A  H  G  D  A  Q  GTATGTAGCCTGCATGGAAACGTACCTAAC CCAACTCAACCGAGCCCGGGGGATACAGAA CAGAATCCGGAGGTGTCGACATTACCTGCG  V  C  S  L  H  G  N  V  P  N   P  T  Q  P  S  P  G  D  T  E   Q  N  P  E  V  S  T  L  P  A  GCTACACCGAGCTCGGACCGACCTGTCAGT CCAACCCCTAGCCAGGCATCTACACCCCGG AAGCGGGCAGACAAGTGCGTCAGTGAGCCC  A  T  P  S  S  D  R  P  V  S   P  T  P  S  Q  A  S  T  P  R   K  R  A  D  K  C  V  S  E  P  CGGACTCCAACCGGTCGGGTCGACAAAACA CTCAAAGTTCCAGGGAAGTCCAGAAAGGGC TCACTTAAAATACGCCTGAGTAAACTTTTC  R  T  P  T  G  R  V  D  K  T   L  K  V  P  G  K  S  R  K  G   S  L  K  I  R  L  S  K  L  F  AGAACTAAAAGCTGTAGTGGTTCCAATAGC CTTCTGGATAAGAGGCCATCAGTGGCCTAT TCAATCTCCTCCGCTGGAAGTCTGATTGAC  R  T  K  S  C  S  G  S  N  S   L  L  D  K  R  P  S  V  A  Y   S  I  S  S  A  G  S  L  I  D  ATGGCCAGTGGAAGTGGTGGGGAGCATGAC GTGGACAGCCAACCCCGAATGACCAGGGCC CAAAGTGCTTTCTCTCCTGCTTCCTTTGCT  M  A  S  G  S  G  G  E  H  D   V  D  S  Q  P  R  M  T  R  A   Q  S  A  F  S  P  A  S  F  A  CCTTTCACTGGCGAGACTGTTTCATTGGTG GATGTGGATATTTCGAGGAGAGGGGCGAAC ACTCCGCACCCTCCCACGCCTCCACCTCCG  P  F  T  G  E  T  V  S  L  V   D  V  D  I  S  R  R  G  A  N   T  P  H  P  P  T  P  P  P  P  CCACGCAGAAGTCTCAGTCTATTAGATGAC ATAGGTGGGCCGCAGCCTGGTCCTTTCCTA GTGAGTGTTATGGGGGCCTCCCTACAGTCC  P  R  R  S  L  S  L  L  D  D   I  G  G  P  Q  P  G  P  F  L   V  S  V  M  G  A  S  L  Q  S  CTTCCCCTGCCTCTTCCTCCTCCTCATCCC TCCCATGCCACCATCCAGCATAGTATCAGC CTCAATGATGCATTCCTCCGGGCCCTGCCT  L  P  L  P  L  P  P  P  H  P   S  H  A  T  I  Q  H  S  I  S   L  N  D  A  F  L  R  A  L  P  CACTCCACCTCTTCACAGCTCCTCTCAGCT CCTCCCCCTTCCAGGTTGGCCCCGCCCCCA AGGCTCTGTCCTCTGAGGCGGCCTGAGGCC  H  S  T  S  S  Q  L  L  S  A   P  P  P  S  R  L  A  P  P  P   R  L  C  P  L  R  R  P  E  A  AGCAACTTCACCGCTAGTCTGAGAGAGCTG GAAAAATGTGGCTGGTATTGGGGTCCTATG AACTGGGAAGATGCAGAGATGAAGTTGAAG  S  N  F  T  A  S  L  R  E  L   E  K  C  G  W  Y  W  G  P  M   N  W  E  D  A  E  M  K  L  K  GGGAAGCCTGACGGGGCGTTCCTGGTGAGG GACAGCTCTGACCCCCGCTACATCCTCAGC CTCAGCTTCCGCTCCCAGGGAGTTACACAC  G  K  P  D  G  A  F  L  V  R   D  S  S  D  P  R  Y  I  L  S   L  S  F  R  S  Q  G  V  T  H  CACACGCGCATGGAGCACTATAGAGGGACA TTCAGCTTGTGGTGTCATCCCAAGTTTGAG GACCGATGTCATTCTGTGGTGGAGTTTATC  H  T  R  M  E  H  Y  R  G  T   F  S  L  W  C  H  P  K  F  E   D  R  C  H  S  V  V  E  F  I  GAGCGAGCCATCATGCACTCCAAAAATGGA AAATCCCTCTACTTCCTGCGTTCACGTGTG CCAGGGCTCCCCCCTACCCCGGTGCAGCTG  E  R  A  I  M  H  S  K  N  G   K  S  L  Y  F  L  R  S  R  V   P  G  L  P  P  T  P  V  Q  L  CTCTACCCAGTGTCCCGGTTCAGCAACGTC AAGTCCCTGCAGCACCTCTGTCGCTTCTGC ATCCGACAAATGGTCCGCATCGACCACATC  L  Y  P  V  S  R  F  S  N  V   K  S  L  Q  H  L  C  R  F  C   I  R  Q  M  V  R  I  D  H  I  CAGGAGCTGCCGCTGCCCAAACCACTGATC ATGTACCTGAGGAAGTTCTACTACTATGAT GCAGAGGTAGAGATGTACCTGTCAATCAAG  Q  E  L  P  L  P  K  P  L  I   M  Y  L  R  K  F  Y  Y  Y  D   A  E  V  E  M  Y  L  S  I  K  AGCATTCGACCAGGGGCTGGAGTAGAGAAA GAGGCGGAGTCTCAGACG**TAA**CTGGAAGAG GTGTCTGCTGGTCTGCCAGTCTTCCAGCAT  S  I  R  P  G  A  G  V  E  K   E  A  E  S  Q  T  *    CAGCCAATCAAAGTCCTCGGCACCCTCTGA ATCCAGTCGGCTGCAGTCAAAGGGGTCCAG ATTACCTAGCCCTTCTCTTGACTTTGG |
| --- | --- |

**Fig. S13 The cDNA sequence and its translation of rainbow trout SOC7b1 gene.** The primers used for cDNA cloning are boxed. The start and stop codons, and the immediate upstream in-frame stop codon of the main ORF are highlighted in red. The arrows indicate intron positions.

**Fig. S14**

| 1 1 93 12 183 42 273 72 363 102 453 132 543 162 633 192 723 222 813 252 903 282 993 312 1083 342 1173 372 1263 402 1353 432 1443 462 1533 492 1623 522 1713 552 1803 582 1893 612 1983 642 2073 672 2163 702 2253 732 2343 762 2433 792 2523 822 2613 2701 | GCGGTAGCTATGTGAAGCGTTGAAATCGTTTATTCCCTTTCGTGAATTGAGGAATAATT**ATG**AACAACGCGCAAGATATGTCTCCCGATTTT   M  N  N  A  Q  D  M  S  P  D  F  GTCTTGATGCGCCTGGTTTCCGCGGCCGAG GATGACCGCTTAGACGAGGAGAATGGGAAT CTGTCGTCGGGTGGAGTGGTGGCAGGACTC  V  L  M  R  L  V  S  A  A  E   D  D  R  L  D  E  E  N  G  N   L  S  S  G  G  V  V  A  G  L  GGTAAAGAGGTCTTGGCTCATAGCAACATG AAAGGGGGCTTGGAATTCTCCCGAGATCCC AAGACAGGCCTCGCGCATTCCGGGCATCTC  G  K  E  V  L  A  H  S  N  M   K  G  G  L  E  F  S  R  D  P   K  T  G  L  A  H  S  G  H  L  TCTTCGCTGAACAATGCACAACAGAGCTGC GGAACGGCGGCACCTGACACCGGGGTGATG GCAACTAGACCTTCGGTGTCTATTACTCTT  S  S  L  N  N  A  Q  Q  S  C   G  T  A  A  P  D  T  G  V  M   A  T  R  P  S  V  S  I  T  L  CCTCCACAGAGCGCTATGGAGGCCAAGGGC TTTGAGTTCTCTCACAGAGGCGGCTTACGG CCTCAGCTCCTTGTGTTTCCTAACATATTG  P  P  Q  S  A  M  E  A  K  G   F  E  F  S  H  R  G  G  L  R   P  Q  L  L  V  F  P  N  I  L  AGGGATGGTGAGGATATTTTAGACCGTGAA TCCGACGGTCGGAATCAGTCGAGGGAACAT ATTGGTTTGGACAAGACCTCCGGCTCTGTC  R  D  G  E  D  I  L  D  R  E   S  D  G  R  N  Q  S  R  E  H   I  G  L  D  K  T  S  G  S  V  TCCGACATAAGCGGAGGCATCAATAACAAC ACGCTCAGCGCCGGCGACACGCAACAGCAG AACCATCTGTCCCGGAGCTGGGGTCTGCAT  S  D  I  S  G  G  I  N  N  N   T  L  S  A  G  D  T  Q  Q  Q   N  H  L  S  R  S  W  G  L  H  CCCCGGGTGGCATTATCCACGGTTGCTGCC GATATCGAGGGAGGTGAGCTATGTCATCGG CACCGTTTGATCACCAACCCATTAGACTGG  P  R  V  A  L  S  T  V  A  A   D  I  E  G  G  E  L  C  H  R   H  R  L  I  T  N  P  L  D  W  CCTCCCTTATCGGATAAATCCAATCCTCTC ACCATGATAGAGTCGAAATGGGGGTGCACA ACCGGGGAACTGCCCAACGGCCCAGTGTTC  P  P  L  S  D  K  S  N  P  L   T  M  I  E  S  K  W  G  C  T   T  G  E  L  P  N  G  P  V  F  GACCTGGCCAGAAGGTTCGGGGAGCTGGGG CTCGGCGCGGTACCCAAGATATTATTCAAG GACGGGGAGATGCCCCAGTGCTCGTGTCAG  D  L  A  R  R  F  G  E  L  G   L  G  A  V  P  K  I  L  F  K   D  G  E  M  P  Q  C  S  C  Q  GGTGCACATGGGCCGGCACCTGCGGGGATG GGGCATGGGGATGACCCGACTGAGACTAGC GATGCTTTGTTGGTGCTGGAGGGGCTGGGG  G  A  H  G  P  A  P  A  G  M   G  H  G  D  D  P  T  E  T  S   D  A  L  L  V  L  E  G  L  G  ACTGGGGATGTGGGTATCACTGGCTGCCCG GAGGACGATTCGGATGAAAGTCGAGCACGT CGAGTTCAAAAGATGTCCGGTGCATTTTCT  T  G  D  V  G  I  T  G  C  P   E  D  D  S  D  E  S  R  A  R   R  V  Q  K  M  S  G  A  F  S  CTCAGTAGCTTTCAAGCAGAGTTGACCAGA CAGATGGAAGGGGTGGCTGGAGAACCTTGT CCGTCGGCGCACGGTGACGCACAAGTATGT  L  S  S  F  Q  A  E  L  T  R   Q  M  E  G  V  A  G  E  P  C   P  S  A  H  G  D  A  Q  V  C  AGCCTGCATGGAAACGCACCTAACCCAACT CAACCGAGCCCGGGGGATATAGAACAGAAT CCGGAGGTGTCGAAATTACCTGGGGCCTCT  S  L  H  G  N  A  P  N  P  T   Q  P  S  P  G  D  I  E  Q  N   P  E  V  S  K  L  P  G  A  S  GCGGCTACACCAAGCTCGGACCGACCTGTC AGTCCAAACCCTAGCCAGGCATCAACACCC CGGAAGCGGGCAGACAAGTGCGTCAGTGCG  A  A  T  P  S  S  D  R  P  V   S  P  N  P  S  Q  A  S  T  P   R  K  R  A  D  K  C  V  S  A  CCCCGGACTCCAAACGGTCGGGGAAGCGAC GGAGAGAAAACACTAAAAGTTCCAGGGAAG TCCAGAAAGGGTTCACTTAAAATACGCCTG  P  R  T  P  N  G  R  G  S  D   G  E  K  T  L  K  V  P  G  K   S  R  K  G  S  L  K  I  R  L  AGTAAACTTTTCAGAACTAAAAGCTGTAGT GGTTCCAATACCCTTCTGGATAAGAGGCCA TCAGTGGCCTTTTCAATCTCCTCCGCTGGA  S  K  L  F  R  T  K  S  C  S   G  S  N  T  L  L  D  K  R  P   S  V  A  F  S  I  S  S  A  G  AGTCTGACGGATATGGCCAGTGGAAGTGGT GGGGAGCATGACGTGGACAGCCAACCCAGA ATGACCAGGGCCCAAAGTGCCTTCTCTCCT  S  L  T  D  M  A  S  G  S  G   G  E  H  D  V  D  S  Q  P  R   M  T  R  A  Q  S  A  F  S  P  GCTTCCTTTGCTCCTTTCACTGGTGAGACT GTTTCATTGGTGGATGTGGATATTTCGAGG AGAGGAGCGAACACTCGGCACCCTCCCACG  A  S  F  A  P  F  T  G  E  T   V  S  L  V  D  V  D  I  S  R   R  G  A  N  T  R  H  P  P  T  CCTCCACCTCCGCCACGCAGAAGTCTCAGT CTATTAGATGACATAGGTGGGCCGCAGCCT GGTCCTTTCCTAGTGGGTGTTATGGGGGCC  P  P  P  P  P  R  R  S  L  S   L  L  D  D  I  G  G  P  Q  P   G  P  F  L  V  G  V  M  G  A  TCCCTACAGTCCCTCCCCCTGCCTCTTCCT CCTCCTCATCCCTCCCATGCCACCATCCAG CATAGTATCAGCCTCAATGATGCATTCCTC  S  L  Q  S  L  P  L  P  L  P   P  P  H  P  S  H  A  T  I  Q   H  S  I  S  L  N  D  A  F  L  CGGGCCCTGCCTCACTCCACCTCTTCACAG CTCCTCTCAGCTCCTCCCTCTTCCAGGTTG GCCCCGCCCCCAAGGCTCTGTCCTCTGAGG  R  A  L  P  H  S  T  S  S  Q   L  L  S  A  P  P  S  S  R  L   A  P  P  P  R  L  C  P  L  R  CGGCCTGAGGCCAGCAACTTCACCGCTAGT CTGAGAGAGCTGGAAAAGTGCGGCTGGTAT TGGGGTCCTATGAACTGGGAAGATGCAGAG  R  P  E  A  S  N  F  T  A  S   L  R  E  L  E  K  C  G  W  Y   W  G  P  M  N  W  E  D  A  E  ATGAAGTTGAAGGGGAAGCCTGACGGGGCG TTCCTGGTGAGGGACAGCTCTGACCCCCGC TACATCCTCAGCCTCAGCTTCCGCTCCCAG  M  K  L  K  G  K  P  D  G  A   F  L  V  R  D  S  S  D  P  R   Y  I  L  S  L  S  F  R  S  Q  GGAGTCACACACCATACGCGCATGGAGCAC TACAGAGGGACATTCAGCTTGTGGTGTCAC CCCAAGTTTGAGGACCGATGTCATTCTGTG  G  V  T  H  H  T  R  M  E  H   Y  R  G  T  F  S  L  W  C  H   P  K  F  E  D  R  C  H  S  V  GTGGAGTTTATCGAGCGAGCCATCATGCAC TCCAAGAATGGAAAATTCCTCTACTTCCTG CGATCACGTGTGCCAGGGCTCCCCCCTACC  V  E  F  I  E  R  A  I  M  H   S  K  N  G  K  F  L  Y  F  L   R  S  R  V  P  G  L  P  P  T  CCGGTGCAGCTGCTCTACCCAGTGTCCCGG TTCAGCAACGTCAAGTCCCTGCAGCACCTC TGCCGCTTCTGCATCCGACAAATGGTCCGC  P  V  Q  L  L  Y  P  V  S  R   F  S  N  V  K  S  L  Q  H  L   C  R  F  C  I  R  Q  M  V  R  ATCGACCACATCCAGGAGCTGCCGCTGCCC AAACCACTGATCATGTACCTGAGGAAGTTC TACTACTACGATGCAGAGGAAGAGATGTAC  I  D  H  I  Q  E  L  P  L  P   K  P  L  I  M  Y  L  R  K  F   Y  Y  Y  D  A  E  E  E  M  Y  CTGTCAATCAAGAGCATTCGACCAGGGGCT GGAGTAGAACAAGTGGCCGAGTCTCAGACG TAACTGGACGAGGTGTCTGCTGGTCTGCCA  L  S  I  K  S  I  R  P  G  A   G  V  E  Q  V  A  E  S  Q  T   *    GTCTTCCAGCATCAGCCAATCAAAGTCCTC GGGCACCCTCTGTATCCAGTCAGCTGCAGT CAAAGGGATCCAGATGACCATGCCTTCTCT TGACTTTGA |
| --- | --- |

**Fig. S14 The cDNA sequence and its translation of rainbow trout SOCS7b2 gene.** The primers used for cDNA cloning are boxed. The start and stop codons, and the immediate upstream in-frame stop codon of the main ORF are highlighted in red. The arrows indicate intron positions.

**Fig. S15**

1. **Alignments of SOCS4 cDNA with SOCS4 genomic DNA (gDNA) sequences**

**Exon 1**

Trout-SOCS4-cDNA 1 ---------------------CATGGACATGGAGATTACGTTGTCTCTCTAAAACTATTT
Trout-SOCS4-gDNA 1 GATTATAATAGAATTTGGCAACATGGACATGGAGATTACGTTGTCTCTCTAAAACTATTT
Trout-SOCS4b-gDNA 1 CCAAATGTTGAGATTATAATATAATTTGGAAACATTTATGTTGACTTTCTAAAAACGACT
Salmon-SOCS4-gDNA 715696 TCAAATGGAGAGATTATAATATAATTTGGAAACATTTATGTTGACTCTCTA-AAACGATT

Trout-SOCS4a-cDNA TTAAAAG----------------------------------------------------- 46
Trout-SOCS4a-gDNA TTAAAAG**gt**aagaccatctctagacaaaagtaatcaaaagttaaatgatttgtaagattt 120
Trout-SOCS4b-gDNA TTGAAAG**gt**aagacagtctctagacaaacgtaatcaacagttaaacaatttagaagatag 120
Salmon-SOCS4-gDNA TTGAAAG**gt**aagacagtctctagacaaacgtaatcaacagttaaacgatttagaagatta 715814

**Exon 2**

SOCS4 ----------------CACAGAGCTGTATGAAGAAGAGGGAAGGCCATGTCTGAGAAGAA 44

SOCS4b ctgtccattgatgt**ag**cacagagctgtataaagaggagggaggaccatgtctgagaagaa 60

************* **** ****** * ****************

SOCS4 ATCCCGAAGTTCGGACATCTGTCCCAAATGCGGCATCCGCAGCTGGAGTGCCGATGGCTA 104

SOCS4b atcctggagttcagacagccgccccaaatgtggcatccgcagctggagtgcagacagcta 120

**** * ***** **** * * ******** ******************** ** ****

SOCS4 CGTGTGGAGCTGCAAGAAACGCTCCCGGAGTTCTCGAAACGATCCGGGCCTTCGGCGTCC 164

SOCS4b cgtgtggagcagcaagaaacgttccaggagttcccggaaaaaccccggcctttggggtcc 180

********** ********** *** ******* ** ** * ** ****** ** ****

SOCS4 GGAGGGGGTAGGGCTGATGGAGGAGCAAGGAGCGCGTTCCACCTCATGTCCGCGGAGACG 224

SOCS4b ggagggggagggaccgatgga---------tgaacgttccaccttgtgtccgtggcgatg 231

******** ** * ****** * ********** ****** ** ** *

SOCS4 GAGAGAGAGGAAGTGTAGCTGTACCGTAATGGGGGAAGTTGACATAGATGTCCCCTGTCG 284

SOCS4b gcgagagaggaagtgtagctgcaccgtcttgggggaaatcgacacggacgacacgtgtcg 291

* ******************* ***** ******** * **** ** * * * *****

SOCS4 GAAAGCCCTTTCTAGGCGCTCTCTCCGGCAGAAGTTCCAGGATGCAGTGGGTCAGTGTTT 344

SOCS4b aaaagccctttctcgacgctccctccggcagaagttccaggatgcggtgggtcagtgttt 351

************ * ***** *********************** **************

SOCS4 CCCTCTCCGCACTGACCATCGCCACCACCACCACGGCTGCCCAACGGGGGCCTACCAGGG 404

SOCS4b ccctctccgcaatggccaccatcacc---accacggccacccaacagggtcctccagggg 408

*********** ** *** * **** ******** ****** *** *** * ***

SOCS4 GGCCTTTTCTGTGCTCCTCTGGTCCAAGCGTCCGATACATGTCACGGAGCTCATGCAGGA 464

SOCS4b ggctttctctgtgctcctctggtccaagcgcaagatccacatctcggagctcatgcagga 468

*** ** *********************** *** ** ** ****************

SOCS4 CAAGTGCCCCTTCTCGTCCAAGTCAGAGCTGGCCCACTGCTGGCACCTCATCAAGAAGCA 524

SOCS4b caagtgtcccttctcacccaagtcggagctggctcactgctggcacctcatcaagaagca 528

****** ******** ******* ******** **************************

SOCS4 TGCCACCCACCCCAGCGCCATTGTGGGCCTAGAGGTTGCCCAAGCCGCCAAGGCTGCACA 584

SOCS4b cgccagccaccctagcaccatcgtgggcctagagactgcccaagctgctcaggcttccca 588

**** ****** *** **** ************ ********* ** ***** * **

SOCS4 GGCTGCTGGCAAAGAACCAGTCCCGTCCACTTCCACATCCCCGCCTTCGACACCTCTTTC 644

SOCS4b ggctgccagcaaagaacaattcccttccacgtcctcgtctccgccttcgatgcctctttc 648

****** ********* * **** ***** *** * ** ********** ********

SOCS4 ATGGGAGGGCATCTGCTTGAGTAGGCCCCTGAGCCTTGAGGACTGGGACCTCTCCCATCC 704

SOCS4b ctgggagggcatttgctcaagtaggcccctgagccttgaggactgggacccgtcctgtcc 708

*********** **** ******************************* *** ***

SOCS4 GCATGGCAGAGCAGCCTATGGTGGCAGCCATACAGATTACATCCTAGTCCCTGACCTCCT 764

SOCS4b acaaggaggagcagcccatggtgacagccataccgactacatccttgtcccggacctcct 768

** ** ******** ****** ********* ** ******** ***** ********

SOCS4 GCAGATCAACAACAGCTCGTGTTACTGGGGCGTTCTGGACCGCTTCCAGGCAGAGGAGCT 824

SOCS4b ccagatcaacaacagcccatgttactggggcgtgcgtgaccgcttcgaagccgaggagct 828

*************** * ************** * ********* * ** ********

SOCS4 CCTGGAAGGCCAGCCCGAGGGCACCTTCCTCCTCCGTGACTCAGCCCAGGACAAGTTCCT 884

SOCS4b tctggagggccagccagagggcaccttcctcctgcgcaactcggcccaggacgaattcct 888

***** ******** ***************** ** **** ********* * *****

**Fig. S15 continued**

SOCS4 CTTCTCCGTCAGCTTCCGCCGCTATAGCCGCTCCCTCCATGCGCGTATCGAGCAGAACGG 944

SOCS4b cttctcagtcagcttccgccgctacagccgttccctccatgcacgcatagagcagaacgg 948

****** ***************** ***** *********** ** ** ***********

SOCS4 TAAGCGCTTCAGCTTCGATGGCCGCGATCCGTGCATGTACCGGGATCCGAGTGTGACGGG 1004

SOCS4b caagcgcttcagctttgacggacgcgacccgtgcatttcctgggacccgagtgtcacggg 1008

************** ** ** ***** ******** * * **** ******** *****

SOCS4 CCTGCTCCGGCACTACAGCGACCCAGCCACATGCCTCTTCTTTGAGCCCCTCCTGTCCCG 1064

SOCS4b cctgctccggcactacagcgacccggccacatgccttttcttcgagcccctccttgctct 1068

************************ *********** ***** *********** * *

SOCS4 CCCTCTGGCCCGGACTTTCCCATTCACCCTGCAGCACCTGTGTCGCGCAGTGATCTGTAG 1124

SOCS4b ccccctgccccggactttccccttcaccctgcaacacctgtgccacgccgtgatctgtag 1128

*** *** ************* *********** ******** * *** ***********

SOCS4 CTGCACTACGTACCAGGGCATCAAGATCCTTCCACTGCCTCATCAGCTCAGGGACTATCT 1184

SOCS4b ctgcactacgtaccagggtattgagatccttccacttccctatcagctcaggtactatct 1188

****************** ** ************* ** *********** *******

SOCS4 TAGGCAGTACCACTACAAGTGCAATGGGGCTTATGCAGTGTAAAACAATGATAATTTCTG 1244

SOCS4b tagacagtaccactacaagtgcaatgtag----aacagtgtagaacaattataattacct 1244

*** ********************** * ******* ****** ********

SOCS4 ACTTCATAATTATAGTCCCATGGGTTACTACTCTTCAATATTGACCATGTTTCATTGGTT 1304

SOCS4b act--atcattattgtcccatgggttactactcttccatattgacaatgatttatccgtt 1302

*** ** ***** ********************** ******** *** ** ** ***

SOCS4 TTTAGAGACTTGTGAAGACCCTGACTGCTACAGTGCCTTCAGAAAGTATTCACACCCATA 1364

SOCS4b ttcagagacctgtgaagatccttcctgcagtcgcaagaagacttttgtagacttttttat 1362

** ****** ******** *** **** * *

SOCS4 GACTTTTTA-CACATTTTGTTGTGTTAAAATGGAGATTTTGTG-------TCACTGGCCT 1416

SOCS4b ttccttgtgggaaaatttaatcggacacacaagagacttgaatgggtgggggagggagag 1422

* ** * * * *** * * * * **** ** * *

SOCS4 ACACACAATATCAAGTAATGTCAAAGTGGAATTATGTTTTTAGAAATGT----------- 1465

SOCS4b tgagatgatttgaatgaatgtcagagtgaatgattacattattacagaaggtatgaggaa 1482

* * ** * ** ******* **** * * ** * *

SOCS4 ------------TTACAAATTAATTAAAAATTAAAAGCTGAAATGTCTTGAGTCAATAAG 1513

SOCS4b gtgcgcatctggtttaaaaactatcccatctggacaaatgtaacatcccaatacaataat 1542

** *** ** * * * * ** ** ** * ******

SOCS4 TATTCAACCCCTGGCAAGCCTAAATAAGTTCAGGAGAAACAATTTGCTTAACAACTCACA 1573

SOCS4b cattgc--------ctcccctactgccttcttacagtaactaaatgagcgataacactca 1594

*** * **** * ** *** * ** * *** * **

SOCS4 TAATAAGTTGCACGGACTCACTGTGTGAGGTAATAG-------------------- 1609

SOCS4b gaaacacttttagtatgtacatttacacatttaatgcaatatgtgaggacagttta 1650

**b. An alignment of trout SOCS4 and the predicted SOCS4b translation**

SOCS4 MSEKKSRSSDICPKCGIRSWSADGYVWSCKKRSRSSRNDPGLRRPEGVGLMEEQGARSTS 60

SOCS4b MSEKKSWSSDSRPKCGIRSWSADSYVWSSKKRSRSSRKNPGLWGPEGEGPMDE---RSTL 57

****** *** ***********.****.********::*** *** * *:* ***

SOCS4 CPRRRRERKCSCTVMGEVDIDVPCRKALSRRSLRQKFQDAVGQCFPLRTDHRHHHHGCPT 120

SOCS4b CPWRWRERKCSCTVLGEIDTDDTCRKALSRRSLRQKFQDAVGQCFPLRNGH-HHHHGHPT 116

** * *********:**:* * *************************..* ***** **

SOCS4 GAYQGAFSVLLWSKRPIHVTELMQDKCPFSSKSELAHCWHLIKKHATHPSAIVGLEVAQA 180

SOCS4b GSSRGAFSVLLWSKRKIHISELMQDKCPFSPKSELAHCWHLIKKHASHPSTIVGLETAQA 176

*: :*********** **::********** ***************:***:*****.***

SOCS4 AKAAQAAGKEPVPSTSTSPPSTPLSWEGICLSRPLSLEDWDLSHPHGRAAYGGSHTDYIL 240

SOCS4b AQASQAASKEQFPSTSSSPPSMPLSWEGICSSRPLSLEDWDPSCPQGGAAHGDSHTDYIL 236

*:*:***.** .****:**** ******** ********** * *:* **:*.*******

SOCS4 VPDLLQINN**SSCYWGVLDRFQAEELLEGQPEGTFLLRDSAQDKFLFSVSFRRYSRSLHAR** 300

SOCS4b VPDLLQINN**SPCYWGVRDRFEAEELLEGQPEGTFLLRNSAQDEFLFSVSFRRYSRSLHAR** 296

************ ***** ***:****************:****:*******************

**SH2 domain**

SOCS4 **IEQNGKRFSFDGRDPC**MYRDPSVTGLLRHYSDPATCLFFEPLLSRPLARTFPFTLQHLCR 360

SOCS4b **IEQNGKRFSFDGRDPC**ISWDPSVTGLLRHYSDPATCLFFEPLLALPLPRTFPFTLQHLCH 356

********************: ************************: ** ***********:

**Fig. S15 continued**

SOCS4 AVICSCTTYQGIKILPLPHQLRDYLRQYHYKCNGAYAV---------------------- 398

SOCS4b AVICSCTTYQGIEILPLPYQLRYYLRQYHYKCNVEQCRTIIITYYHYCPMGYYSSILTMI 416

************:*****:*** ********** .

**SOCS box**

SOCS4 ---------------------------------------------- 398

SOCS4b YPFSETCEDPSCSRKKTFVDFFISLWENLIGHTRDLNGWGRESEMI 462

**c. Identity )%)/similarity (%) of trout SOCS4 and the predicted SOCS4b translations**

|  | Full-length | N-terminal overlapping region | SH2 domain | SOCS box |
| --- | --- | --- | --- | --- |
| Identity | **70.6** | **83.2** | **90.8** | **89.2** |
| Similarity | **76.0** | **88.3** | **95.4** | **94.6** |

**Fig. S15 Analysis of a second SOCS4 locus in rainbow trout. (a) Alignments of SOCS4 cDNA with SOCS4b genomic sequences.** In the exon 1 alignment, trout SOCS4 cDNA was aligned with SOCS4 genomic DNA (acc no. CCAF010010822, reverse complement of region 2518-2637), trout SOCS4b genomic DNA (acc. no. CCAF010010823, reverse complement of region 61-180), and Atlantic salmon SOCS4 genomic DNA (acc. no. AGKD04000108). In the exon 2 alignment, trout SOCS4 cDNA was alignment with a trout SOCS4b genomic contig (acc. no. CCAF010010822, reverse complement of region 57181-58830). The nucleotide duplets in the intron junctions (GT/AG) are in red. The start and stop codons of the main ORF are highlighted in red. **(b) An alignment of trout SOCS4 and the predicted SOCS4b translations.** Dashes (-) indicate gaps in the alignment. The * and :/. indicate identical and similar amino acids, respectively. The SH2 domain is in bold and the SOCS box is underlined. The C-terminal translation of SOCS4b that resulted from ORF change is in red. (**c) Identity/similarity (%) of trout SOCS4 and SOCS4b translations.** The N-terminal overlapping region excludes the C-terminal region highlighted in red in panel (b).

**Fig. S16**

**
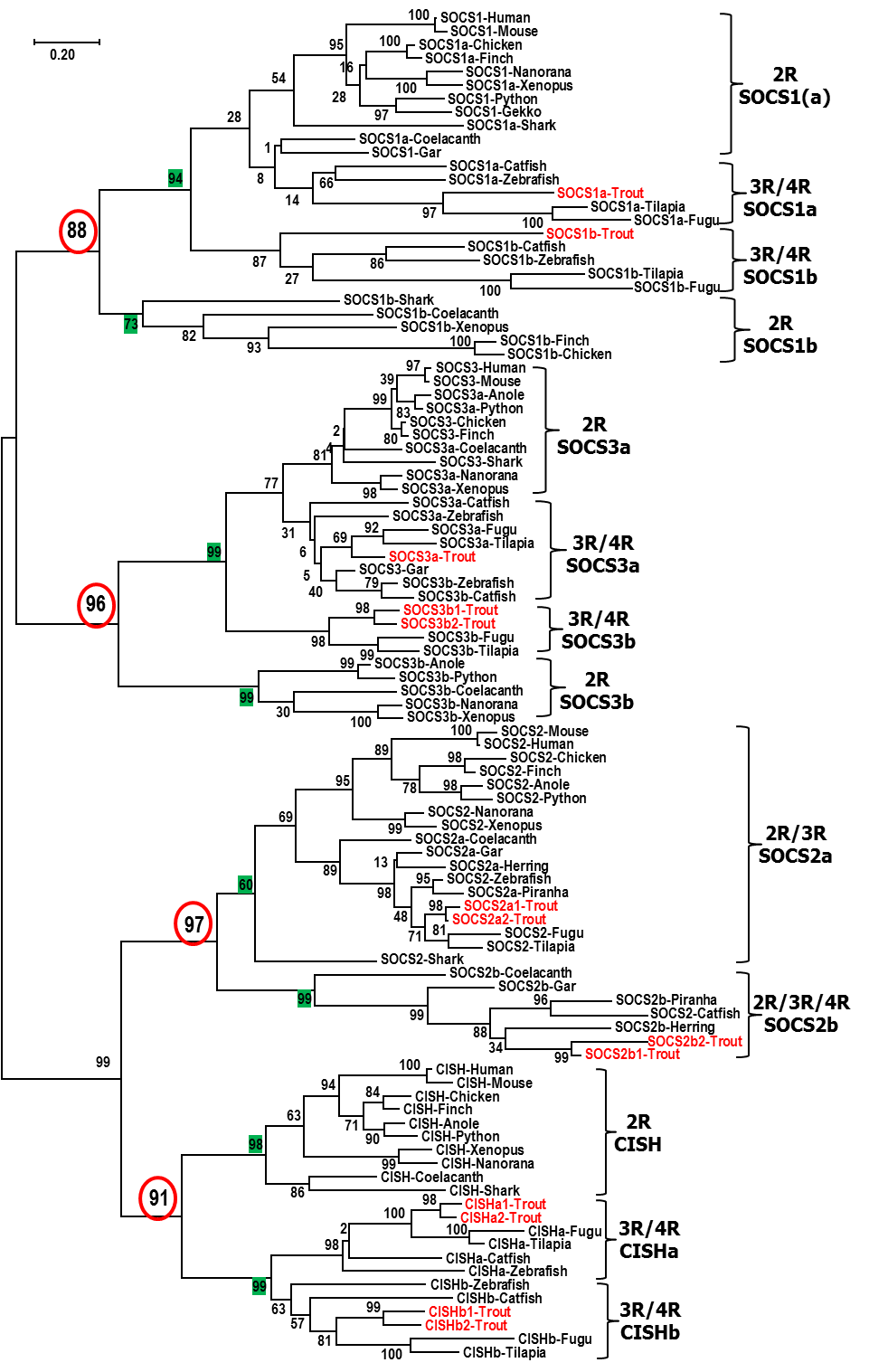
**

**Fig. 16 A maximum likelihood phylogenetic tree of vertebrate type II SOCS (SOCS1-3 and CISH).** The phylogenetic tree was constructed using amino acid multiple alignments generated by ClustalW and the maximum likelihood method within the MEGA7 program (68). The percentage of replicate trees in which the associated taxa clustered together in the bootstrap test (1,000 replicates) is shown next to the branches. The evolutionary distances were computed using the JTT matrix-based method with all sites used in the analysis. The amino acid sequences used are as described in Fig. 2 with the trout sequences in red.

**Fig. S17**


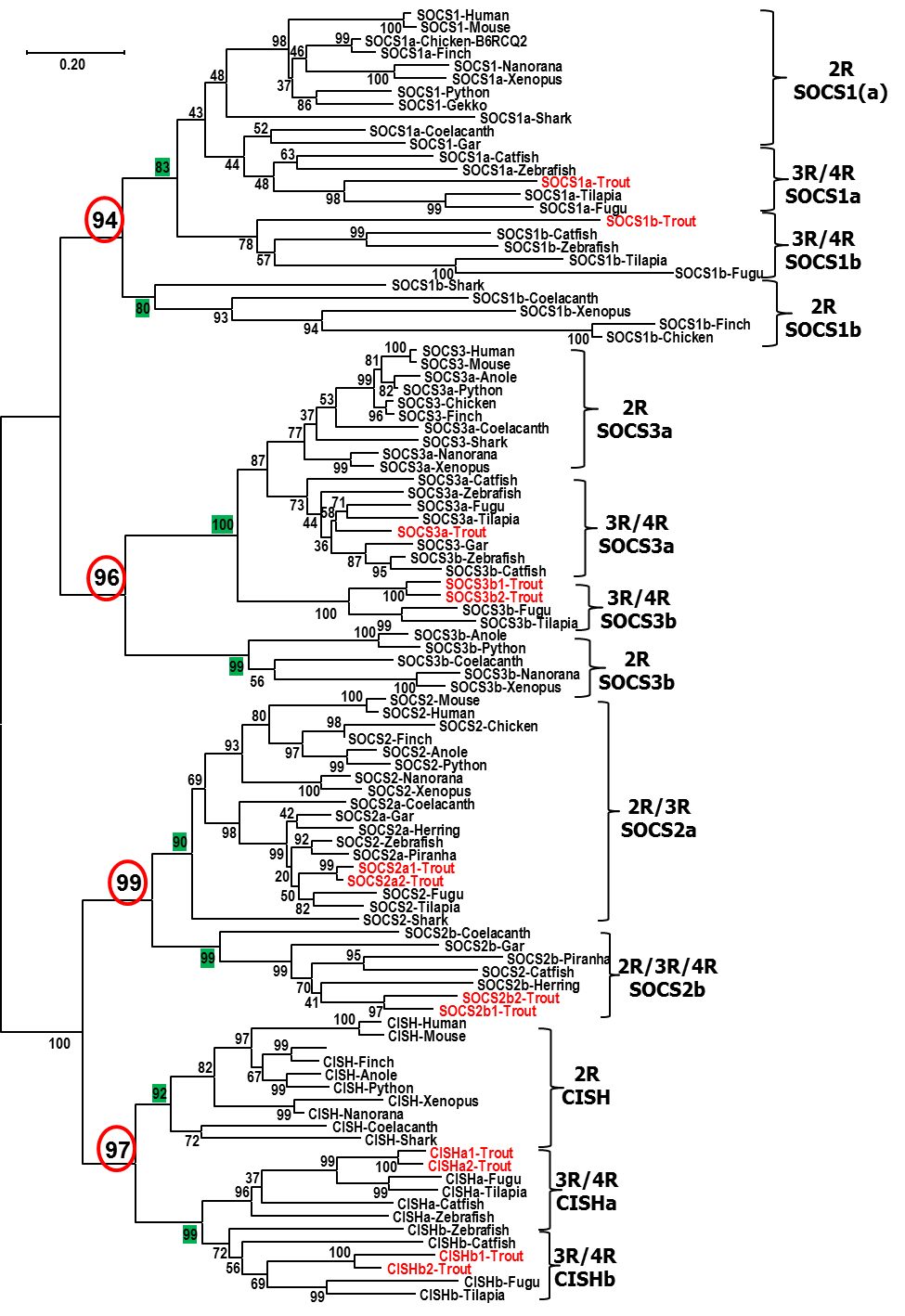


**Fig. 17 A minimum evolution phylogenetic tree of vertebrate type II SOCS (SOCS1-3 and CISH).** The phylogenetic tree was constructed using amino acid multiple alignments generated by ClustalW and the minimum evolution method within the MEGA7 program (68). The percentage of replicate trees in which the associated taxa clustered together in the bootstrap test (5,000 replicates) is shown next to the branches. The evolutionary distances were computed using the JTT matrix-based method with pairwise deletion option. The amino acid sequences used are as described in Fig. 2 with the trout sequences in red.

**Fig. S18**

**
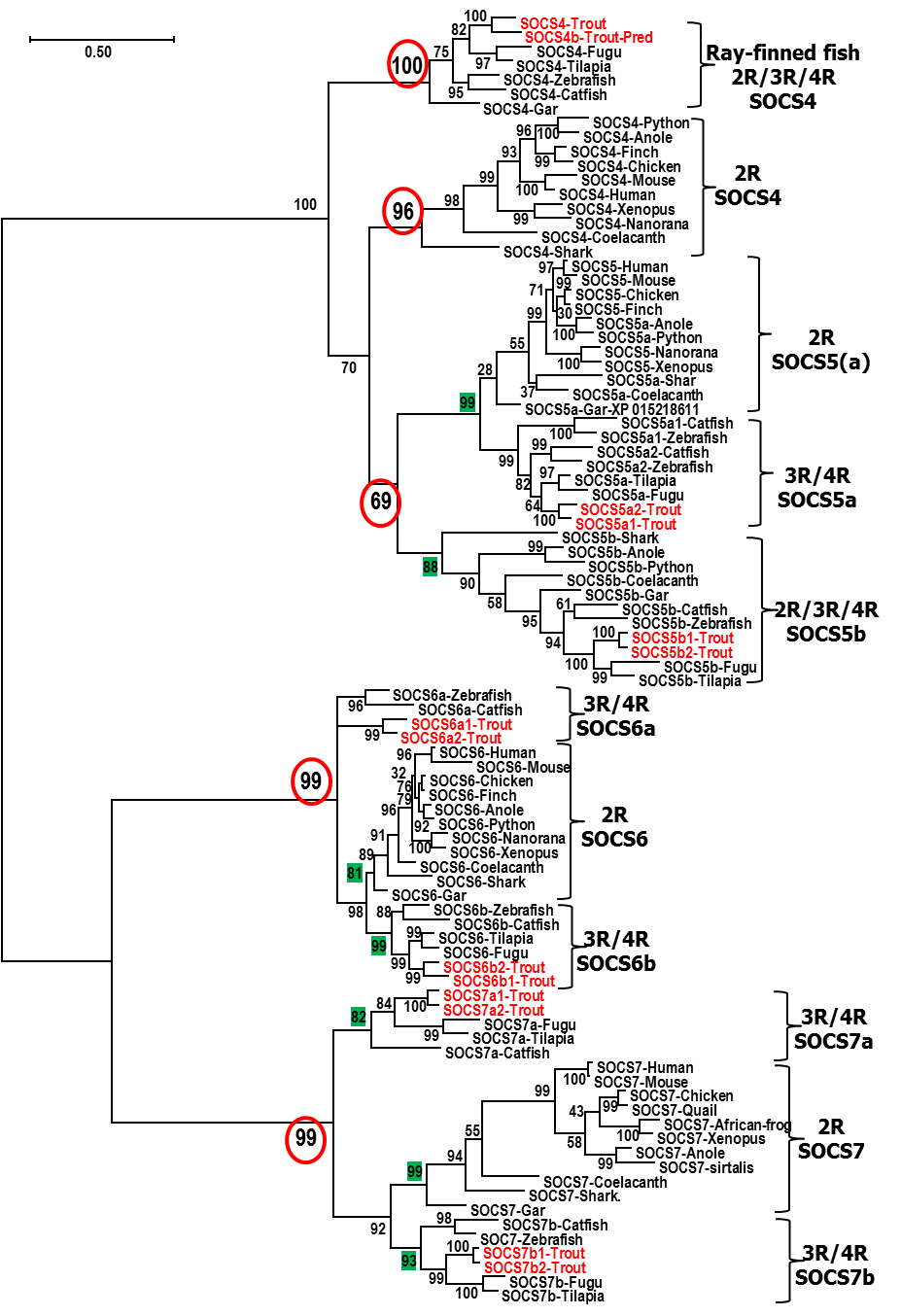
**

**Fig. 18 A maximum likelihood phylogenetic tree of vertebrate type I SOCS (SOCS4-7).** The phylogenetic tree was constructed using amino acid multiple alignments generated by ClustalW and the maximum likelihood method within the MEGA7 program (68). The percentage of replicate trees in which the associated taxa clustered together in the bootstrap test (1,000 replicates) is shown next to the branches. The evolutionary distances were computed using the JTT matrix-based method with 25% cutoff partial deletion option (with 20-40% cutoff showing similar topology). The amino acid sequences used are as described in Fig. 3 with the trout sequences in red.

**Fig. S19**

**
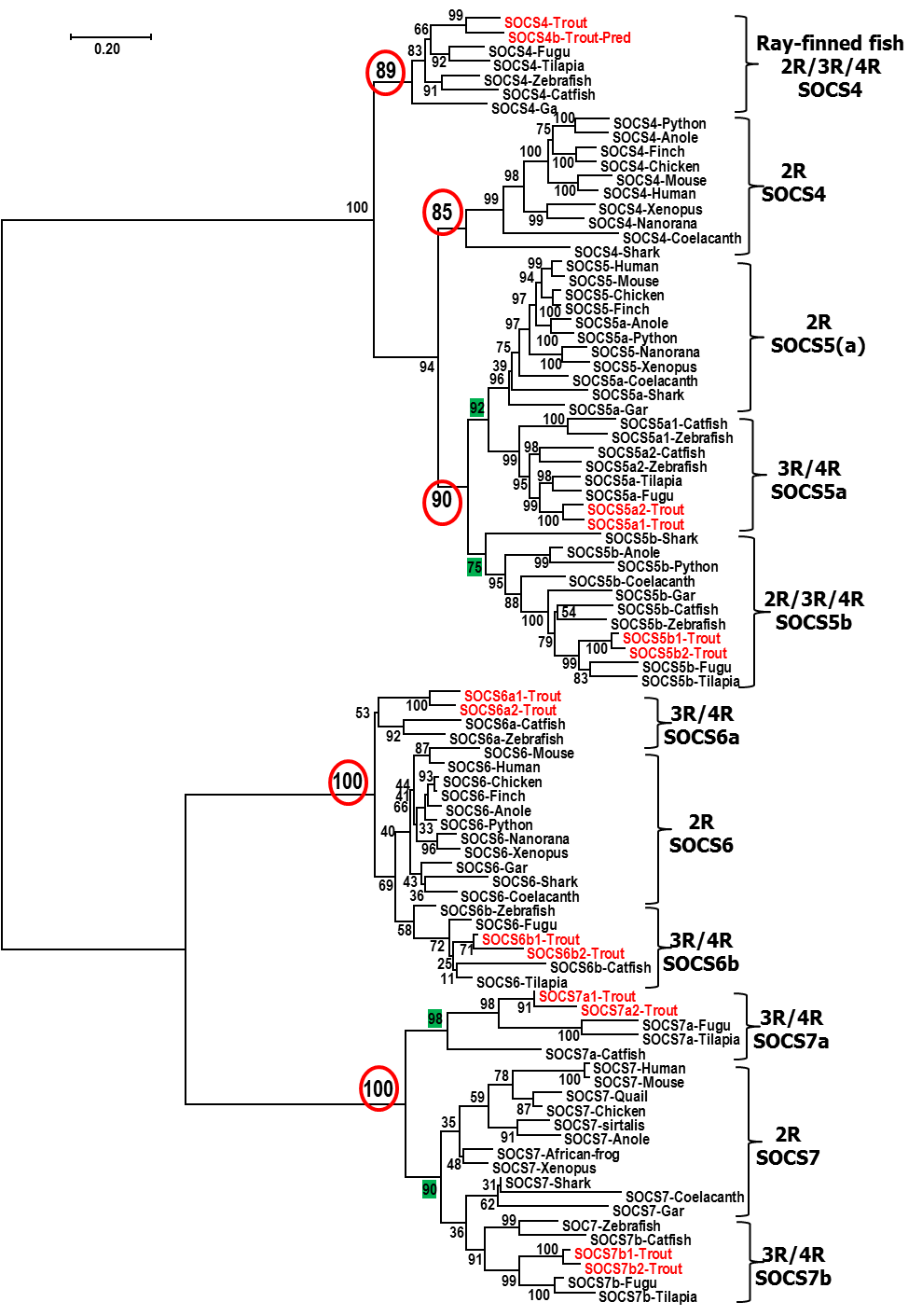
**

**Fig. 19 A minimum evolution phylogenetic tree of vertebrate type II SOCS (SOCS4-7).** The phylogenetic tree was constructed using amino acid multiple alignments generated by ClustalW and the minimum evolution method within the MEGA7 program (68). The percentage of replicate trees in which the associated taxa clustered together in the bootstrap test (5,000 replicates) is shown next to the branches. The evolutionary distances were computed using the JTT matrix-based method with pairwise deletion option. The amino acid sequences used are as described in Fig. 3 with the trout sequences in red.

**Fig. S20**


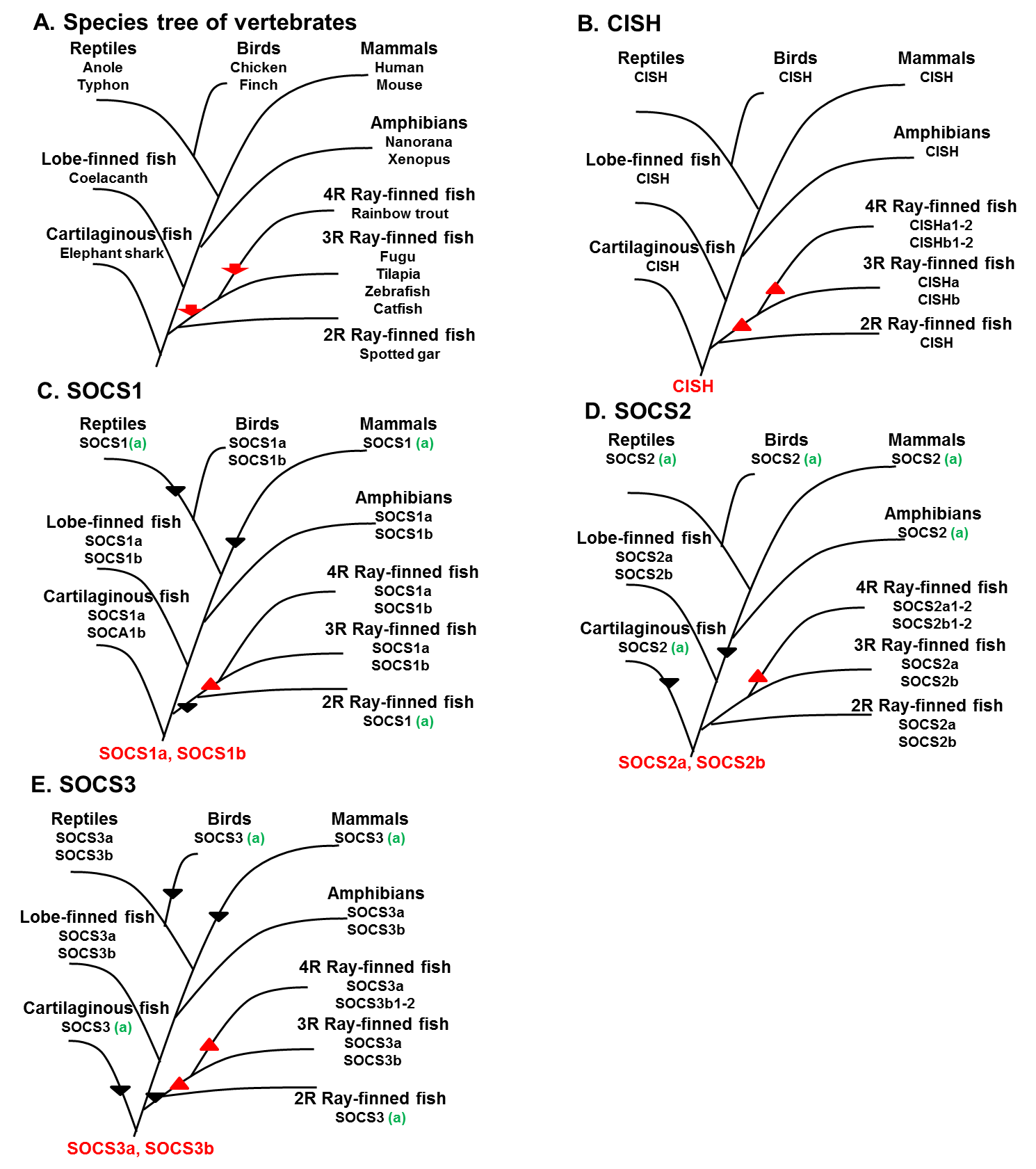


**Fig. S20 The type II SOCS genes in vertebrates and the coincidence of gene loss/gain in relation to 3R/4R WGDs.** (A) A species tree of vertebrates analysed in this study. The 3R and 4R WGDs are indicated by red arrows. (B) CISH in vertebrates. (C) SOCS1 in vertebrates. (D) SOCS2 in vertebrates. (E) SOCS3 in vertebrates. Black arrows indicate potential gene loss and red arrows indicate potential gene gain that coincide with the 3R/4R WGDs. The green “(a)” indicate its relatedness to the ancestral “a” paralogues.

**Fig. S21**


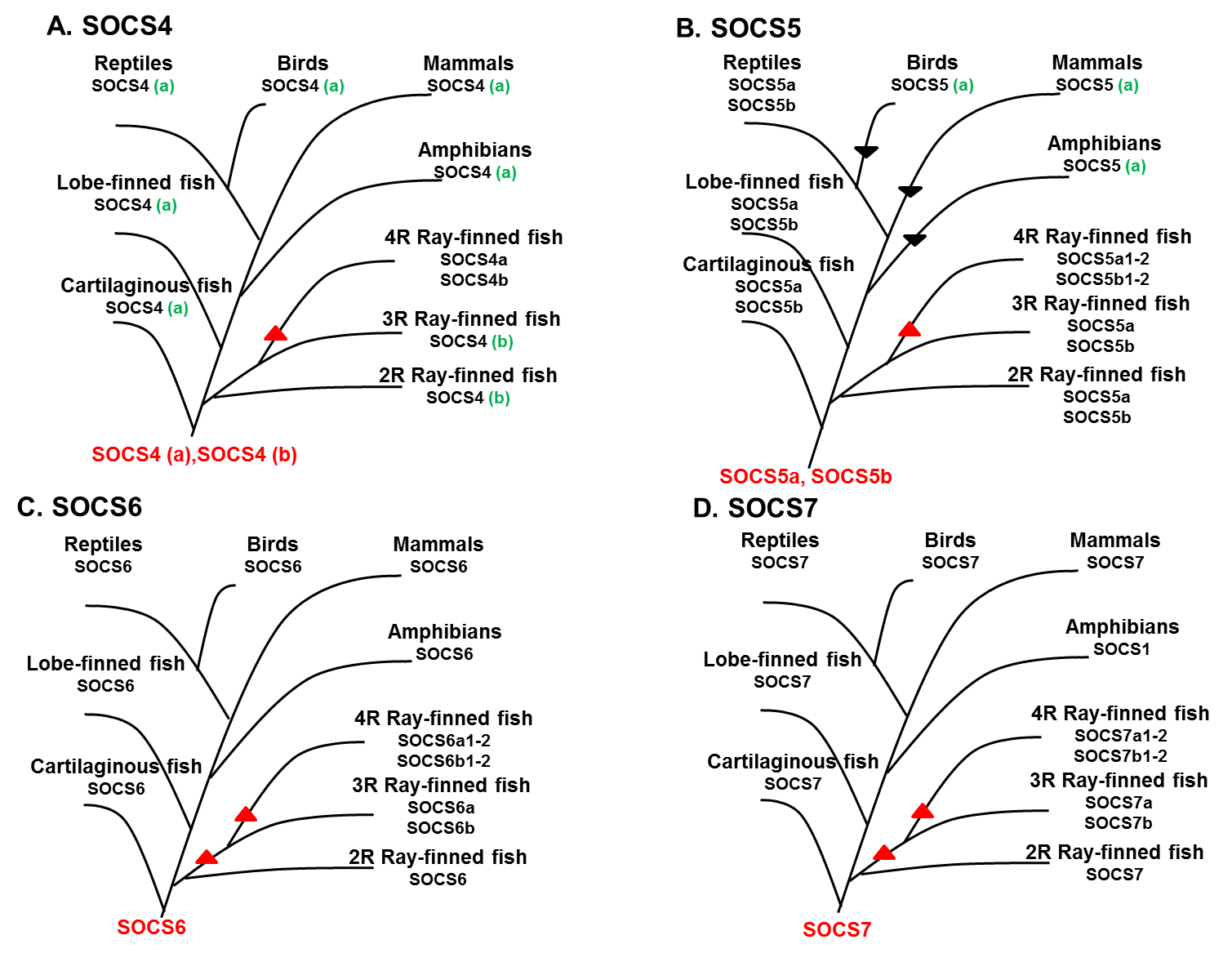


**Fig. S21 The type I SOCS genes in vertebrates and the coincidence of gene loss/gain in relation to 3R/4R WGDs.** (A) SOCS4 in vertebrates. (B) SOCS5 in vertebrates. (C) SOCS6 in vertebrates. (D) SOCS7 in vertebrates. Black arrows indicate potential gene loss and red arrows indicate potential gene gain that coincide with the 3R/4R WGDs. The green “(a)” or “(b)” indicate their relatedness to the ancestral “a” or “b” paralogues.

**Fig. S22**

**
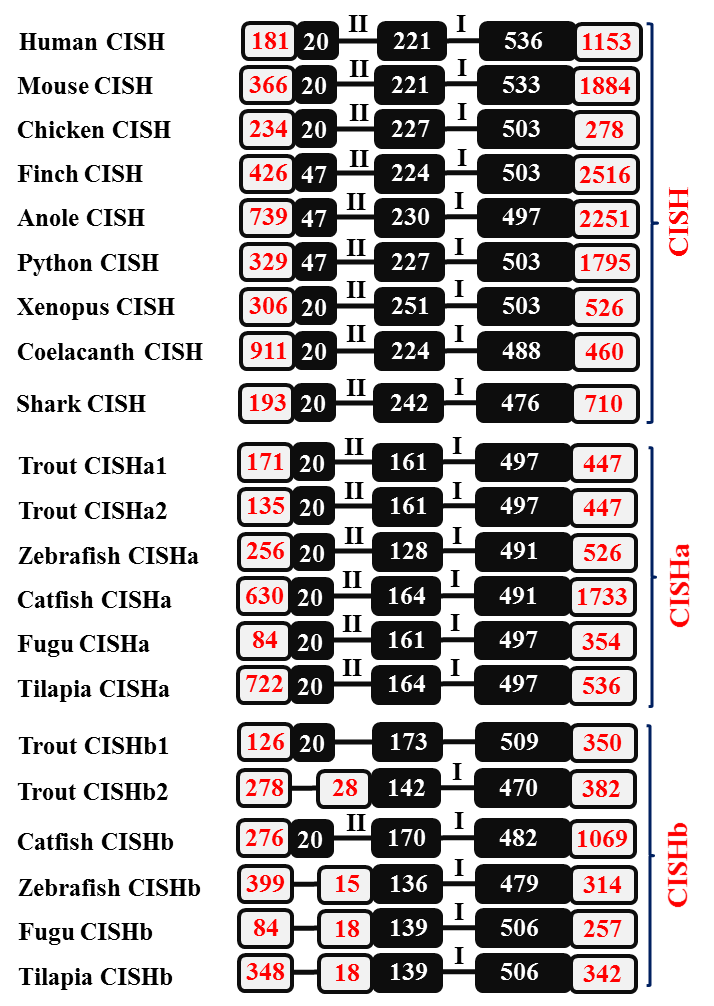
**

**Fig. S22 CISH gene organisation across vertebrates**. The gene organisation was predicted using the Spidey programme. The accession numbers of cDNA / genomic sequences used for prediction are NM_145071 / NC_000003 (human CISH), NM_009895 / AL672070 (mouse CISH), AF167297 / AADN04000453 (chicken CISH), XM_002190732 / ABQF01030557 (finch CISH), XM_003217562 / AAWZ02007093/AAWZ02007094 (anole CISH), XM_007430740 / AEQU02090666 (python CISH), XM_012960556 / AAMC03016793 (xenopus CISH), XM_005991736 / AFYH01035918 (coelacanth CISH**),** NM_001292833 / AAVX02006957 (shark CISH), AM903340 / CCAF010023507 (trout CISHa1), HG003693 / CCAF010055202 (trout CISHa2), EF195760 / CR847825 (zebrafish CISHa), XM_017480048 / LBML01006303 (catfish CISHa), EF195746 / CAAB02003702 (fugu CISHa), XM_003448311 and MKQE01000019 (tilapia CISHa), FR873795 / CCAF01004423 (trout CISHb1), HG003694 / CCAF010010398 / CCAF010010399 (trout CISHb2), XM_017450219 / LBML01016837 (catfish CISHb), EF195766 / CR589944 (zebrafish CISHb), NM_001114158 / CAAB02004909 (fugu CISHb) and XM_003441421 / MKQE01000019 (tilapia CISHb). The black and white boxes represent amino acid coding region and untranslated region within exons, respectively, and the black bars represent introns. The sizes (bp) of exons are numbered in the boxes and the intron phases are indicated above the bar.

**Fig. S23**


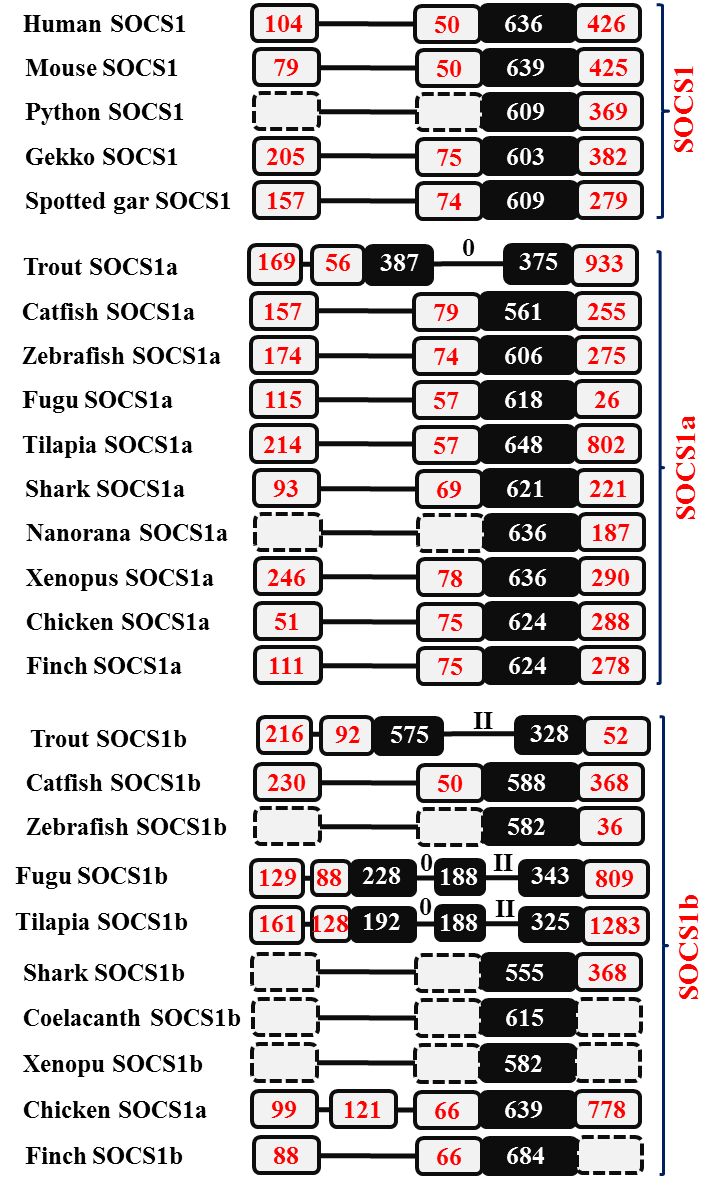


**Fig. S23 SOCS1 gene organisation across vertebrates**. The gene organisation was predicted using the Spidey programme. The accession numbers of cDNA / genomic sequences used for prediction are AB000734 / AC009121 (human SOCS1), AF120490 / CT010583 (mouse SOCS1), XM_007434469 / AEQU02124954 (python SOCS1), XM_015409927 / LNDG01050215 (gekko SOCS1), XM_015360089 / AHAT01013580 (spotted gar SOCS1), AM748721 / MSJN01099356 (trout SOCS1a), XM_017488873 / LBML01002201 (catfish SOCS1a), NM_001003467 / CABZ01037936 (zebrafish SOCS1a), DQ643957 / CAAB02005685 (fugu SOCS1a), XM_003454927 / MKQE01000018 (tilapia SOCS1a), XM_007904882 / GPS_003798271 (shark SOCS1a), XM_018570688 / JYOU01089937 (nanorana SOCS1a), BC088083 / AAMC03034580 (xenopus SOCS1a), XM_015294233 / AADN04000130 (chicken SOCS1a), XM_002197275 / ABQF01032812 (finch SOCS1a), KY387584 / MSJN01004860 (trout SOCS1b), XM_017456881 / LBML01008463 (catfish SOCS1b), JN800507 / BX901897 (zebrafish SOCS1b), XM_011612784 / CAAB02002914 (fugu SOCS1b), XM_003455977 / MKQE01000020 (tilapia SOCS1b), XM_007885846 / AAVX02048847 (shark SOCS1b), XM_006014393 / AFYH01276438 (coelacanth SOCS1b), XM_018093212 / AAMC03016537 (xenopus SOCS1b), XM_004937686 / NC_006088 (chicken SOCS1b) and XM_002192453 / ABQF01015820 (finch SOCS1b), The black and white boxes represent amino acid coding region and untranslated region within exons, respectively, and the black bars represent introns. The sizes (bp) of exons are numbered in the boxes and the intron phases are indicated above the bar. Dashed boxes indicate uncertainty of existence or size.

**Fig. S24**

**
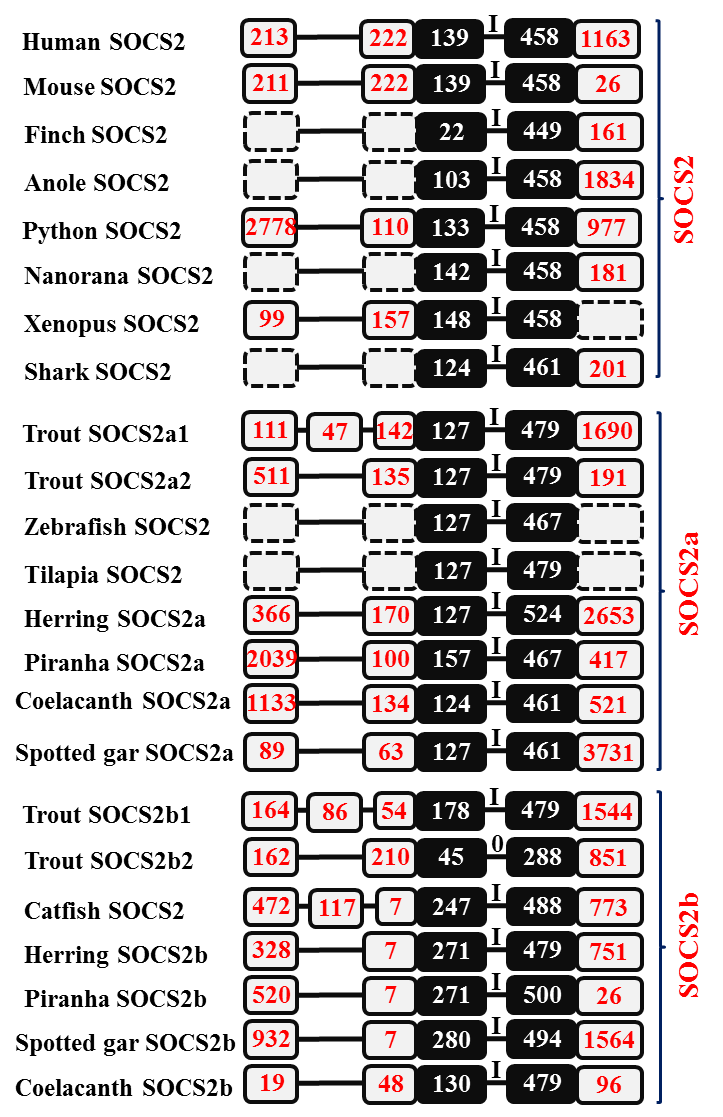
**

**Fig. S24 SOCS2 gene organisation across vertebrates.** The gene organisation was predicted using the Spidey programme. The accession numbers of cDNA / genomic sequences used for prediction are NM_001168655 / AC167222 (human SOCS2), BC106153 / NC_000076 (mouse SOCS2), XM_002187405 / ABQF01015647 (finch SOCS2), XM_003221079 / AAWZ02012484 (anole SOCS2), XM_007435720 / AEQU02137275 (python SOCS2), XM_018560239 / NW_017306710 (nanorana SOCS2), EF544587 / AAMC03011324 (xenopus SOCS2), XM_007909034 / AAVX02034644 (shark SOCS2), AM748722 / MSJN01000592 (trout SOCS2a1), KY387585 / MSJN01002587 (trout SOCS2a2), EF195761 / BX649398 (zebrafish SOCS2), KR149238 / MKQE01000009 (tilapia SOCS2), XM_012838195 / NW_012223313 (herring SOCS2a), XM_017706408 / MAUM01009537 (piranha SOCS2a), XM_006007404 / AFYH01182642 (coelacanth SOCS2a), XM_006633311 / AHAT01016211 (spotted gar SOCS2a), FR874096 / CCAF010036701 (trout SOCS2b1), FR874097 / CCAF010027937 (trout SOCS2b2), XM_017487406 and LBML01012901 (catfish SOCS2), XM_012820747 / JZKK01050640 (herring SOCS2b), XM_017698202 and MAUM01005547 (piranha SOCS2b), XM_006628341 / AHAT01014245 (spotted gar SOCS2b) and XM_005988369 / AFYH01013833 (coelacanth SOCS2b). The black and white boxes represent amino acid coding region and untranslated region within exons, respectively, and the black bars represent introns. The sizes (bp) of exons are numbered in the boxes and the intron phases are indicated above the bar. Dashed boxes indicate uncertainty of existence or size.

**Fig. S25**


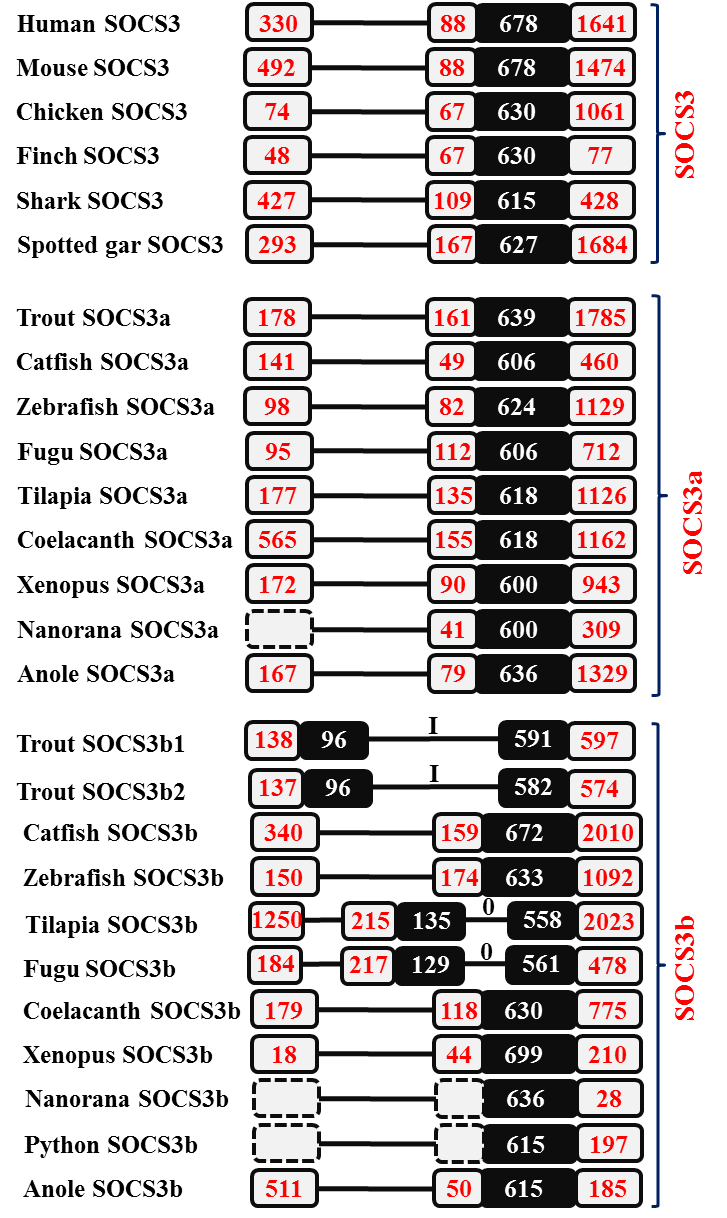


**Fig. S25 SOCS3 gene organisation across vertebrates**. The gene organisation was predicted using the Spidey programme. The accession numbers of cDNA / genomic sequences used for prediction are NM_003955 / AC061992 (human SOCS3), NM_007707 / AL591433 (mouse SOCS3), AF424806 / AADN04000355 (chicken SOCS3), XM_002196529 / ABQF01037511 (finch SOCS3), XM_007888651 / AAVX02005155 (shark SOCS3), XM_015356751 / AHAT01000365 (spotted gar SOCS3a), AM748723 / CCAF010078215 (trout SOCS3a), GU589111 / LBML01002135 (catfish SOCS3a), BC049326 / AL953847 (zebrafish SOCS3a), DQ335254 / CAAB02005766 (fugu SOCS3a), XM_003450130 / MKQE01000018 (tilapia SOCS3a), XM_005989088 / AFYH01017018 (coelacanth SOCS3a), BC075262 / AAMC03036914 (xenopus SOCS3a), XM_018573333 / JYOU01105027 (nanorana SOCS3a), XM_008104197 / AAWZ02002315 (anole SOCS3a), KY387586 / CCAF010092130 (trout SOCS3b1), KY387587 and CCAF010048864 (trout SOCS3b2), XM_017483973 / LBML01004477 (catfish SOCS3b), NM_213304 / CU855552 (zebrafish SOCS3b), XM_005471464 / MKQE01000022 (tilapia SOCS3b), XM_003979116 / NW_004073897 (fugu SOCS3b), XM_006000626 / AFYH01113324 (coelacanth SOCS3b), NM_203661 / NC_030683 (xenopus SOCS3b), XM_018573267 / JYOU01104501 (nanorana SOCS3b), XM_007439874 / AEQU02179444 (python SOCS3b) and XM_008118448 / AAWZ02025084 (anole SOCS3b). The black and white boxes represent amino acid coding region and untranslated region within exons, respectively, and the black bars represent introns. The sizes (bp) of exons are numbered in the boxes and the intron phases are indicated above the bar. Dashed boxed indicate uncertainty of existence or size.

**Fig. S26**


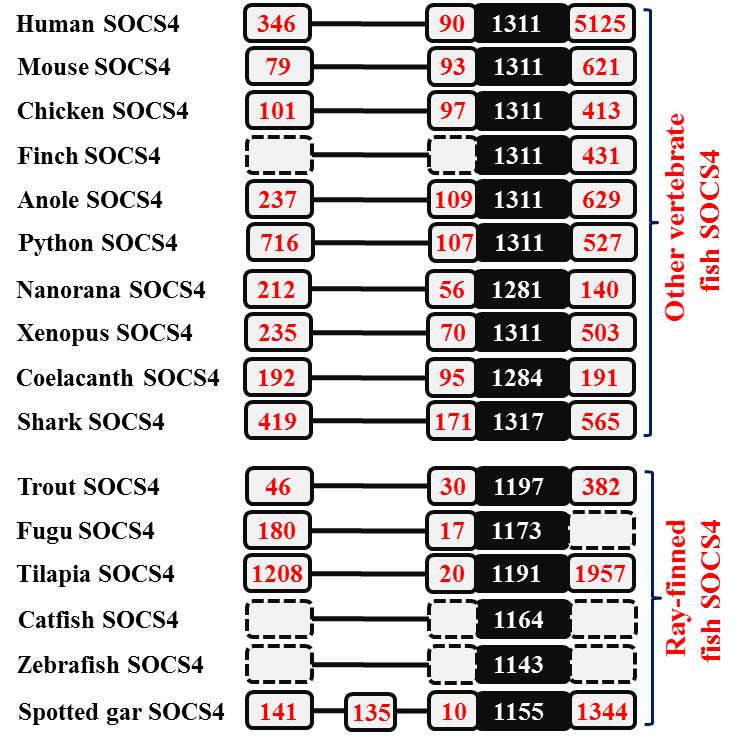


**Fig. S26 SOCS4 gene organisation across vertebrates**. The gene organisation was predicted using the Spidey programme. The accession numbers of cDNA / genomic sequences used for prediction are NM_080867 / NC_000014 (human SOCS4), NM_080843 / AC156016 (mouse SOCS4), NM_001199108 / AADN04000379 (chicken SOCS4), XM_012574073 / ABQF01004107 (finch SOCS4), XM_003225779 / AAWZ02024919 (anole SOCS4), XM_007427365 / AEQU02061981 (python SOCS4), XM_018559410 / JYOU01042137 (nanorana SOCS4), CR761536 / AAMC03032192 (xenopus SOCS4), XM_005986468 / AFYH01002849 (coelacanth SOCS4), XM_007888125 / AAVX02004635 (shark SOCS4), KY387588 / CCAF010102521 (trout SOCS4), NM_001122865 / CAAB02000042 (fugu SOCS4), XM_005458895 / MKQE01000001 (tilapia SOCS4), KM387313 / LBML01013100 (catfish SOCS4), NM_001111225 / NC_007128 (zebrafish SOCS4) and XM_015350691 / AHAT01019073 (spotted gar SOCS4), The black and white boxes represent amino acid coding region and untranslated region within exons, respectively, and the black bars represent introns. The sizes (bp) of exons are numbered in the boxes. Dashed boxes indicate uncertainty of existence or size.

**Fig. S27**

**
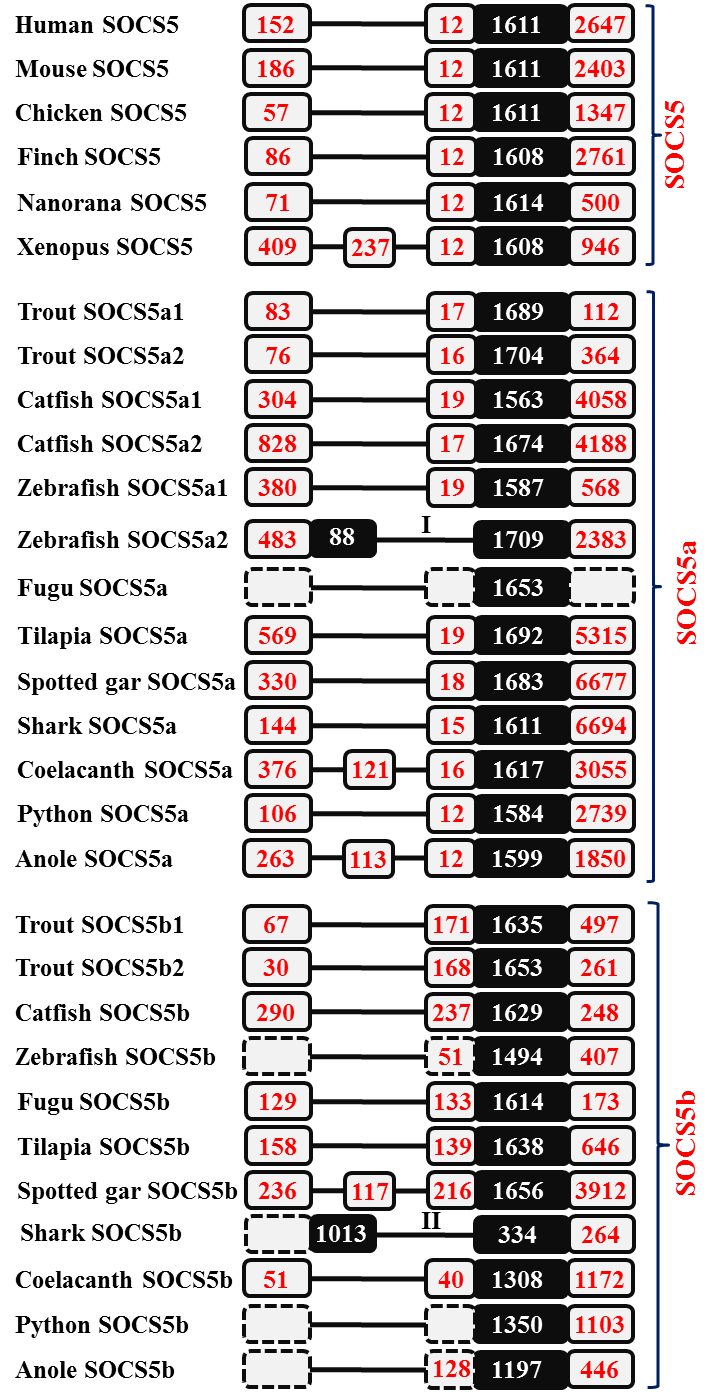
**

**Fig. S27 SOCS5 gene organisation across vertebrates**. The gene organisation was predicted using the Spidey programme. The accession numbers for cDNA / genomic sequences used for prediction are NM_014011 / NC_000002 (human SOCS5), AF033187 / AC127695.3 (mouse SOCS5), NM_001127314 / AADN04000139 (chicken SOCS5), XM_002188332 / ABQF01049187 (finch SOCS5), XM_018576289 / JYOU01011719 (nanorana SOCS5), XM_012963030 / AAMC03017945 (xenopus SOCS5), KY387589 / NC_035077 (trout SOCS5a1), KY387590 / CCAF010029944 (trout SOCS5a2), XM_017484193 / LBML01004391 (catfish SOCS5a1), XM_017463960 / NC_030418 (catfish SOCS5a2), NM_001113758 / CR385067 (zebrafish SOCS5a1), XM_005156601 / AL954180 (zebrafish SOCS5a2), EF195748 / CAAB02003445 (fugu SOCS5a), XM_013275781 / MKQE01000005 (tilapia SOCS5a), XM_015363125 / AHAT01001683 (spotted gar SOCS5a), XM_007892432 / AAVX02009726 (shark SOCS5a), XM_006012813 / NW_005822530 (coelacanth SOCS5a), XM_007441622 / AEQU02197550 (python SOCS5a), XM_016991522 / NC_014777 (anole SOCS5a), AM903341 / CCAF010050454 (trout SOCS5b1), KY387591 / CCAF010049242/ CCAF010049241 (trout SOCS5b2), XM_017491254 / LBML01017502 (catfish SOCS5b), NM_001113801 / CABZ01087561 (zebrafish SOCS5b), EF195752 / CAAB02007217 (fugu SOCS5b), XM_005474722 / MKQE01000006 (tilapia SOCS5b), XM_015341080 / AHAT01023721 (spotted gar SOCS5b), XM_007909558 / AAVX02035793 (shark SPCS5b), XM_005987802 / AFYH01009848 (coelacanth SOCS5b). XM_007434595 / AEQU02125995 (python SOCS5b) and XM_003227548 / AAWZ02029161 (anole SOCS5b). The black and white boxes represent amino acid coding region and untranslated region within exons, respectively, and the black bars represent introns. The sizes (bp) of exons are numbered in the boxes and the intron phases are indicated above the bar. Dashed boxes indicate uncertainty of existence or size.

**Fig. S28**


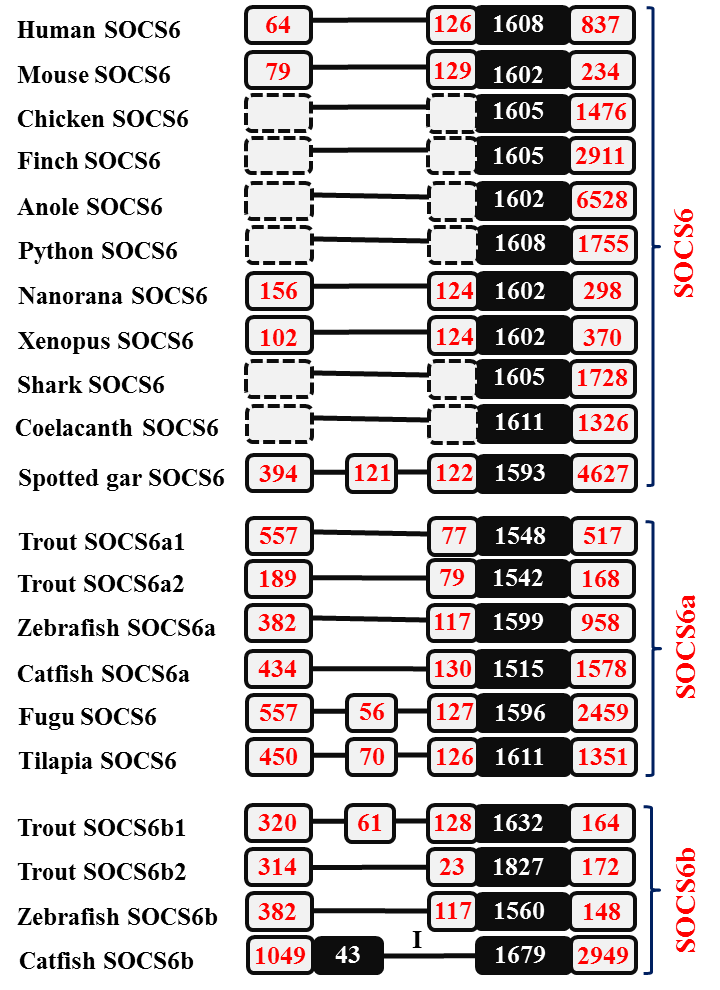


**Fig. S28 SOCS6 gene organisation across vertebrates**. The gene organisation was predicted using the Spidey programme. The accession numbers of cDNA / genomic sequences used for prediction are AF121907 / AC122398 (human SOCS6), AK031164 / AC122398 (mouse SOCS6), NM_001127312 / AADN04000136 (chicken SOCS6), XM_002195793 / ABQF01031461 (finch SOCS6), XM_003219725 / AAWZ02005710 (anole SOCS6), XM_007425323 / AEQU02043543 (python SOCS6), XM_018563180 / JYOU01056498 (nanorana SOCS6), BC135383 / AAMC03038335 (xenopus SOCS6), XM_007888015 / AAVX02004528 (shark SOCS6), XM_005995478 / AFYH01065480 (coelacanth SOCS6), XM_015353677 / AHAT01004312 (spotted gar SOCS6), AM903342 / CCAF010004893 (trout SOCS6a1), KY387592 / CCAF010023265 (trout SOCS6a2), NM_199625 / CR788254 (zebrafish SOCS6a), XM_017496398 / LBML01001110 (catfish SOCS6a), XM_011618494/ CAAB02009260 (fugu SOCS6), XM_005471782 / MKQE01000023 (tilapia SOCS6), KY387593 / CCAF010075589 (trout SOCS6b1), KY387594 / CCAF010031173 (trout SOCS6b2), XM_681949 / NC_007135 (zebrafish SOCS6b) and XM_017453704 / LBML01015661 (catfish SOCS6b). The black and white boxes represent amino acid coding region and untranslated region within exons, respectively, and the black bars represent introns. The sizes (bp) of exons are numbered in the boxes and the intron phase is indicated above the bar. Dashed boxes indicate uncertainty of existence or size.

**Fig. S29**

**
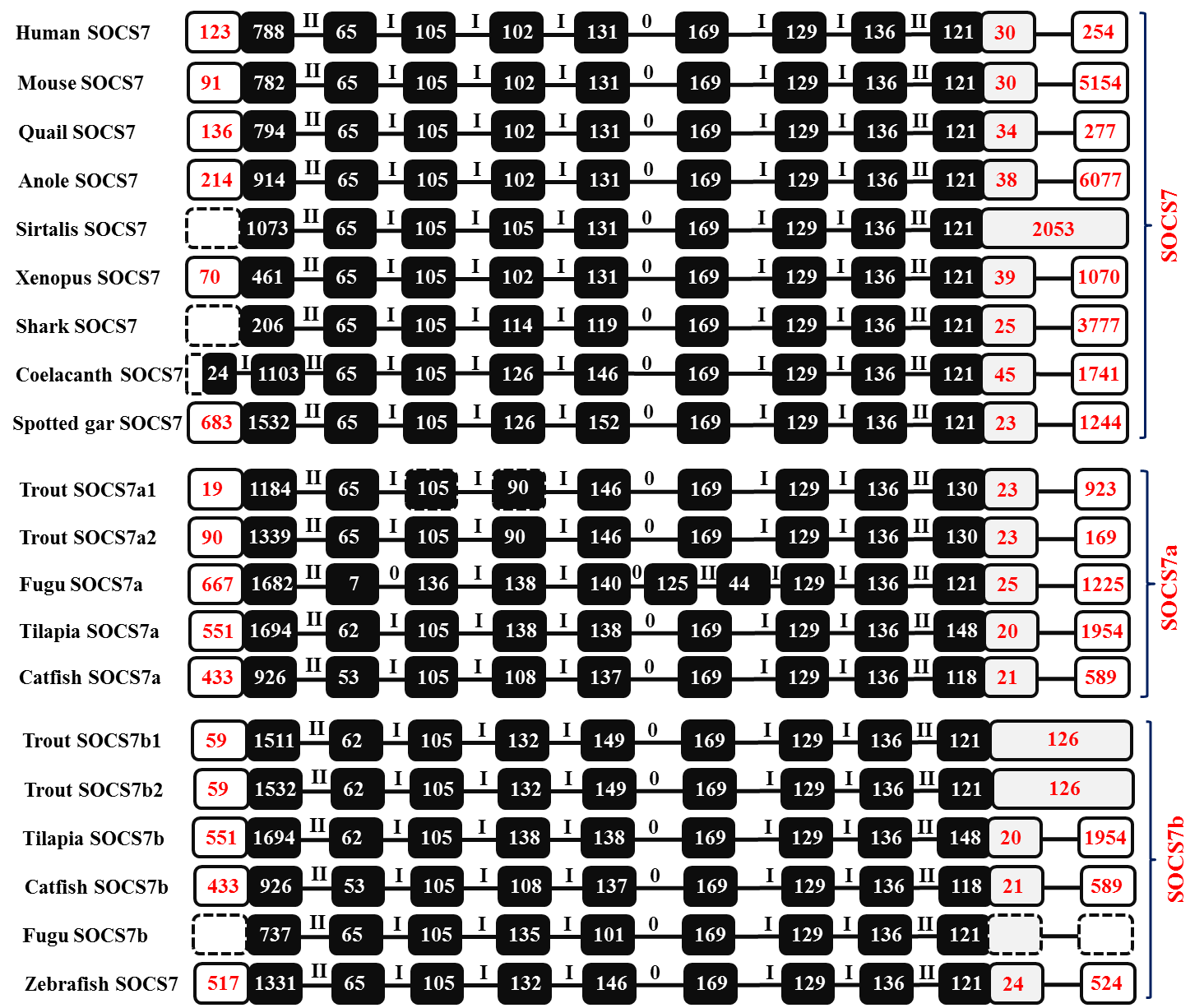
**

**Fig. S29 SOCS7 gene organisation across vertebrates**. The gene organisation was predicted using the Spidey programme. The accession numbers of cDNA / genomic sequences used for prediction are NM_014598 / AC115992 (human SOCS7), NM_138657 / AL596088 (mouse SOCS7), XM_015885974 / LSZS01001750 (quail SOCS7), XM_008113428 / AAWZ02002881 (anole SOCS7), XM_014057860 / LFLD01005363 (sirtalis SOCS7), NM_001128059 / AAMC03036285 (xenopus SOCS7), XM_007904404 / GPS_003798262.1 (shark SOCS7), XM_005990077 / NW_005819066.1 (coelacanth SOCS7), XM_006638385 / AHAT01030905 and AHAT01030907 (spotted gar SOCS7), AM903343 / NC_035089 (trout SOCS7a1), KY387595 / NC_035088 (trout SOCS7a2), XM_011604374 / NC_018894 (fugu SOCS7a), XM_005469177 / MKQE01000018 (tilapia SOCS7a), XM_017454537 / LBML01019113 (catfish SOCS7a), KY387596 / CCAF010024912 (trout SOCS7b1), KY387597 / CCAF010034763 (trout SOCS7b2), XM_005469177 / MKQE01000018 (tilapia SOCS7b), XM_017454537 / LBML01019113 (catfish SOCS7b), NM_001122863 / NW_004073730 and NW_004072415 (fugu SOCS7b), and XM_009305863 / BX004880 (zebrafish SOCS7). The black and white boxes represent amino acid coding region and untranslated region within exons, respectively, and the black bars represent introns. The sizes (bp) of exons are numbered in the boxes and the intron phases are indicated above the bar. Dashed boxes indicate uncertainty of existence or size.

**Fig. S30**


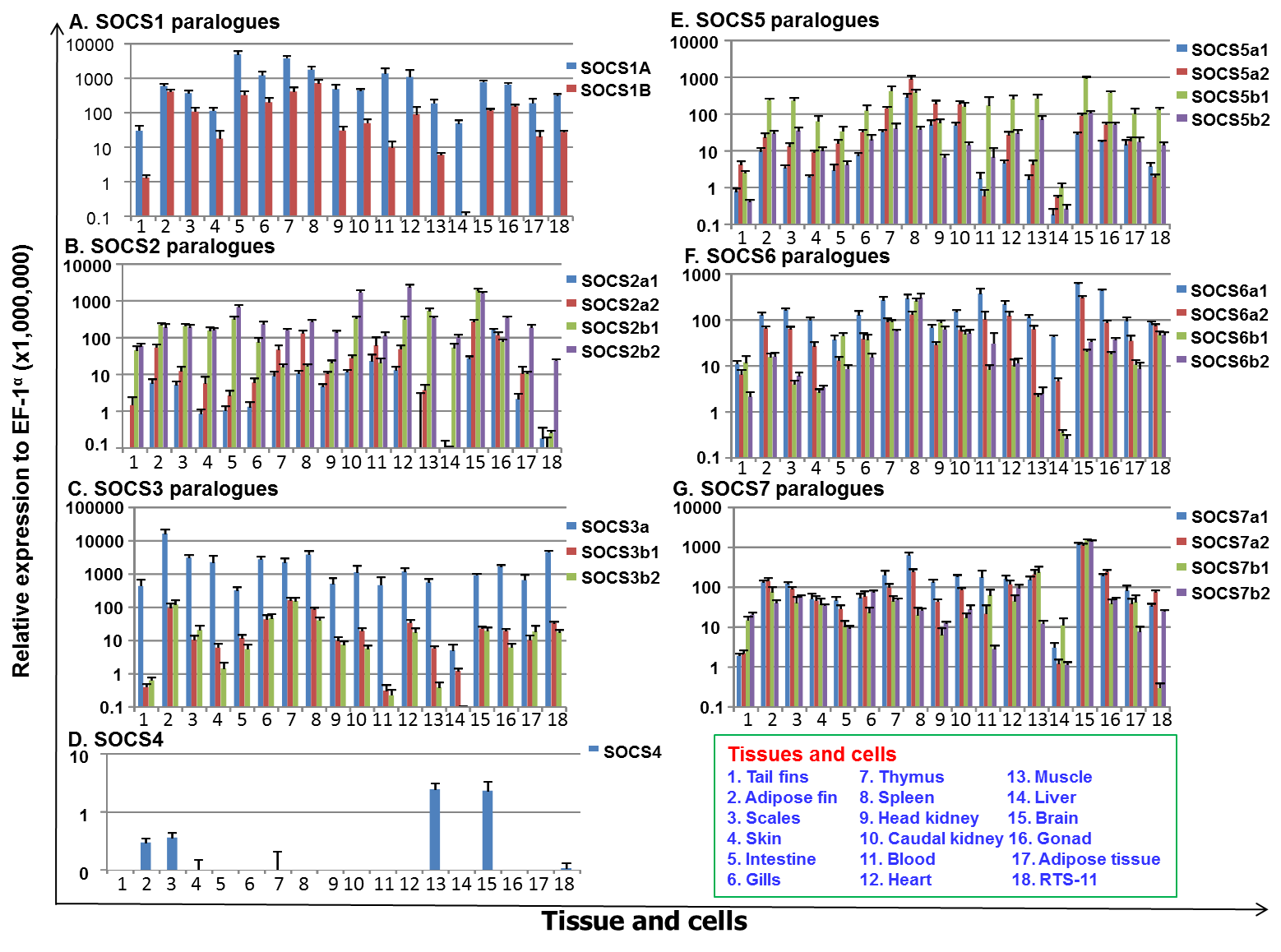


**Fig. S30 Comparative expression of trout paralogues of SOCS1 (A), SOCS2 (B), SOCS3 (C), SOCS4 (D), SOCS5 (E), SOCS6 (F) and SOCS7 (G) *in vivo* in tissues and in RTS-11 cells.** The transcript expression level of the SOCS genes was determined by real time RT-PCR in 17 tissues from six fish and in un-stimulated RTS-11 cells. The transcript level was calculated using a serial dilution of references that contained equal molar amounts of the probes for each gene and was normalized against the expression level of EF-1α. The expression levels were relative to EF-1α expression (multiplied by 1,000,000). The results are presented as averages +SEM (N=6).

**Table S1 Primers used for PCR cloning and real-time RT-PCR analysis of gene expression of rainbow trout SOCS gene family members**

| Gene | Primer name | Sequence (5’ to 3’) | Application |
| --- | --- | --- | --- |
| SOCS1a | SOCS1A-F | GCTAGGAGTGGAGGAGGCTCACA | Real-time PCR |
|  | SOCS1A-R | AGAGGCAGGGGAAAGAGTGCTC | Real-time PCR |
| SOCS1b | S1B1F1 | ACGGTGACAGTAATGTAATGGTGTGA | PCR cloning |
|  | S1B1R1 | CAGTCTGTTGAAACGGAAGTGGTG | PCR cloning |
|  | SOCS1B1-F | AGCTTCCTCATCAGGGACAGCA | Real-time PCR |
|  | SOCS1B1-R | GCCTATGGGATGACTCCACGTAGAA | Real-time PCR |
| SOCS2a1 | SOCS2A1-F | CAAAAACAAGACGCGTGGGAGAA | Real-time PCR |
|  | SOCS2A1-R | CGTCGGAATCTACAACCCGTGACA | Real-time PCR |
| SOCS2a2 | S2A2F1 | GGGTTTAATGGGGATTTGGTTAGTC | PCR cloning |
|  | S2A2R1 | GGATTAGATGACTGACTTGGCTCTATAAGC | PCR cloning |
|  | SOCS2A2-F | CCACTCGCAAATAGGACGATTCATTT | Real-time PCR |
|  | SOCS2A2-R | TCATCGGAGTCTACAACTCGCGACT | Real-time PCR |
| SOCS2b1 | SOCS2B1-F | CTCACCTTCAGGAGTCAGGCTGGTA | Real-time PCR |
|  | SOCS2B1-R | ACATGCCAGTGGCGTGCTCTATAC | Real-time PCR |
| SOCS2b2 | SOCS2B2-F | TGTCTCATCTTCGGGAGTCAGTGTTC | Real-time PCR |
|  | SOCS2B2-R | CATGCCAGTGCCATGCTCTATGT | Real-time PCR |
| SOCS3a | SOCS3A-F | CACAGAGAAACCGTTAAAAGGACTATCC | Real-time PCR |
|  | SOCS3A-R | AAGGGGCTGCTGCTCATGAC | Real-time PCR |
| SOCS3b1 | S3B1F1 | ACCTTCTTCTCTCTCTCCCTCCTG | PCR cloning |
|  | S3B1R1 | CGACACCACAAACACTTTCACTCTG | PCR cloning |
|  | SOCS3B1-F | CACATGCACACTACCAACAGGTGCTGT | Real-time PCR |
|  | SOCS3B1-R | GGGGCATGTGTGTGCAGCA | Real-time PCR |
| SOCS3b2 | S3B2F1 | CCTCCATCACAACTCACCTCCTC | PCR cloning |
|  | S3B2R1 | GACACCATGCACACAAACACATTC | PCR cloning |
|  | SOCS3B2-F | CACGCACACTACCAACAGGTGCTGT | Real-time PCR |
|  | SOCS3B2-R | GGGCGTGTGCGTGCAGTG | Real-time PCR |
| SOCS4 | S4F1 | CATGGACATGGAGATTACGTTGTC | PCR cloning |
|  | S4R1 | CTATTACCTCACACAGTGAGTCCGTG | PCR cloning |
|  | SOCS4-F | CGTTGTCTCTCTAAAACTATTTTTAAAAGCACAGAG | Real-time PCR |
|  | SOCS4-R | AGGTGGAACGCGCTCCTTG | Real-time PCR |
| SOCS5a1 | S5A1F1 | GGGATATGTGCCTGACTTGTCTTG | PCR cloning |
|  | S5A1R1 | TCCCTGCCTGACTGAAAAGTGCCA | PCR cloning |
|  | SOCS5A1-F | GTGTTTATAAAAGACAAAAGGGTCTTGGTCTC | Real-time PCR |
|  | SOCS5A1-R | GACGTCGTCCCCCAGTAACCAT | Real-time PCR |
| SOCS5a2 | S5A2F1 | GGGATATGTGCCTGACTTAAGCCA | PCR cloning |
|  | S5A2R1 | CATGCTGTGGGATCAGGCAG | PCR cloning |
|  | SOCS5A2-F | CTGGTTTAAAAAGACAAAAGGGGCTTG | Real-time PCR |
|  | SOCS5A2-R | TCCTCATTGTCTTTTCTGTCTGTGGTTC | Real-time PCR |
| SOCS5b1 | SOCS5B1-F | GCAAGGAATACTGTTTTTTTTAAGCCTCAAG | Real-time PCR |
|  | SOCS5B1-R | GACGTATGGCCTTTTTTCTTTCCTCTTC | Real-time PCR |
| SOCS5b2 | S5B2F1 | AGCTTTTGGAGAGAACAACGAAGTT | PCR cloning |
|  | S5B2R1 | GAGAAAAATGGGTGAATGTGCATCAG | PCR cloning |
|  | SOCS5B2-F | GAACAACGAAGTTTTTATGCCCCAAGT | Real-time PCR |
|  | SOCS5B2-R | CAGCTTGCTCATGGGACGTCTG | Real-time PCR |
| SOCS6a1 | SOCS6A1-F | GTTGAAAAACATACTGTAGAAAGGGATGACA | Real-time PCR |
|  | SOCS6A1-R | TCCTCATGATTGAAGTCGTTGCG | Real-time PCR |
| SOCS6a2 | S6A2F1 | CGTGTGGGATCTCTAAAGCCAGTTAG | PCR cloning |
|  | S6A2R1 | CTTCAATCCTTGGCTTTGAGGTG | PCR cloning |
|  | SOCS6A2-F | GTTTTTAACAGAGGATGACAGGGATGACT | Real-time PCR |
|  | SOCS6A2-R | CTTCTCCTCGTGATCAAAATCGTTACA | Real-time PCR |
| SOCS6b1 | S6B1F1 | GGAGAGAAGAAGAGGGCGACAGA | PCR cloning |
|  | S6B1R1 | CACTACATACAGACATCCCTGGACAC | PCR cloning |
|  | SOCS6B1-F | TGCAGATGTTCATCTAACGAAAGCAAAG | Real-time PCR |
|  | SOCS6B1-R | TTGGCAGGGGCGGCTACTCT | Real-time PCR |

Table S1 Continued.

| SOCS6b2 | S6B2F1 | GCAAGAGAGAGAGGAAGAGTCCGTC | PCR cloning |
| --- | --- | --- | --- |
|  | S6B2R1 | CGGAATAGGGTTCCATTTTGGAC | PCR cloning |
|  | SOCS6B2-F | GCAGATGGTAATCTAACGAAGGGATGAGT | Real-time PCR |
|  | SOCS6B2-R | CCCTCCTTCTTCCTTGCCCTTGT | Real-time PCR |
| SOCS7a1 | SOCS7A-F | GGACCAGGACACAGGGAGCAAACT | Real-time PCR |
|  | SOCS7A-R | GAAGGCAGGACCGAAGGCCAC | Real-time PCR |
| SOCS7a2 | S7BF1 | GTTTCTGATGCGCCTCGACCT | PCR cloning |
|  | S7BR1 | AGCGAGATTGTCCCCAATCAGT | PCR cloning |
|  | SOCS7B-F | CAGACTTGGACACAGGGAGCAAACA | Real-time PCR |
|  | SOCS7B-R | GAAGGCAGGACCGAAGGCCAA | Real-time PCR |
| SOCS7b1 | S7B1F1 | GCGGTAGCTATGTGAAGCGTAGAAC | PCR cloning |
|  | S7B1R1 | CCAAAGTCAAGAGAAGGGCTAGGTA | PCR cloning |
|  | SOCS7B1-F | CGCTGCCCAAACCACTGATCA | Real-time PCR |
|  | SOCS7B1-R | CCAGTTACGTCTGAGACTCCGCCTCTTT | Real-time PCR |
| SOCS7b2 | S7B2F1 | GCGGTAGCTATGTGAAGCGTTGAAA | PCR cloning |
|  | S7B2R1 | TCAAAGTCAAGAGAAGGCATGGTC | PCR cloning |
|  | SOCS7B2-F | CGCTGCCCAAACCACTGATCA | Real-time PCR |
|  | SOCS7B2-R | CAGTTACGTCTGAGACTCGGCCACTTG | Real-time PCR |
| CISHa1 | CISH-A1-F | CATTCTACCTTGATACCTCAGGCTGGT | Real-time PCR |
|  | CISH-A1-R | CCTGCTGCACCTTCCTCCC | Real-time PCR |
| CISHa2 | CISH-A2-F | TCTTCTACCTTGATACCTCAGGCTGGT | Real-time PCR |
|  | CISH-A2-R | CCTTGCCCTTCTGTACCTTCCTTGT | Real-time PCR |
| CISHb1 | CISH-B1-F | GAATGATGGTGAGAGAGGAGATTTGTGT | Real-time PCR |
|  | CISH-B1-R | GACCCCCAGTACCAACCTGAGTT | Real-time PCR |
| CISHb2 | CISH-B2-F | GATCATGGTGAGAGAGGAGGGTCA | Real-time PCR |
|  | CISH-B2-R | GACCCCCAGTACCAACCTGAGTT | Real-time PCR |
| EF-1α | EF-1α-F | CAAGGATATCCGTCGTGGCA | Real-time PCR |
|  | EF-1α-R | ACAGCGAAACGACCAAGAGG | Real-time PCR |

**Table S2 The amino acid number and identity (top right)/similarity (bottom left) of CISHa, CISHb and CISH.** The accession number for each protein sequence is shown in Fig. 2.

**Table S3 The amino acid number and identity (top right)/similarity (bottom left) of SOCS1a, SOCS1b and SOCS1.** The accession number for each protein sequence is shown in Fig. 2.

**Table S4 The amino acid number and identity (top right)/similarity (bottom left) of SOCS2a, SOCS2b and SOCS2.** The accession number for each protein sequence is shown in Fig. 2.

**Table S5 The amino acid number and identity (top right)/similarity (bottom left) of SOCS3a, SOCS3b and SOCS3.** The accession number for each protein sequence is shown in Fig. 2.

**Table S6 The amino acid number and identity (top right)/similarity (bottom left) of SOCS4 homologues.** The accession number for each protein sequence is shown in Fig. 3.

**Table S7 The amino acid number and identity (top right)/similarity (bottom left) of SOCS5a, SOCS5b and SOCS5.** The accession number for each protein sequence is shown in Fig. 3.

**Table S8 The amino acid number and identity (top right)/similarity (bottom left) of SOCS6a, SOCS6b and SOCS6.** The accession number for each protein sequence is shown in Fig. 3.

**Table S9 The amino acid number and identity (top right)/similarity (bottom left) of SOCS7a, SOCS7b and SOCS7.** The accession number for each protein sequence is shown in Fig. 3.

**Table S10 Summary of intron/exon organisation of the vertebrate SOCS gene family.** The numbers of genes analysed and the numbers of genes with changed gene organisation are shown.

|  | 2R vertebrates | | 3R fish | | 4R rainbow trout | |
| --- | --- | --- | --- | --- | --- | --- |
|  | No. of genes analysed | No. of genes changed | No. of genes analysed | No. of genes changed | No. of genes analysed | No. of genes changed |
| CISH | 8 | 0 | 8 | 3 | 4 | 1 |
| SOCS1 | 15 | 1 | 8 | 2 | 2 | 2 |
| SOCS2 | 13 | 0 | 7 | 1 | 4 | 3 |
| SOCS3 | 15 | 0 | 8 | 2 | 3 | 2 |
| SOCS4 | 11 | 1 | 4 | 0 | 1 | 0 |
| SOCS5 | 16 | 5 | 10 | 1 | 4 | 0 |
| SOCS6 | 11 | 1 | 6 | 3 | 4 | 1 |
| SOCS7 | 9 | 2 | 7 | 1 | 4 | 2 |
| Total | 98 | 10 | 58 | 13 | 26 | 11 |
| % of change | 10/98=10.2% | | 13/58=22.4% | | 11/26=42.3% | |

**Table S11 The p-value of a paired samples T test of the expression levels of SOCS paralogue pairs in 17 tissues and RTS-11 cells (N=6).** The arbitrary unit measurement in Fig. 7 was log2 transformed with negative numbers (AU < 1) converted to 0 before statistical analysis. The numbers are in red when p ≤ 0.05. NT, not determined due to low level expression.

**Table S12 The Spearman's rho correlation coefficient (R) between the the expression levels of SOCS gene family members in 17 tissues and RTS-11 cells (N=108).** The expression data was transformed as in Table 11. The numbers are in black when R > 0.8. The genes with exon insertion in the 5’-UTR and intron loss in the 3’-UTR and a low R between paralogues are highlighted in green.

**Note:**

* Correlation is significant at the 0.05 level (2-tailed).

** Correlation is significant at the 0.01 level (2-tailed).
